# Genome-wide analyses of genes encoding FK506-binding proteins reveal their involvement in abiotic stress responses in apple

**DOI:** 10.1186/s12864-018-5097-8

**Published:** 2018-09-25

**Authors:** Qinglong Dong, Ke Mao, Dingyue Duan, Shuang Zhao, Yanpeng Wang, Qian Wang, Dong Huang, Chao Li, Changhai Liu, Xiaoqing Gong, Fengwang Ma

**Affiliations:** State Key Laboratory of Crop Stress Biology for Arid Areas/Shaanxi Key Laboratory of Apple, College of Horticulture, Northwest A & F University, Yangling, 712100 Shaanxi China

**Keywords:** Genome-wide, Apple, FKBP gene family, Expression analysis, Salt stress, Drought stress

## Abstract

**Background:**

The FK506-binding proteins (FKBPs) play diverse roles in numerous critical processes for plant growth, development, and abiotic stress responses. However, the FKBP gene family in the important fruit crop apple (*Malus* × *domestica* Borkh.) has not been studied as thoroughly as in other species. Our research objective was to investigate the mechanisms by which apple FKBPs enable apple plants to tolerate the effects of abiotic stresses.

**Results:**

Using bioinformatics-based methods, RT-PCR, and qRT-PCR technologies, we identified 38 FKBP genes and cloned 16 of them in the apple genome. The phylogenetic analysis revealed three major groups within that family. The results from sequence alignments, 3-D structures, phylogenetics, and analyses of conserved domains indicated that apple FKBPs are highly and structurally conserved. Furthermore, genomics structure analysis showed that those genes are also highly and structurally conserved in several other species. Comprehensive qRT-PCR analysis found various expression patterns for *MdFKBP*s in different tissues and in plant responses to water-deficit and salt stresses. Based on the results from interaction network and co-expression analyses, we determined that the pairing in the MdFKBP62a/MdFKBP65a/b-mediated network is involved in water-deficit and salt-stress signaling, both of which are uniformly up-regulated through interactions with heat shock proteins in apple.

**Conclusions:**

These results provide new insight for further study of FKBP genes and their functions in abiotic stress response and multiple metabolic and physiological processes in apple.

**Electronic supplementary material:**

The online version of this article (10.1186/s12864-018-5097-8) contains supplementary material, which is available to authorized users.

## Background

Immunophilins are cellular receptor proteins for immunosuppressive drugs that can be combined with FK506, Cyclosporine-A (CsA), and rapamycin [1, 2]. As agents of immunosuppression, immunophilins are divided into two categories: cyclophilins, which can bind to CsA; and FK506-binding proteins (FKBPs), which can bind to FK506 and rapamycin [2–4]. The FKBPs belong to a superfamily for peptidyl-prolyl *cis-trans*isomerase (PPIase, EC 5.1.2.8), which catalyzes the rotation of the peptide bond that immediately precedes a proline residue between the *cis* and *trans* configurations; family members are present in all organisms and almost all subcellular compartments, from yeasts to humans [1, 2, 5, 6].

All FKBPs contain at least one FK506-binding domain (FKBd) of approximately 110 amino acids that provides the active site for PPIase catalysis and the receptor site for proline and proline analogues [1, 2, 7]. In FKBPs, the formation of a well-conserved tertiary structure of FKBd primarily contains six anti-parallel beta sheets that form a concave surface and hydrophobic sidechains; a short alpha helix; and several solvent-exposed loops that interact with substrates bound at the FKBP active site [1, 2, 7]. Members of FKBP families in plants range significantly in size, from single-domain (SD) isoforms that comprise a single FKBd to multiple-domain (MD) proteins that contain up to three FKBds, along with a tetratricopeptide repeat (TPR), C-terminal calmodulin-binding domains, or a coiled–coil domain [1, 2, 6–8]. In plants, FKBPs have been isolated from the mitochondrion, endoplasmic reticulum, cytosol, and nucleus [9–12]. However, most FKBP subcellular locations are expressed in the chloroplast thylakoid [13–15].

Over the past 20 years, researchers have reported that plant FKBPs perform diverse functions in numerous critical processes for growth and development, including signal transduction, hormonal pathways, stress responses, photosynthesis, and gene transcription [1, 2, 5–8, 16–18]. Both AtFKBP12 from *Arabidopis thaliana* (hereafter *Arabidopsis*) and PwFKBP12 (from *Picea wilsonii*) are involved in cell cycle regulation and embryo development, and they also participate in controlling the direction of pollen tube growth through their relationship with FKBP12 interacting protein 37 kD (AtFIP37) and a putative CCAAT-binding transcription factor protein PwHAP5, respectively [19–21]. In the green algae *Chlamydomonas reinhardtii* and maize (*Zea mays*; Zm), FKBP12 and rapamycin form a complex that inhibits TOR kinase, an important regulator of plant germination and development [22, 23]. Overexpression of *Polytrichastrum alpinum PaFKBP12* enhances tolerance to heat, salt, and drought stresses in *Arabidopsis* [24]. The FKBP62 (ROF1) and FKBP65 (ROF2) proteins from *Arabidopsis* and the closely related FKBPs, FKBP73, and FKBP77 from wheat (*Triticum aestivum*) are induced by malondialdehyde (MDA) treatment, wounding, and NaCl stress, all of which up-regulate the expression of genes involved in abiotic stress responses [1, 7, 25, 26]. Moreover, AtFKBP20–2 in the chloroplast thylakoid lumen is required for accumulation of the PSII supercomplex in *Arabidopsis* [16]. The AtFKBP72 (PAS1) protein helps control cell proliferation, and *pas1* mutant plants show severe developmental defects [27]. Furthermore, AtFKBP42 (TWD1) modulates the activities of P-glycoproteins, influencing auxin efflux from the root apoplast into the cytoplasm by interacting with the B family of ABC transporter (ABCB), and also regulating brassinosteroid signaling through an interaction with the receptor kinase BRASSINOSTEROID-INSENSITIVE 1 (BRI1) [28–32]. Although *twd1* mutant plants exhibit disoriented growth for all organs, they still develop fertile flowers and seeds [28]. Whereas members of the FKBP family have conserved some of their basic functions in higher eukaryotes, other biological functions are unique to each member, such that one member cannot completely compensate for the absence of another [33]. Therefore, researchers must characterize each member independently to uncover those unique functions.

In many plant species, increasing numbers of FKBP gene family members have been identified and characterized based on the highly conserved FK506-binding domain reported for *Arabidopsis* (At) [34], rice (*Oryza sativa*; Os) [8, 35, 36], maize [37], grapevine (*Vitis vinifera*; Vv) [3], strawberry (*Fragaria ananassa*; Fa) [5], and peach (*Prunus persica*; Pp) [6]. Although considerable details are known about this family in several species because it has been subjected to extensive genomic analysis; members in apple (*Malus* × *domestica* Borkh.) have not been as thoroughly investigated. Nevertheless, recent completion of the draft genome sequence for apple has enabled genome-wide analyses of FKBP genes in that species [38–40]. Here, this work identified the FKBP genes in apple and examined their FK506-binding domain, protein and gene structures, conserved domains, phylogenetic relationships, chromosomal locations, *cis*-acting elements, and expression patterns for *MdFKBP*s cloned from various tissues and in response to water deficits and salt stress. To our knowledge, this is the first comprehensive study of the FKBP gene family in apple. These results could facilitate future investigations into the functions of these genes in apple growth, developmental processes, and abiotic stress responses, and would lay a solid foundation for efforts to introduce improved apple cultivars.

## Methods

### Identification of apple FKBP genes

The database of the *Arabidopsis* FKBP family was downloaded from the TAIR website (http://www.arabidopsis.org/) [34]. As query sequences for BlastP (http://www.rosaceae.org/tools/ncbi_blast) against predicted apple proteins, we used 23 *Arabidopsis* FKBP proteins and the consensus protein sequences of the FK506-binding domain Hidden Markov Model (HMM) profile (FKBP_C, PF00254) from the Pfam database (http://pfam.xfam.org/family/PF00254). We then searched all of those FKBP sequences against the apple genome database (https://www.rosaceae.org/gb/gbrowse/malus_x_domestica/) with HMMER v3.0 and BlastP [41]. Confirming the reliability of those protein sequences ensured that the FK506-binding domain was present in each candidate MdFKBP protein. For this, we used the Pfam database (http://pfam.sanger.ac.uk/search) and NCBI-Conserved Domain Search (NCBI-CDD; http://www.ncbi.nlm.nih.gov/Structure/cdd/wrpsb.cgi) [41].

### Sequence logo and structure modeling

Sequence logos for the FK506-binding domain in 22 FKBP12 genes and the TPR domain in 25 FKBP42 genes were generated by the application WebLogo (http://weblogo.threeplusone.com) [42]. The web server SWISS-MODEL (http://swissmodel.expasy.org/) [43] was used for modeling and predicting the homology of protein structures for MdFKBP12, MdFKBP42, MdFKBP62, and MdFKBP72. The proposed 3-D structure was modelled on the original NMR structure in PDB ID: 5HWB [44], 2IF4 [45], 1KT1 [46], and 3JYM [47], and RasTop 2.2 software (http://www.geneinfinity.org/rastop/) was used to present that model [41].

### Sequence alignment and phylogenetic analysis

Multiple sequence alignments of 36 MdFKBP protein sequences were performed for using DNAMAN 6.0.3.99 with its default parameters. The phylogenetic tree for the MdFKBP gene family was constructed with MEGA 5.2 software (www.megasoftware.net) and the Neighbour-Joining (NJ) method, utilizing the amino acid sequences for those 36 proteins as well as FKBP12 proteins from other species. The following parameters were used in the NJ method: bootstrap (1000 replicates), complete deletion, and amino:p-distance. Protein–protein interactions were determined using STRING 10.0 (http://string-db.org/), setting the confidence score at > 0.9 [48, 49].

### Analyses of intron-exon structure, genome distribution, and gene duplications

Genomic sequences (apple v1.0), gene distributions on chromosomes, and genome locations for the FKBP genes in apple were downloaded from the apple genome database (Additional file 1: Table S1). Data for intron-exon distribution of the FKBP12, FKBP42, FKBP62, and FKBP72 genes in various species were downloaded from the resource PLAZA 3.0 (http://bioinformatics.psb.ugent.be/plaza/) (Additional file 2: Table S2) [50]. The MdFKBP genes were mapped onto chromosomes by using MapInspect (www.plantbreeding.wur.nl/UK/software_mapinspect.html), a locational software for identifying chromosomal positions [41]. We followed the method of Hu and Liu [51] for investigating segmental and tandem duplication events.

### Prediction of *cis*-acting elements in promoters

To examine the putative *cis*-acting elements in the promoters of apple FKBP genes, we isolated sequences that were 1500 bp upstream of the translational start codon, using the contig sequences of that genome and PCR amplification. Details for the promoters used here are listed in Table 2 and Additional file 3: Table S3. Possible *cis*-acting elements in those promoters were then predicted according to the Plant CARE database (http://bioinformatics.psb.ugent.be/webtools/plantcare/html/).

### Plant materials and treatments

For gene cloning and expression analysis, young roots, stems, and leaves, as well as flowers and mature fruit (70 mm, red peel, 150 d after bloom) were collected from apple plants that were five-years-old after bud-grafting. The scion was *Malus domestica* ‘Golden Delicious’ and the rootstock was *M. hupehensis*. Samples used for examining the effects of water deficits and NaCl stress were harvested from plants 3 months after bud-grafting was performed with ‘Golden Delicious’ scions and *M. hupehensis* rootstocks. These grafted plants were grown in pots (height, 320 mm; diameter, 300 mm) in a greenhouse and treatments began when the plants were approximately 500 mm tall. To induce a water deficit, irrigation was withheld from certain plants for up to 8 d while the designated control plants continued to receive normally scheduled irrigation [52]. For salt-stress treatment, plants were irrigated for 2 d with a 200 mM NaCl solution [52]. The sampling schedule involved harvesting mature leaves at the middle nodes on Days 0, 4, and 8 of the water deficit; or on Days 0, 1, and 2 during salt treatment. All tissues were frozen immediately in liquid N_2_ and stored at − 80 °C.

### Cloning of *MdFKBP*s and qRT-PCR analysis of expression

Total RNA was extracted from previously frozen tissues according to the hot borate method [53]. Two micrograms of total RNA were used to synthesize first-strand cDNA. For cloning *MdFKBP*, complete open reading frames (ORFs) were obtained by RT-PCR, using specific primers listed in Additional file 4: Table S4. The 5’- and 3’-untranslated regions (UTRs) were obtained with a Rapid Amplification for cDNA Ends kit (TaKaRa, Dalian, China). For the qRT-PCR assays, reverse-transcription was performed with 1 μg of total RNA from each sample, followed by PCR-amplification of 1 μL of the product. We conducted the qRT-PCR assays in 20-μL reaction mixtures that contained 10 μL of SYBR® Premix Ex Taq™ (TaKaRa), and used an iQ5 instrument (Bio-Rad, Hercules, CA, USA) as described before [54]. Thermal-cycling included an initial 3 min at 95 °C; then 40 cycles of 10 s at 95 °C, 30 s at 58 °C, and 15 s at 72 °C; followed by 3 min at 72 °C and then 81 cycles of 7 s each, increasing by an increment of 0.5 °C from 55 °C to 95 °C. Three biological replicates were tested in each assay, and △Ct values were calculated by using *MdMDH* as our endogenous control [55]. Relative quantification values were calculated according to the 2^-△△Ct^ method [56] and dissociation curve analysis was performed for determining the specificity of the amplifications. Heat maps of MdFKBP family members were constructed and hierarchical clustering was conducted with the MeV v4.8.1 software package [57].

### Statistical analysis

All data were analyzed with IBM SPSS Statistics v20. One-way ANOVA and Tukey’s tests were used to compare the results from abiotic stress treatments against their respective controls. Values were considered significantly different at *p* < 0.05.

## Results

### Identification and annotation of apple FKBP genes

To identify the genes in the apple genome that encode FK506-binding proteins, we conducted a BlastP of the apple genome database and identified 42 putative FKBP family genes in that genome. We then used the Pfam and NCBI-CDD databases to verify that all of them were FKBP members by searching for the FK506-binding domain in the amino acid sequences encoded by all 42 genes. From this, we confirmed the identity of 36 typical apple FKBP genes in the original dataset (Table 1). In addition to those 36, we found four genes with partial FKBP ORFs in the apple genome (Additional file 5: Table S5). However, we were unable to analyze those genes further because of their incomplete ORFs. Another two were identified as TIG genes. However, unlike other species, the apple TIG genes do not contain the FKBP_C domain. We classified these 36 apple FKBP genes as either SD members with an FKBP catalytic domain, or MD members with a TPR domain. Among those 36, 29 genes were characterized as SD members, three genes (MDP0000205111, MDP0000141863, and MDP0000182579) were predicted to encode proteins containing two FKBP_C domains, and four (MDP0000175388, MDP0000292276, MDP0000858936, and MDP0000146478) contained three FKBP_C domains (Table 1).

**Table 1:** MdFKBP genes in apple genome

Gene name	Gene ID^a^	Similarity (%)^b^	FKBP_C domain number	Protein length (AA)	MW (kDa)	Theoretical pI	Chromosome Location	SubLocation (WoLF/TargetP)^c^
*MdFKBP12*	MDP0000201315	79	1	112	12.03	7.18	chr4:1045355..1047625	Chloroplast
*MdFKBP13*	MDP0000206611	64	1	170	18.31	10.05	chr9:31699221..31701106	Chloroplast/_
*MdFKBP15–1*	MDP0000119443	77	1	143	15.62	7.05	chr10:15393041..15395572	Extracellular/_
*MdFKBP15–2*	MDP0000252743	74	1	143	15.76	6.03	chr5:17596963..17599596	Extracellular/_
*MdFKBP16–2*	MDP0000255282	79	1	236	24.79	8.94	chr10:19387108..19390624	Chloroplast/_
*MdFKBP16–3*	MDP0000257937	67	1	232	24.75	7.65	chr4:1049663..1053844	Chloroplast/_
*MdFKBP16–4*	MDP0000136937	75	1	235	25.19	9.34	chr12:12820631..12827383	Chloroplast/_
*MdFKBP17–1*	MDP0000225037	83	1	214	23.33	8.48	chr5:10020089..10021794	Chloroplast/_
*MdFKBP18*	MDP0000246192	69	1	243	26.15	9.74	chr6:804241..806170	Chloroplast/_
*MdFKBP19*	MDP0000316685	74	1	244	26.89	9.49	chr4:7139029..7146029	Chloroplast/_
*MdFKBP20-1a*	MDP0000250600	82	1	188	20.13	7.92	chr1:18126709..18130908	Nuclear/_
*MdFKBP20-1b*	MDP0000295893	81	1	188	20.15	6.63	chr1:16425122..16426609	Nuclear/_
*MdFKBP20–2a*	MDP0000260154	75	1	999	108.42	8.65	chr2:23076779..23083272	Plasmid/_
*MdFKBP20–2b*	MDP0000244902	75	1	999	108.42	8.65	chr2:23092712..23099197	Plasmid/_
*MdFKBP20–2c*	MDP0000252922	77	1	1016	110.52	8.42	chr2:23094726..23101497	Plasmid/_
*MdFKBP42a*	MDP0000192242	75	1	370	42.38	5.97	chr2:35303971..35313649	Nuclear/_
*MdFKBP42b*	MDP0000151350	58	1	493	56.18	8.41	chr2:35291342..35296351	Cytosol/_
*MdFKBP42c*	MDP0000195382	62	1	339	38.36	5.28	chr7:1170364..1173209	Cytosol/_
*MdFKBP42d*	MDP0000303422	72	1	197	22.37	5.08	chr7:1174145..1175548	Cytosol/_
*MdFKBP42e*	MDP0000624161	74	1	262	29.54	6.11	chr7:1222808..1224133	Cytosol/_
*MdFKBP42f*	MDP0000129865	67	1	235	27.13	6.38	chr15:2997831..2999339	Cytosol/_
*MdFKBP43*	MDP0000125269	53	1	720	80.33	5.06	chr9:10597121..10600633	Nuclear/_
*MdFKBP53a*	MDP0000238941	46	1	484	53.75	5.11	chr1:24173775..24179861	Nuclear
*MdFKBP53b*	MDP0000927757	68	1	177	19.49	9.78	chr7:22658205..22659109	Cytosol/_
*MdFKBP62a*	MDP0000205111	80	2	572	63.91	5.16	chr10:15327013..15330279	Peroxisome/_
*MdFKBP62b*	MDP0000141863	81	2	572	63.64	5.07	chr5:17589076..17592257	Peroxisome/_
*MdFKBP65a*	MDP0000175388	76	3	457	51.15	5.91	chr15:12488563..12491487	Cytosol/_
*MdFKBP65b*	MDP0000292276	55	3	553	61.54	4.99	chr15:1389508..1392893	Cytosol/_
*MdFKBP65c*	MDP0000202431	71	1	205	22.37	4.72	chr2:23076779..23083272	Nuclear/_
*MdFKBP65d*	MDP0000194793	77	1	165	18.01	4.01	chr2:7447196..7447693	Cytosol/_
*MdFKBP72a*	MDP0000858936	75	3	620	69.52	5.41	chr4:14191542..14195723	Cytosol/_
*MdFKBP72b*	MDP0000146478	71	3	620	69.59	5.35	chr12:22576401..22581917	Cytosol/_
*MdFKBP72c*	MDP0000250482	72	1	191	20.84	6.06	chr4:14188811..14190234	Cytosol/_
*MdFKBPa*	MDP0000182579	No	2	563	63.2	5.08	chr15:1405892..1407392	Nuclear/_
*MdFKBPb*	MDP0000249133	No	1	456	49.93	8.58	chr3:3181572..3190793	Endoplasmic reticulum/_
*MdFKBPc*	MDP0000296958	No	1	1504	166.24	6.16	chr14:28292656..28303065	Mitochondrion/_
*MdTIGa*	MDP0000286595	67	0	537	60.593	5.18	chr11:22521045..22525184	Chloroplast
*MdTIGb*	MDP0000122902	69	0	397	45.77	4.69	chr11:22503161..22506819	Cytosol/_

^a^Gene ID in apple genome (https://www.rosaceae.org/gb/gbrowse/malus_x_domestica/)

^b^The similarity of apple FKBP genes and *Arabidopsis* homologous gene protein sequences

^c^Predicted localization and prediction obtained using WoLF and TargetP

For the nomenclature of the apple FKBP genes, we followed the previously published rules for *Arabidopsis* [34] and rice [36]. Thus, they were named for the FKBP506-binding protein and labeled according to their orthology with reported isoforms in *Arabidopsis*, based on their estimated molecular weights. For some genes in which more than two proteins showed an orthologous sequence with the same gene in Arabidopsis, lower-case extension letters were added to the name according to the order of similarity (Table 1). For example, the putative FKBPs from apple that shared the highest amino acid identity (75, 58, 62, 72, 74, and 67%) with AtFKBP42 were denoted as ‘MdFKBP42a, -42b, -42c, -42d, -42e, and -42f’, despite their respective predicted molecular weights being 42, 56, 38, 22, 29, and 27 kDa. In addition, FKBP15–3, FKBP17–2, and FKBP17–3 were not predicted in apple, while two new members (MdFKBPa and MdFKBPb) were found with the number of their FKBd being 2 and 1, respectively. We next cloned all of the full-length apple FKBP genes based on predicted nucleotide sequences in the apple genome and in the NCBI Nucleotide Database. As shown in Table 1, this revealed that the full-length cDNAs of MdFKBP12, − 15-2, − 16-2, − 16-3, − 17-1, − 18, − 19, − 20-1a, −42a, − 43, −53a, −62a, −65a, −65b, −72a, and MdTIGa had been isolated and confirmed by RT-PCR. Subsequently, the corresponding 5′- and 3’-UTRs of each gene were amplified.

### Multiple sequence alignments and 3-D structures of MdFKBP12, MdFKBP42, MdFKBP62, and MdFKBP72 proteins

**Fig. 1:**
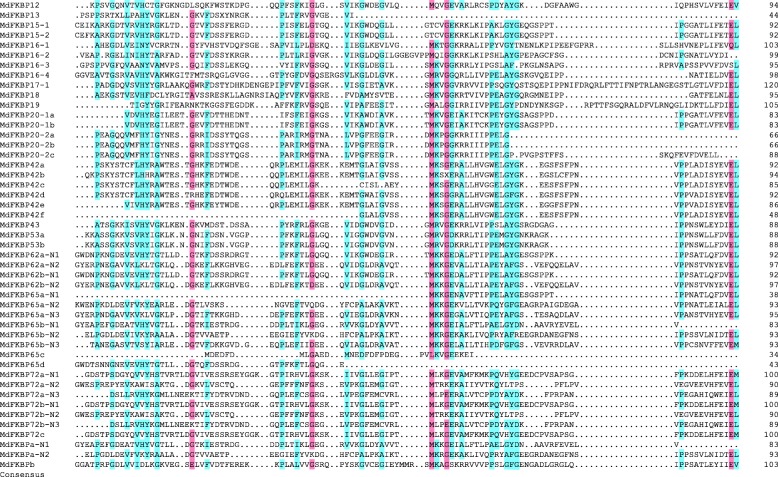
Multiple alignments of FK506-binding domain encoded by 36 apple FKBP genes

**Fig. 2:**
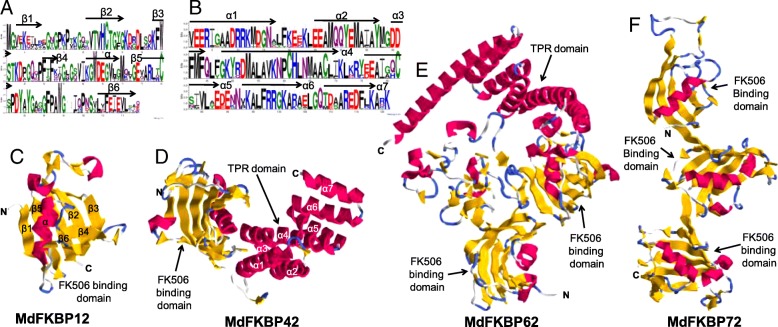
The secondary and three-dimensional structure of FKBP genes. Sequence logos of FK506-binding domains (**a**) and TPR domains (**b**) in 25 FKBP12 and 25 FKBP42 genes, respectively. Heights of symbols within each stack indicate relative frequency of each amino acid at that position. Logos were obtained through multiple alignments of 25 FKBP12 and 25 FKBP42 protein sequences. The a-helix and b-sheet appear at top of corresponding amino acid sequences. Predicted three-dimensional tertiary structural modes of MdFKBP12 (**c**), MdFKBP42 (**d**), MdFKBP62 (**e**), and MdFKBP72 (**f**) proteins (PDB IDs: 5HWB.1.A, 2IF4.1.A, 1KT1.1.A, and 3JYM.1.A, respectively). Yellow, red, and green-blue indicate β-Sheets, α-helices, and strands, respectively. RasTop software was used to generate 3-D representation

Multiple alignments demonstrated that the FK506-binding domains were conserved among the MdFKBP proteins (Fig. 1). To characterize the FK506-binding domain in the MdFKBP12 protein and the TPR domain in MdFKBP42 protein, we produced sequence logos, which enabled us to determine that the FK506 binding domain and the TPR domain from various species were highly conserved at each residue position (Fig. 2a, b; Additional file 6: Table S6). We then used the SWISS-MODEL web server for modeling and analysis of homology among protein structures. From this, we built homology models for MdFKBP12, MdFKBP42, MdFKBP62, and MdFKBP72 protein homology models and evaluated them with homologous templates 5HWB.pdb, 2IF4.pdb, 1KT1.pdb, and 3JYM.pdb, respectively. The results indicated that the MdFKBP12 structure most closely matched that of the fungal FKBP12s [44] (Fig. 2c; PDB ID: 5HWB.1.A; root-mean-square deviation, or RMSD, = 2.05 Å; and 52.43% sequence identity for residues 1–112), the MdFKBP42 structure most closely matched AtFKBP42 [45] (Fig. 2d; PDB ID: 2IF4.1.A; RMSD = 2.85 Å; and 79.01% sequence identity for residues 42–299), the MdFKBP62 structure most closely matched the squirrel monkey FKBP51 [46] (Fig. 2e; PDB ID: 1KT1.1.A; RMSD = 2.80 Å; and 41.03% sequence identity for residues 35–552), and the MdFKBP72 structure most closely matched wheat FKBP73 [47] (Fig. 2f; PDB ID: 3JYM.1.A; RMSD = 2.28 Å; and 32.19% sequence identity for residues 48–384).

### Phylogenetic tree and analysis of conserved domains among apple FKBP genes

**Fig. 3:**
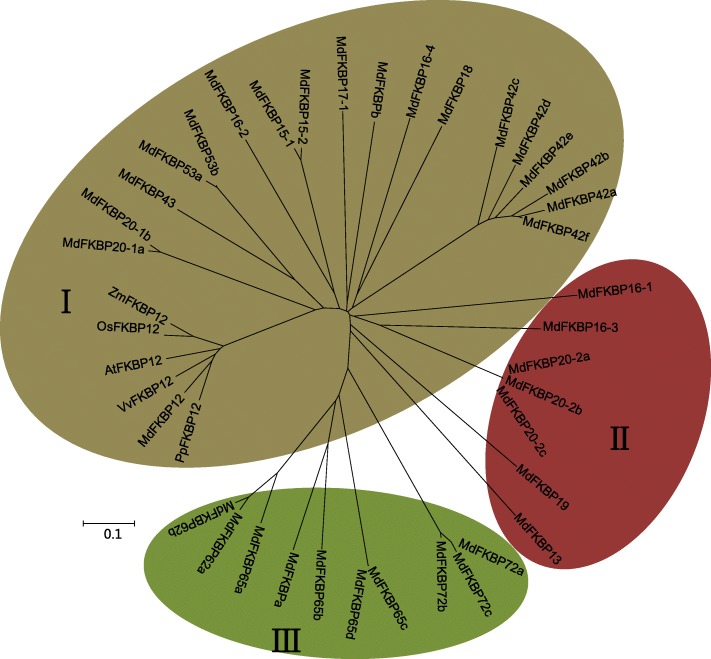
Phylogenetic tree of amino acid sequences from 36 MdFKBPs, ZmFKBP12, OsFKBP12, VvFKBP12, PpFKBP12, and AtFKBP12. Neighbor-Joining phylogenetic tree was constructed with MEGA 5 software, using 41 full-length amino acid sequences from 6 species. Three protein groups on tree are represented by Roman numerals

**Fig. 4:**
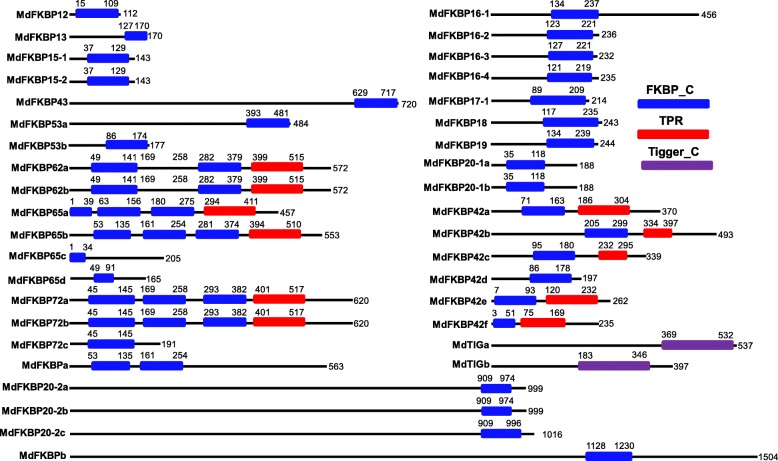
Distribution of functional domains in 38 apple FKBPs. Three conserved domains shown in colored boxes were identified in apple FKBP protein sequences using NCBI batch web Conserved Domains Search tool. Blue box, FKBP_C domain; red box, TPR domain; and purple box, Tigger_C domain. Order in which motifs appears corresponds with their positions in individual protein sequences

To examine the evolutionary relationships among apple FKBPs, the full-length amino acid sequences of 36 apple FKBPs were used, plus ZmFKBP12, OsFKBP12, VvFKBP12, PpFKBP12, and AtFKBP12, to generate a phylogenetic tree. These 41 total FKBPs were assigned to one of three groups (Fig. 3). In Group I, most of the 24 putative FKBPs each had a single FKBd. The exceptions in this group were MdFKBP42a, −b, −c, −e, and -f, each of which contained one FKBd and one TPR domain. The Group II proteins were MdFKBP13, − 16-3, − 19, − 20-2a, − 20-2b, − 20-2c, and -b, each of which contained only a single FKBd. In Group III, MdFKBP62a, −62b, −65a, −65b, −72a, −72b, and -a were multi-domain while MdFKBP65c, −65d, and -72c each contained only one FKBd (Fig. 4).

### Genome distribution of apple FKBP genes

**Fig. 5:**
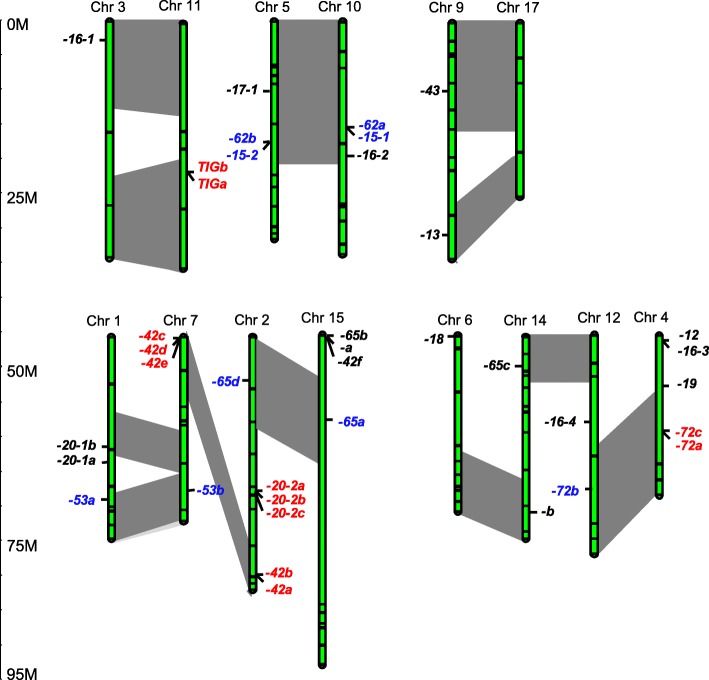
Chromosomal locations of apple FKBP genes. Scale is in megabases (Mb). Two genes could not be localized to specific chromosome. Red font, tandem duplications; blue font, segmental duplications; dark-grey area, genome-wide duplications

The genomic locations of apple FKBP genes were determined by mapping each to genes identified in the apple genome database based on their mapping coordinates. These *MdFKBP*s were unevenly distributed among the 13 chromosomes (Fig. 5), with Chromosome (Chr) 2 having the most, i.e., six, followed by Chr4 (5 genes), and Chr3 and Chr6, each containing only one *MdKFBP*.

This gene family is believed to have expanded during the process of genome-evolution for apple [58]. To uncover the mechanism underlying this expansion, we investigated gene-duplication events, including tandem and segmental duplications, and found that many MdFKBP genes (55.3%, 21/38) were present in two or more copies (Fig. 5). While 12 have undergone tandem duplication, 11 have undergone segment duplication. Whereas those tandem duplications produced FKBP gene clusters or hotspots, the segment duplications produced many homologs of FKBP genes on different chromosomes (see red and blue fonts in Fig. 5). It is thought that a relatively recent (60–65 million years ago) genome-wide duplication event resulted in the transition of nine ancestral chromosomes to 17 chromosomes within the *Pyreae* tribe [38]. Here, our results determined that multiple pairs were each linked to at least 12 potential chromosomal segmental duplications (Fig. 5, pairs of bars in dark-grey areas). These pairs included large sections of Chr2 and Chr7, Chr4 and Chr12, and Chr5 and Chr10.

### Exon-intron structures of FKBP genes in various species

**Fig. 6:**
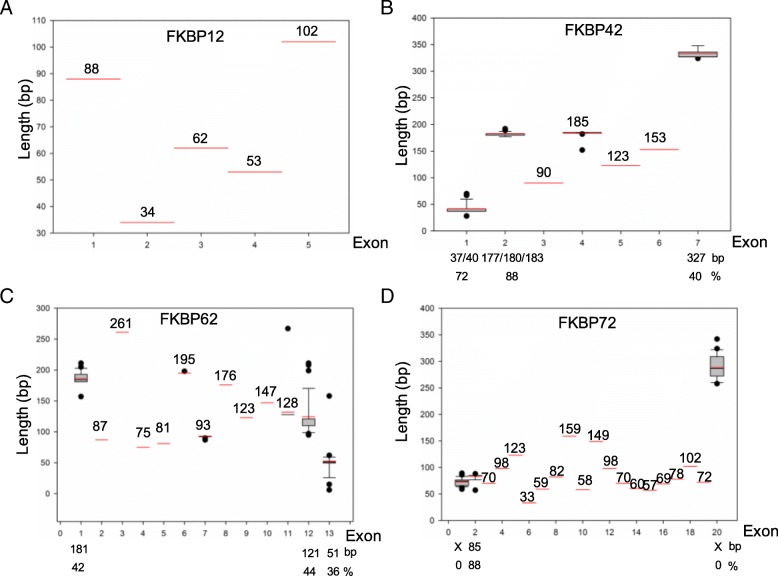
Exon length distribution analysis of FKBP12, FKBP42, FKBP62, and FKBP72 in various plant species. Analysis of exon length distributions for FKBP12 (**a**), FKBP42 (**b**), FKBP62 (**c**), and FKBP72 (**d**), based on Boxplot depictions in SigmaPlot 12.0 program. Each box represents exon size range in which 50% of values for a particular exon are grouped. Mean value is indicated by long red line

To gain insight into their structural diversity, we analyzed the exon-intron organization of coding sequences for FKBP genes from various species. As typical genes, we selected *FKBP12*s, containing one FKBd; *FKBP42*s, with one FKBd and one TPR domain; *FKBP62*s, with two FKBds and one TPR domain; and *FKBP72*s, with three FKBds and one TPR domain (Fig. 4). Among the species tested here, we found that these genes typically contained four (*FKBP12*), six (*FKBP42*), 12 (*FKBP62*), or 19 (*FKBP72*) introns (Fig. 6; Additional file 2: Table S2). We also compared gene lengths and noted that they were restricted in all exons of the *FKBP12* genes (Fig. 6a). The lengths of the FKBP42 genes were most restricted in Exons 3 through 6, while Exons 1, 2, and 7 displayed comparatively more variation (Fig. 6b). For the FKBP62 genes, their lengths were most restricted in Exons 2 through 11, while Exons 1, 12, and 13 showed comparatively less variation (Fig. 6c); Finally, for the FKBP72 genes, their lengths were most restricted in Exons 2 through 19, and Exons 1 and 20 exhibited comparatively higher variation (Fig. 6d).

### Promoter sequences of MdFKBP genes

To investigate putative *cis*-acting elements in their promoter regions, we isolated approximately 1500-bp genomic sequences upstream of the start codon from our *MdFKBP*s (Additional file 1: Table S1). In addition to some types of *cis*-elements involved in light-responsiveness (Additional file 3: Table S3), many types responsive to stresses and correlative hormones were found in those promoter regions. In total, 11 types of *cis*-elements were discovered in the 15 promoters. They were associated with responses to hypoxia, heat, chilling, drought, pathogens, wound, or correlative hormones such as salicylic acid (SA), methyl *jasmonate* (MeJA), abscisic acid (ABA), or ethylene (Table 2). The results concluded that these *cis*-elements play various important roles in plant stress responses.

**Table 2:** The *cis*-acting elements of 15 promoters in apple FKBP genes

Cis-acting elements	ABRE	ARE	CGTCA	ERE	HSE	LTRE	MBS	TCA	TC-rich repeat	W-box	WUN
Response to	ABA	Hypoxia	MeJA	Ethylene	Heat	Chilling	Drought	SA	Defence	Pathogen	Wound
*MdFKBP12*			3/1	1/0		1/1	0/1		1/1		
*MdFKBP15–2*		0/1	1/1		1/0	0/1		0/1		0/1	
*MdFKBP16–2*	0/1	1/0	0/1		0/1	0/2	1/1		2/0		
*MdFKBP16–3*							0/1		1/0		
*MdFKBP17–1*	2/2	2/0	2/1		1/0				2/1	0/1	
*MdFKBP18*	0/1	1/0	0/1		0/1		0/1		1/1		
*MdFKBP19*	0/1		3/0	0/1	0/1	0/1	4/0	0/1			
*MdFKBP20-1a*	2/0	1/0			2/0		0/2	1/0		0/1	
*MdFKBP42a*	0/1	1/0	0/1	1/0	0/2	1/0	0/2		0/1		
*MdFKBP43a*	2/0		1/2	1/0	0/2		1/0	0/1	0/1		0/1
*MdFKBP62a*		0/3	0/2		1/0	2/0	1/0		2/1		
*MdFKBP65a*			1/1				0/1		2/1		
*MdFKBP65b*	4/1		0/1		1/0	0/1			0/1		
*MdFKBP72a*	1/1		1/0			1/0					
*MdTIGa*	3/0	2/0	1/2		0/1		1/0		0/1	0/1	

Sequences and functions for ABRE (ACGT-containing ABA response element), ARE (anaerobic response element), CGTCA (cis-acting regulatory element involved in the MeJA-responsiveness), ERE (ethylene-responsive element), HSE (heat shock element), LTR (cis-acting element involved in low-temperature responsiveness), MBS (MYB binding site involved in drought-inducibility), TCA (salicylic acid response element), TC-rich repeat (cis-acting element involved in defense and stress responsiveness), WUN (wound-responsive element) elements or W-box (elicitation; wounding and pathogen responsiveness. Binds WRKY type transcription factors) were obtained from PlantCARE. (Number of cis-acting elements in plus strand/number of cis-acting elements in minus strand). Blank space indicates no corresponding cis-acting element in either strand of the promoter

### Expression profiles for MdFKBP genes

**Fig. 7:**
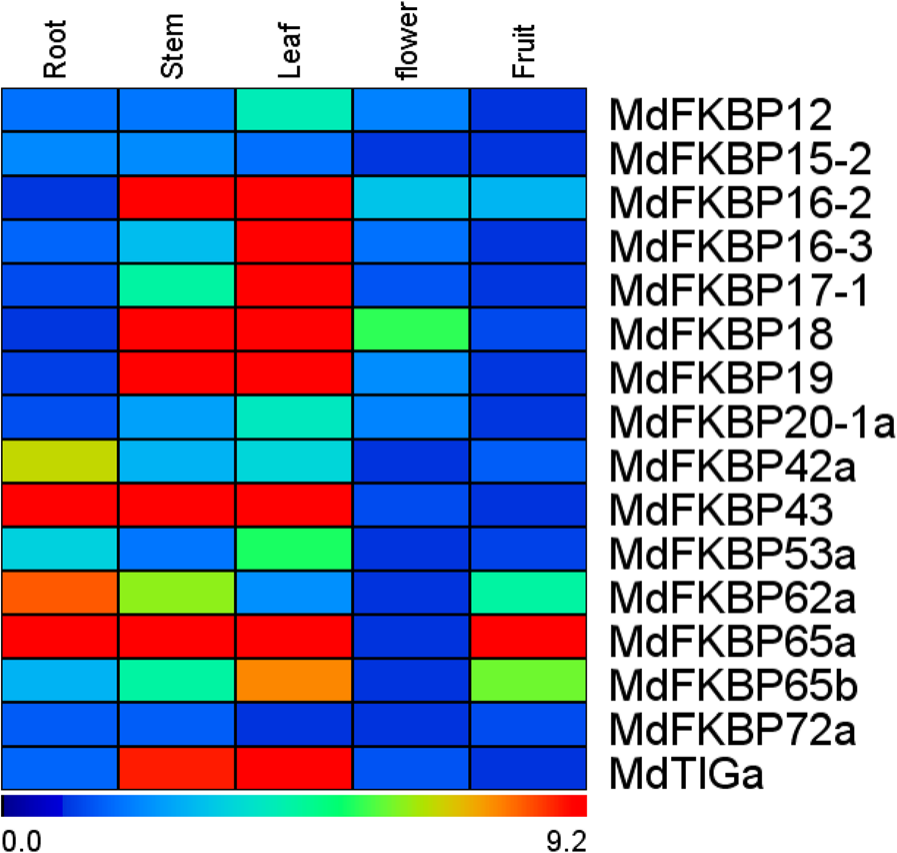
Expression heat map of MdFKBPs in various tissues. Expression values are log2-transformed

**Fig. 8:**
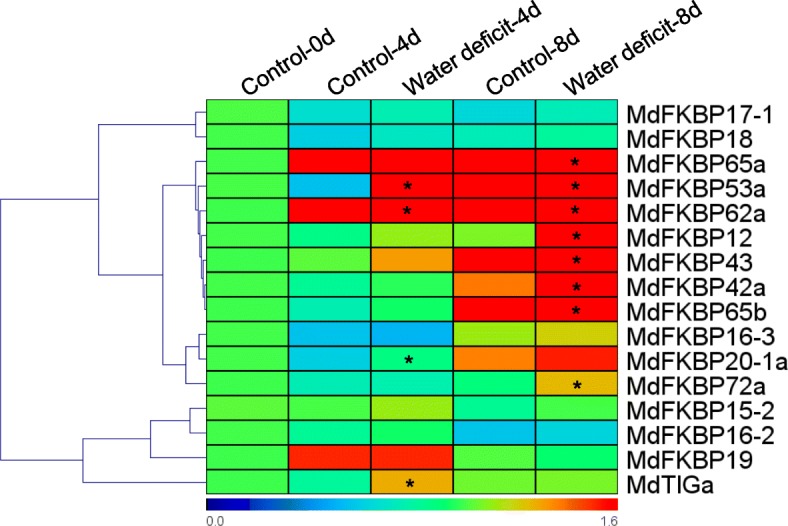
Expression heat map of MdFKBPs under water deficit stress. After qRT-PCR data were re-analyzed, relative expression was calculated with respect to control samples (i.e., Day 0). Heat maps were generated using TIGR MeV v4.8.1 software. Bar at bottom of each heat map presents relative expression values: 0, down-regulated; 1.0, expression unaltered; or 1.4, up-regulated

**Fig. 9:**
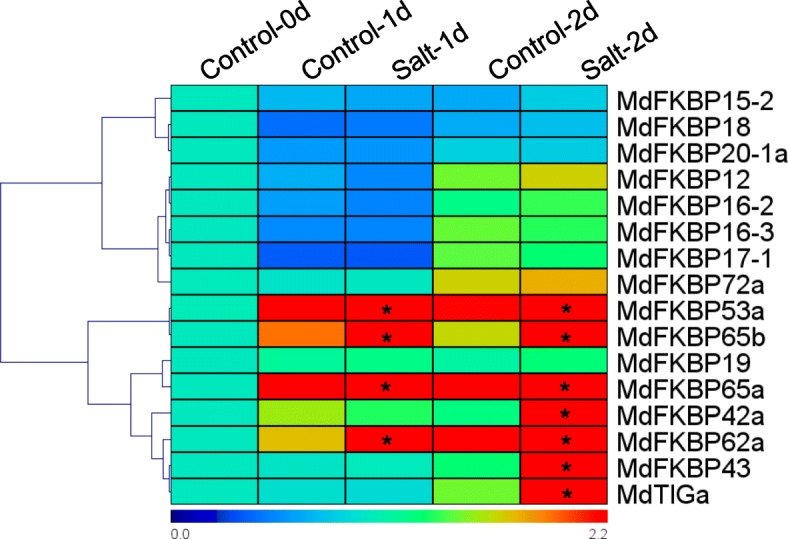
Expression heat map of MdFKBPs under NaCl stress. After qRT-PCR data were re-analyzed, relative expression was calculated with respect to control samples (i.e., Day 0). Heat maps were generated using TIGR MeV v4.8.1 software. Bar at bottom of each heat map presents relative expression values: 0, down-regulated; 1.0, expression unaltered; or 1.4, up-regulated

Knowing the patterns of expression in various tissue types can help us understand gene functions. We isolated the full-length cDNA, 5’-UTR, and 3’-UTR sequences of 16 MdFKBP genes and used specific primers for our qRT-PCR assays (Additional file 4: Table S4). As shown in Fig. 7, *MdFKBP*s were constitutively expressed in the five tissues examined here, albeit at different levels of transcription. For example, *MdFKBP12*, − *16-2*, − *16-3*, − *17-1*, − *18*, − *19*, − *20-1a*, −*53a*, −*65b*, and *MdTIGa* were most highly expressed in the leaves, while expression of *MdFKBP15–2*, −*42a*, − *43*, −*62a*, −*65a*, and *-72a* was highest in the roots. Under water-deficit conditions, expression of *MdFKBP12*, − *20-1a*, −*42a*, − *43*, −*53a*, −*62a*, −*65a*, −*65b*, −*72a*, and *MdTIGa* was significantly induced (*p* < 0.05) from that detected in the control plants while the expression of other MdFKBP genes remained constant (Fig. 8). In response to NaCl stress, transcripts of *MdFKBP42a*, − *43*, −*53a*, −*62a*, −*65a*, −*65b*, and *MdTIGa* mRNAs were significantly accumulated (*p* < 0.05) when compared with the untreated controls (Fig. 9).

### Analyses of MdFKBP62a and MdFKBP65a/b interaction network and co-expression

**Fig. 10:**
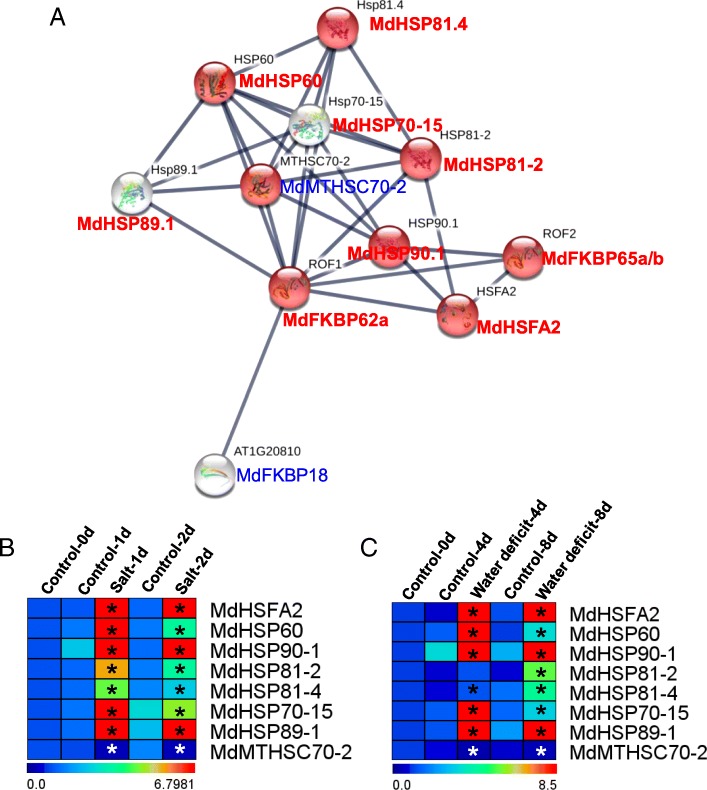
Interaction network and co-expression analyses of FKBP62 and FKBP65 in *Arabidopsis* and apple

Although orthologous ROF1/ROF2 pairs are known to function in abiotic stress responses in some species [1, 2, 7, 27, 59–61], they have not been characterized in apple. Therefore, we chose the FKBP62/65 pair to investigate potential protein–protein interactions. An *Arabidopsis* ROF1/ROF2-mediated network, comprising ROF1, ROF2, AtFKBP18, and eight other interactive proteins, was generated using STRING 10.0, an online database that can, with high confidence, identify interactive proteins. We then determined the homologs of those proteins through reciprocal BlastP analyses of the apple genome (Fig. 10; Additional file 7: Table S7; Additional file 8: Table S8), and used the predicted nucleotide sequences to identify reciprocal 5’- or 3’-UTR expressed sequence tags (ESTs) in the >NCBI EST Database (Additional file 7: Table S7). Our qRT-PCR analysis indicated that, under water-deficit or NaCl treatments, 10 gene pairs (ROF1:MdFKBP62a-ROF2:MdFKBP65a/b; ROF1:MdFKBP62a-HSFA2:MdHSFA2; ROF1:MdFKBP62a-HSP60:MdHSP60; ROF1:MdFKBP62a-HSP90.1:MdHSP90.1; ROF1:MdFKBP62a-HSP81–2:MdHSP81–2; ROF1:MdFKBP62a-HSP81.4:MdHSP81.4; ROF1:MdFKBP62a-HSP70–15:MdHSP70–15; ROF1:MdFKBP62a-HSP89.1:MdHSP89.1; ROF2:MdFKBP65a/b- HSFA2:MdHSFA2; and ROF2:MdFKBP65a/b- HSP90.1:MdHSP90.1) were co-expressed and showed uniform upregulation (Figs. 8, 9, 10). All of these results demonstrated that genes within this apple MdFKBP62a:MdFKBP65a/b-mediated network have potentially important roles in water-deficit and NaCl-stress signaling.

## Discussion

The proteins encoded by FKBP genes comprise large families and are broadly distributed in higher plants [1, 7, 18]. Apple is an economically important woody plant and the most widely cultivated fruit crop in the world. Sequencing of the apple genome has provided a good platform for genome-wide analyses of all putative gene families in apple, including the DREB [62], MYB [63], MADS-box [41], and WRKY [64, 65] families. However, genome-wide information about the FKBP gene family in apple has remained unknown while members of that family have been identified in other plant species [3, 5, 6, 8, 34–37, 66]. Moreover, the content of FKBP genes varies substantially among species. For example, 23, 29, 24, 23, 21, and 23 FKBP genes have been reported for *Arabidopsis* [34], rice [8, 35, 36], maize [37], grape [3], strawberry [5], and peach [6]. Here, we determined that the apple genome contains 38 FKBP genes, making this family much larger than in any other species.

When members are compared among species, it is apparent that some have disappeared or are repeated. For example, in a comparison with AtFKBP members, *FKBP15–2*, *FKBP15–3*, *FKBP17–3*, and *FKBP53* are absent while *FKBPa*, *FKBPb*, and *FKBPc* are new members in grapevine [3]. In peach, *FKBP15–2*, *FKBP15–3*, *FKBP17–3*, and *FKBP43* do not exist but two copies of *FKBP42*, *FKBP53*, and *FKBP62* occur [6]. Similar trends have been noted with rice [36], maize [37], and strawberry [5]. We found two copies of *FKBP15* in apple (versus three copies in *Arabidopsis*), one copy of *FKBP17* (three in *Arabidopsis*), five copies of *FKBP20* (two in *Arabidopsis*), six copies of *FKBP42* (one in *Arabidopsis*), two copies of *FKBP53* (one in *Arabidopsis*), two copies of *FKBP62* (one in *Arabidopsis*), four copies of *FKBP65* (one in *Arabidopsis*), and three copies of *FKBP72* (one in *Arabidopsis*). Based on traditional FKBP nomenclature rules, we named *MdFKBPa* and *MdFKBPb* as new members of that family. We speculated that the presence of additional genes, or the disappearance/repeat of others in the apple genome, reflects the great need for these genes to drive the complicated enzymatic activity associated with these woody perennial plants. These apple FKBPs are highly and structurally conserved, based on analyses of sequence alignments, 3-D structures, phylogenetics, and conserved domains (Figs. 1, 2, 3, 4). Similar results have been reported for *Arabidopsis* [34], rice [36], maize [37], grape [3], strawberry [5], and peach [6]. In addition, FKBP12 shares the same exon-intron structure and the same exon length as found in other species, as well as in FKBP42, FKBP62 and FKBP72 (Fig. 6), again suggesting that genes in that family are highly conserved. Because some members have increased in number, disappeared, or been repeated in some species, we believe this phenomenon means that the content of these FKBP genes has varied during the evolution of this family.

Segmental, tandem, and whole-genome duplications are critical for both the diversification of gene function and the rearrangement and expansion of genomes [41, 62–65]. Whole-genome duplication events have occurred in apple [38], and tandem, segmental, and whole-genome duplications have caused some apple gene families to expand, including the MYB [63], MADS-box [41], and WRKY [64] families. We learned here that 11 MdFKBP genes have undergone segment duplication and 12 have undergone tandem duplication (Fig. 5). In addition, multiple pairs have each been linked to 12 potential chromosomal segmental duplications (Fig. 5). Similar results have been reported for the FKBP gene family in *Arabidopsis* [34] and maize [37].

In various plant species, the expression of FKBP genes is induced by abiotic stresses, e.g., salt, cold, heat, wounding, desiccation, and MDA, including *AtFKBP62* (*ROF1*), *AtFKBP65* (*ROF2*), *wFKBP73*, and *wFKBP77*. Whereas expression of *AtFKBP65* and *wFKBP77* is up-regulated by heat-shock treatment, that of *AtFKBP62* and *wFKBP73* is not [59, 67]. Expression profiles of *OsFKBP* and *ZmFKBP* genes under water stress or different environmental conditions have indicated that many are regulated by water stress (*OsKFBP*s) or by heat, cold, salt, and drought (*ZmFKBP*s) [36, 37]. We found that expression of *MdFKBP42a*, − *43*, −*53a*, −*62a*, −*65a*, −*65b*, and *MdTIGa* was significantly induced in response to water-deficit and NaCl treatments (Figs. 8, 9). In wheat, an orthologous FKBP pair FKBP73 and FKBP77 interact with HSP90 through TPR domains [68]. In *Arabidopsis*, AtFKBP62 and AtFKBP65 work antagonistically and have an important role in the heat-stress response through their interaction with some small heat shock proteins [59–61]. OsFKBP64 and 65, ZmFKBP62a and 62b, FaFKBP62–1 and 62–2, and PpFKBP62a and 62b proteins have TPR regions with conserved residues, suggesting the probability of analogous interaction with HSP90-client complexes [5, 6, 8, 37]. All of These reports indicated that the interaction of this FKBP pair with HSP90 could be a conserved mechanism in higher plants. Moreover, AtFKBP62 directly interacts with phosphatidylinositol phosphate proteins PI(3)P and PI(3,5)P2, which are involved in salt/osmotic stress responses during the germination of *Arabidopsis* seeds [2, 69]. Our data indicated potential protein–protein interactions for MdFKBP62a:MdFKBP65a/b, and co-expression of their genes was uniformly up-regulated (Figs. 8, 9, 10). Therefore, all of these findings suggest that *MdFKBP62a*, *MdFKBP65a/b*, and any gene pairs up-regulated by water-deficit and salt treatments could help confer tolerance to apple plants to those stresses.

## Conclusions

In apple, the genome-wide identification of the FKBP family genes was completed and confirmed 38 *MdFKBP*s. Subsequent analyses based on bioinformatics, RT-PCR, and qRT-PCR approaches were focused on FK506-binding domains, protein and gene structures, conserved domains, phylogenetic relationships, chromosomal locations, *cis*-acting elements, and expression patterns in various tissues or under water-deficit and salt stresses. The interaction-network and co-expression analyses showed that the paired MdFKBP62a/MdFKBP65a/b-mediated network is involved in water-deficit and salt-stress signaling. Those genes were uniformly up-regulated through interactions with apple heat shock proteins. The results of this study provide valuable information about MdFKBP genes in apple and will aid in determining the functions of those family members.

## Additional files

Additional file 1: **Table S1.** Genomic information of MdFKBPs in apple genome. (DOCX 57 kb)

Additional file 2: **Table S2.** Exon length analyses of FKBP12, 42, 62 and 72 in various species. (XLSX 26 kb)

Additional file 3: **Table S3.** Promoter analyses of MdFKBPs. (XLSX 69 kb)

Additional file 4: **Table S4.** Application of primers and sequences. (DOCX 19 kb)

Additional file 5: **Table S5.** FKBP fragments identified from the apple genome. (DOCX 13 kb)

Additional file 6: **Table S6.** The IDs of FKBP12 and FKBP42 in various species. (XLSX 12 kb)

Additional file 7: **Table S7.** MdFKBP62a and MdFKBP65a/b interaction network and co-expression in apple. (XLSX 12 kb)

Additional file 8: **Table S8.** MdFKBP62a and MdFKBP65a/b interaction network in String10.0. (XLSX 29 kb)

## Abbreviations

3D structure: Three-dimensional structure
AA: Amino acid
ABRE: ABA responsive element
ACD: Alpha-crystallin domain
ARE: Anaerobic responsive element
BlastP: Basic local alignment search tool-protein
CRI: Conserved region I
CRII: Conserved region II
DRE: Dehydration-responsive element
ERE: Ethylene responsive element
EST: Expressed sequence tag
FKBPs: FK506-binding proteins
HMM: Hidden markov model
HSE: Heat stress element
LTRE: Low temperature responsive element
MBS: MYB binding site involved in drought-inducibility
MW: Molecular weight
ORF: Open reading frame
PI: Isoelectric point
qRT-PCR: Quantitative real-time polymerase chain reaction
RT-PCR: Reverse transcription polymerase chain reaction
WUN: wound-responsive element
