# Supplementary material for: Genome-wide analyses of genes encoding FK506-binding proteins reveal their involvement in abiotic stress responses in apple
Source: BMC Genomics. 2018 Sep 25;19:707. doi: 10.1186/s12864-018-5097-8 (PMC6156878; doi:10.1186/s12864-018-5097-8)
Supplement: Supplementary file 1 — Table S1. Genomic information of MdFKBPs in apple genome. (DOCX 57 kb) [file 12864_2018_5097_MOESM1_ESM.docx]

>MdFKBP12

ATAAATTGATTCTAATTTTATCGGTCATTTCTTTATTTTCTGAGAAAAAATGCCCCAGTTTTGCTGGAGCATTTTTTTATGTGTTGGGGAATGGGGATCAAGTGACGGCTTTGAGTTCGTCTCACGTCAGGAGGGATCGAAGAAAGAAACAGTCGCCCTCCGAGTGCACGGTAAAATTGTGATTGCCGGTTGAAATCTGTGTTTATGTTGTTCAGTAGCATTTGGTTGCGAAATTGTATTAGTTGTACAGTGCAATGCTTAAAATTTACAATGACGTATCACTACTTCCTTTGAAATGTCGGTTTTCGGAGTGCCGGATTCCGGCTTGTGCACATTTGCACGGAGGCGTTCTTTGCACTTGTTGACTTGACGACTTCAATCGCATTATCGTCGAAATGTAATGGGATCACAATCTAAAAGTACAGCAAACTGGGTAAGAGCATTTTCATCAAACAGGCTGCAGTAAATTTCTGCCTTAGATTGTTATGGCTGTATTGAAGTGTTTTTAAAATGGCTGAAAACGCTTTTGATGAAAATGTTTTTGGAATCTATCTTAAGTAAAATAAAAATGAATCATGGAAATGCATTTGAAGTGCTTTTGAAACTCTAAAACATGATCTTTAAAAGTGTTGTCATTCCACTAGCCTACTTTACACAAATCAAGCGTCTAGTCTCTCTCTCTCTCTCTTCAAGAGTCCGAATATCCAAATCCTTGCCTTCGACACTCTACGTAGACCAATGGAAAAGAAGAGAGAGCCTTCTTTAAGAGTATTTGAATTGAGAGGTTTGAGGTTTTAGGTTTCAGATTTTAGGTTCACTTGTAAGCAAGAATTTTTAGATTGATTATAATTTGAGGTTTTAAGTTCGATTCTAGCCAAAAGCGAATATGAACCACATTATTGCTAACCCGTTGTGAGGTTTAACTCACCTCTTAAAAATTTGAGATGAGATCTACTTGTATTGGTGGGTACCGCCTTTATTAGAGAAATTATTCTAATTATATCTACAAAACATATATTTTATTAGCCACACATTAAGTCAATTATAATTAGATTTGAACTTGCCATTTTTTCTTAAAAAAAAAAAAAGGAAAAAGGTTCCTTTTTTAAAAGGCCGAAAAGACTCACAGAAGTATTTTAAAACCGTAATACAAGAAGCTGTGGAGGGACAAGTTGATTTACATAGTTACAACAAGGCCCACTAAAACAGATAGCAGGCCCAAATGGTCACCGATTAGATTGATTGTAAGATTGATTGGACGTACAAAACCCTAAGCAAAGAGAAGCATCGAGAAGCAGCGAGAAAAGAAGAAAATGGGAGTGGAGAAGGAAGTTGTGAGGGCTGGAAGCGGTGCCAAACCGTCCGTCGGACAGAACGTCACCGTTCACTGCACTGGCTTTGGTTTGTCCCTTCATTCTTTTTCTCTCCTAATTTCCCCACTAATCCAACTAAAAACCATAAAATCAAAATTCTAATTTTATAATTTTATAATTTTTTTTATTTGCTTAATGATTGAATGATTAATTTTACTGTCCTCATCGCAGGAAAAAATGGCGATCTGAGTCAGAAGTTCTGGAGGTAAGCCTTTTATTTTTGTTTTTACTTATTTTTTTAATTTTTCCCTATTTTTTATTATTTATTTTATTTTATTTATTGTTGGTGGAAAGAACAAATGGGTAATCTTCTTTTTTGAGGCTTGTATATATTTTTTTCTTCTAAAAAATTAGTTATTTATATTTTTTGTTTGCGTTTTGTAGCACCAAGGACCCTGGGCAGCAGCCTTTCAGCTTCAAAATAGGCCTGGGATCTGTTATAAAAGGTAACTAACTACGTTTGTGTATATGTGTGTGTGTCTATATATATATATATATATATATATATATATAGGAATTCTTTAAGGGAAGGGATCTTCATTTTTCTATAAAACTGTGGATTAGTTGTGAGGTTCACACCACATCGAACTTTAATGATCTGAACYGTCTATATTTCAAATTGCACCTYATAAATCATCCYTGCAAAATTTTAGCCAAATCAGAAATATTTAAGATATATATTCGGGTTTAAAAAAAATCAAKGTATAATTTRTTATATAAGRAACAATAAAATTTTATTTTAAGAATTAAATAGGCAAATTATTTCAGATTGAATTGAATTTTGACAAAGATRATCTATAAATCGAGACTTACAAAATAGACGGTTCAGATTGTTGAAGTTCGATGTGGAATGGGCCCCRCACCTAATTCTCATTTGTTGAAAAAAAATGGGAATCCCTTTACATAAAGGGCATATATATATATATATATATATATATATATATATATCTATAAATCGAGACTTACAAAATAGACGGTTCARATTGTTGAAGTTCGATGTGGAATGGGCCCCACACCTAATTCTCATTTTTTGAAAAAAAATGGGAATCCCTTTACATAAAGGGCATATATATATATATATATATATATATATATATATATATATATATATATATATATATATGAGGAAGAGGAGGCTTTGTCGTCGGGCTCCTCATAGATGATGTTAGGATCGGCTGGCTCATCCTTATCCGTATTTAGCTACTGATTATGGTACACAATTTTCATTGTTCACACACACGTGGATTGGTGGATTGCTCATGGTTGGTGCGAACTAGATGAGAATCATTCTCAACAGAGTCCATAAGAAGTGTGTTTATATATATATGTGTGTATATATGTGGGTGTATCTATGTATGCATGTTTATTCTTTTCAAGTTGTTTATTTCTTTTTCACTTCCTTTGTAGGATGGGATGAAGGTGTTCTTCAAATGCAAGTGGGAGAAGTTGCTCGTCTGCGGGTATGTTCATCTTCTGTGCTACCAAAAATTTGTGCTCTATGACTTATCAAATAGTTTTGATTTACGTTGAAGTTTCTTGTAAATTGTTATAGTTGAATCGGAGAAGGAACCATTTGGCATGATGTTAATGACTCATTTGGAAAACTTTGAAGTGCCCCGTATGTGTGTCATAATGATCTTTTGCATTTTTTAGCAATAAAATTCTATAAACATGTCATGCTATCATAATTCTTGTTTCATTTATTTCGCTCGTCTAATACTAATAGTCCTGCATTGTTTTGTATATACCTTTTAATACAATGTTAAGTAGTCAAATACTCTATGACCGATGTTTTTAAAGTAAAAGAGTTGATTTCCCTCTTGACCGATTTACCTTTTAAGACACTGAATTTGTGGCTTCATAAAGTTCTTTATATTCCCTGATTGATATGAATTCCTCTTGTAACCGTATGGTCTCAGACCCCCGTGTCATAAATCTGTTGGTTTCCATGGGCGATATGGCATTTCTAAATTAGTGATCTAAGATGTAAGAAGCTAGGAATGTGTTGTGCCCTTATTTTCATTTGGTATGCATTCGTTTAAAAAATCTGGATCTGGAAGGAGATCTAAACATGAACACATCTCACACTCCTTGTTGATGGCTTTGCAGTGCTCTCCAGATTATGCATATGGCAAAGATGGATTTGCTGCATGGGGGATACAACCTCACTCGGTTCTGGTTTTTGAGATTGAAGTCCTGAGTGCGCAGTAACATCCGGGTATGGAGTTGACCATCCCGCATATCCTTATGGTTATACTAAGATATAATTACGAACATCCGTATAACCTTCGTGACCTCTTTCGTTGTTAAAATTTGGCGGATATATCCATATGGTTACCCTACGAATAAATTCTCGTTGTGCCCTGTTATATCAGACATGTTTTAAGGGTTAGTTCTATTGCAATGTTACGAAATGCCCTTTGTTTGGAGCATGGTATGATCATTATGGTAGTTGCTTGGAAGCTGAACCCTGAGTCATTTCTTT

>MdFKBP15-2

GTTCCAAGAGTTCATGTACCTTTGTTATTGTTAATAGCATGTCAATGACAACCAAAGGGATTAATTTGATCTCGAAAGAAAATTAAATCTTAAGTGGAATGCCACTCAATTCAAATTTGAAAGTTAAAAGAAAAGGAAAACTAATGAAAATGGTTTCAAAACTTTAAGTTTTAATGATAAGGACAAAATAAAGGGTAAAGTGAATAGTACCAGGATTGACTTTTTAGTGTAAAAATGATTTTTTGTTAAAGTGAACAGTACCGGGTGCTTTTCGTTAAAGTTCTTTAAAAGAAACTCTAACCGACATATCGCAATTAAAGTTGCTGCCAATTTCATTGGAAATTGGAGATGCTTAATTGGTTGAATAATCTTCAAAAATGGTGGTAGCCCCAATTCCAAAACGGCAAAACCCATGAGAGCAGTTCCATGGGAGCCCTCTTCCCAAGCTATCCACTATGCTATCCACCAAGTAAACAGTAATCGCCCCTAATAAACAGTAATCGTATTTTGTATCTCTACCCAACATTGAATAGTGCTGGCAATTGACAATAAAATATTATTATTATTATTTATAATTAATATTATATTATTTACTTTGTATGAATTAATATTATATTATTAATTTATCTTTTCCTTTTGGTCTTCCCGAAACTTCTACCATTTTTCTTTCTTCTTCCTTCCCACTTCTTTCTTTCAGAAATTTCTTCCTTGCACTGCAACGAGAGAGCACTGCAAATTTATGTTTTACAACTCCAAATTTGATTTCGTTTTTTCCTACAAATTTCTGGAGAAGAGCACTGCAAGGTAATTGACTTTTGGGTTTACCGCAGATTTTGATATTTTGTGTGCTTTCTGTTTCGTTTTTCCAGAATCTGGTGAGCTCCGCGACCAATTCCGACCAAGCTTGACCACGGTTGTTACTTACAGTGGTAGAATCTTGTTCATTTTCATTTTAGCATCATTTTTGTGCTTAAATTATAATTTTTAATAGAGTTTTAAATCGAAATCGGAATCGGGATGAATCCCTGCCAAATAATCCTGCGTTCTTCCTCGAGGGTTCCGGTGACCCATGTTTTAAATCGTGGCTGACGTTTGCGTCAAGTTGCCTTCGGGCTCTCGGGCTGGCAACCCGTGCCAGACCTGTCGCTCTGGCCGGGTTTGTCCCTCGGGCTACCACACGTTAGACCAAATCCGTCGGGCTCTCTGGCCCTGTTCACCCCCTGTCCCCCGAGCAAGAGCTCCCGCTGGAGCTGCTTTGAAAGAAGAGGGGAGCTACACCTGTTTCTCTTGAAAATAATTGTAAAAAGAAAATGGGTTGCAAAGCAAAGTGCAAAATCGACTGCCAAGTAAGCAAAAAAGAATTGTATCGACGTATTGATAACCAATAGAAATCCTGGATTTCAGACCCATTTAAAACCCGAGACCCACCAAAAAAGCACTCGGACACACTCCGACTGAGCGAGGTAGATCGCAAATAATTAGCACCGCTGCTGCTGCAATGAGGGCCGTCGCCATCCTCTTCTTCTTCCTTTTCCTTTCCAGCTCCGGTAGGTCTCAAATCTTTCACCGAATCATATAAATTTCCGATTCCGTTTGGACAAAAATTTCAATTTCCATTTGCTAACTTGTTTGCTTTTCTGAATCTAGTTTTTGGGGACAAGGAGGTCAAAGAGTTGCAGATTGGCGTCAAGGTAAATTACCCTTTTCGTTTTGTGTTTCATTTTTCTGTTTTCCAAGTCCAATTTGTAATCTGAAACGATGTTTGTGTCGAAATTGTGGATGATTTGCTGGAGCTGATTGATTTGTTGTGATTTGTGCGTGGGGCAACTGCTTGGTTGAGTTTAAAGTTTCTTACTTTATGATTCATTGAATTATGATCTTTGCTTTACAAAGCGGTATACTAAACTACTCCAATTTACGGAGATTTGCGCGGCCAAATGGCCTAACTCACATATGCAAGTTATGATCTTTTTGAATAGTATATTTCGTTTAAGACCCTTAGGTTATTTTTTGCGCCCCGTTATTTTCGTTGATCAGCTTGCTTTTTGGATTTGGGCCATTTTCTTTTAAGGGTTGGTTGTTCCTATCATTATGCTATGAAGTGCTTTTGATTTGGCTATGAAGTGCTATCATTATGCTATGAAGTGCTTTTGACTCTTGGGTTTTATTTTTTTAGAAATTGTTGTTATTTCTTTGTTGTTCTCTGGTCGTGTATGGAACTTTGGACGAAGAGGGTTTGCTAGAATTGGTTGGAGGCTTGGAGCTCATCCGTCATTCACCTTTATTGATTATTGATACTTGGAACTTGCTTTTATTTTGCAGTATAAGCCGGAAACTTGTGAATTTAAGGCTCGTAAAGGTGATACAGTCAGGGTACACTATCGGGTAAGTAGAATCTATGGAACAGTTGACAGTGATATGTACGATGGATGTCATGATAGTGTGATGGTATATGCGTGTAAATACTTTCTTGTACTCAAAATAGATTAGCATTTCGTTAATAAATGTGTTACCAAACATAGCTTACGTTTTGTTTTTCTTAACAAAGGTCAAGCTTTGCATTCATATTCTTTTCGATTGCCACATTATTACTTGAGCTGAAAATGAGAGTAATATCTTGTTTTATGAAATTTGAAGCGTTGTTTTAGCAACCTGACAAGGCAATTGTTATTTCATCATTTCTTTGATCTATTCTTATGTAAATTATGCTGCCTATGTATTCGCTACTCCTGGTCTTGTACGTCTTTTTATATGTGGTGAGCTTGTTAGGCCCAAAAAGTTCCTTCAATACGATGTAGGAATTTGAGAGAAACTAATAACCAACTGATATCAGCGAATCCTCCGCAATACCTCTTGAGAATGTTTAGGTTCTGGCAGACTTTGTGTCACATAGTACACTCTGACATTCAACTTTCAGTTTCAGCTCATCAGAAGCTTTTTCTAATTGCTTGCAATCTTTTTCATTCAATATCTAAACTCTACCTGCATTCATGTAGTTGTCTTACCATAAGTTTGATTTTTGAGCCAATATCTCATCCTATAATGAAGACTGAAAAGACTAATAGTTTTTTGTCTCTGACCTTTGCAGGGTAAACTCACTGACGGAACTGTATTTGATTCTAGTTTTGAAAGAGGCGATCCAATCTCTTTTGAGCTTGGGACTGGTCAAGTTATAAAAGGTTTGGGGTTTGCTATTTAATTCGTGTCCATATTCGAATGTAGTTTTCTTACTCTTGTAAAAATACCTTTTGCTGGAATGCATAGCAGGTTATAGGCAAACCCATAGCTTGACAAATGTTCATATAGGAAGCATTTGATCATTCTACATATCTAAGTCTATGCATAAGTAACCAATTTCAGATACTTTACATTCATTAGTTGTTTCTACTGGCTATAAGAGTTTAATGGTTTTTTTCAGCTTGTCAGTATTCTAAGCTGCTTCGTCTAATAATGATGGGTTAATTATGACTATTTTCTAAATTTCACTGAGTGTACTTTTGTATTATATTTGTGGTCTTAATAGCTTGGATTCCTTTCAATTTTTAACCAATGCTGGATTTGTAAGGAATGCTTACTAGATTGCCCTCATTGCATTTTGTTTCTATCCACGTTGTCAATCTTAAGTTTGTCGTCCAAAGATCTACATCATGCAATTTTGGCAAACGTCAAGTGTGATGGGTATGCTGCATGTAACGGCATGGTAGCCTGTTACTAGAACAAGACATATAGTTTAACTGCACTCTTAATATTTTGGTTAAGATCTCCAGAGGTGGGCATATGTGTTGTTACTGAAAACTTAGTAGTATATTAAAGGATTTCTTCTTTGCAGGATGGGACCAAGGATTGTTGGGAACCTGCGTAGGTGAGAAGCGTAAGTTGAAGATACCTGCAAAACTAGGTTATGGGGAGCAAGGTTCTCCACCAACCATCCCAGGTAATAAATTATTCCAATCCCCGCGTGTCCAGACCTCATTTAACGTGAGACATATGTTTAGAAGTACTAATTTATTAGAATATCAATTGAGTGAGGTTGTTATTTGAACGTTGTAGGTGGTGCAACACTGATATTCGAGACCGAGCTGGTGGAAATTCTGGGAAAAAATTCAAAAGCTGAAGATGAAGAGCTGTAGGTGGGCAATGATTTCGTTCTTAGTTTTCTTCAAGAAGTTTCTTGTATCCACAACCCAGTGGAGCCAAAACGTTATTTT

>MdFKBP16-2

TTGGCTACTAGGCAGCGGCCATAGTAGTGGTGGTGCTCCATGGGGCAGGGCTCCTTTTGCCATCGCGTTAAGCCTTGAAGAACATCGGCACATGCCACATCTTCATCTCTATCCTCTGGTTCGCATCTTTCGAAGTAGGGTATTATGTTCGTTGTAGTTATAGGAAACATTCAATGAGACTACGTGTGAAAAATCAAATGTAATAGAAAAATCCGAAAGCAAATTCAAGAGCCTTCTTCGAGTGTGCTAAATCAGGGTCTCAATAAAAACACCAATATGTGGTAGAAAAAATTCGTCGTCTTCAGTCTTGATGAATTTGTACCTATAAAACAATAAACACCTTTGACTAAGGGCCAAAGCCACATGCACCCACGAGGTTGGGAGGGGGGTTGGCTAATGGATCTCCAAAGCCTAATTCAGTATCTCTGAAAAGAGTGAGTGTTTAGGGCTTGTATGCGTAGCAAAATGCTTATCAGAAAATGGTGTAGATGGAGTATTTATAGGGAGGTGGCCGACCATAGTAGGTTTTTACCCTACATGTGGTGGCTTCCCATTGACTAAGGTCTGTCATCCGTAGGATGGATGAGGAGAAAATTATCCCGAAGTATAATTAGGAGATAATATCTTAGAATTATCAAATAATTATCCTAGATTGATTATGATGAGGATTATTTACTTGTTTGAGTTGATCTTCAATTATGAATATATTAAAGATGAAATTTGGTAATTAATTCTATTTTTAATGCTTTTGACTTTCTAAACCATGAGCAAGGAAGCTTGGAGATGTTGTAGTTAACTACTCAAGCCTCTAGGAATATTGAATGCGCTTGGGAATACTTGTGGGTTTGCCCATTTAATGAGGGTGACTTCTTCGGAAAAAAGTTTATGTGTCTTCTGAATTTTTTGGATTATTTTTTACTCCACAACTACATCAAATAACTTTGTAAATATGCCAATCATATATGACCAATATTGACATCGTTCTAGTTGTGCGAGTCACTTTGAACCCGCTCGTATTGTGTATTTATTTTTATGATTCAGCCGTGTACTTTTAACGAGACGGCACTAGAGACCCCTCATAAAGCAAAACTCACTCATTGGAAATGTTCTCTTAATTATATTCACTGAAGTCACTTAGTACTACGTTTAATGATATTTATTTTCACTTGTAAGTGAGAGGTCTTAGATTTGTTTCTCGTCAAAACGAATTTGAACCATATTATTGCTAATTCATTGTGAGACTAAGCTCATCTGTTCTCTTTAATGTAGATAATACCTTTTGTTCAAAAAAAAAAAAAAAAAAAAACTCTGTTCACTGAATAAAAAGGCAAGATGGGGTCTTATCCAAATTTACGGCCAAGATGTTCCTTAATCAGGTCCCATCGTATCCACTCTTCTTTCCCTCTCATGAAAAGAGTCGTTAGCAGTTATTCAGCTCTCAAACCTCCAGACCAAGAACAGATAAGAGTTACAAAATCAAATTAAGATAAATAAAAATGGCGGTTTCTACTCTAATCCTTACAACTCCAAAGCCCCTAGCCCAAATTCAACCCCCTAATCCTCCTCCTGCAACCTCACTCTCCTCCTCCAAACCCACTTCCACTTGTTCTTGKTCATCTTCTTCATTCTCAGCAACTGCAAACAATGCAAACAAATCTTCCTGGAAATTTTCAAATATTTTGAGCAAGAAGTCCGTAATGGATGTGGGTATGGGGCTGTTAGCATCTTCAATTATGGCATTGTCTCCTTTGGAAGCTGACGCCACGAGRATTGAGTACTACGCCACCGTGGGGGAGCCTCTCTGTGACTTGAACTTTGTTCGTTCTGGGCTTGGTTTCTGTGATGTTTCAGTTGGCCCCGGCGTCGAAGCTCCTCGCGGTGAGCTCATCAATGTGAGTTCCCAGTTTCTGCTTTCTTCAGCGCTATGTCAGGTCTTTTATTTCAACAAAAGAGAAAGCATACAAAATTTTTGGTTTTTTATTGTTGTATACTTTCGAATTACAAGTTTTGCTTCTTGTTAATGGGTGTCAAATACAATATGATTACGAATTGCACCCAAGTTCGAATCCCCTTTTCCTCACTGCTTAGGATAATGTAGAATATCGTCTTGTGAAGCACATACATATATATATGTATTTAAAACATTGGGAAAGATAGAAGTGAAGTTGGTTTTATGGCTTACCAAATTAATGGTCTAGTGTGAATCGACCCAAAACAGATAGGTACGCTCTGTTTGGTACAGAATTTACTTCCMAGCATTAGGTTATACTCGCATTTCTTCCCACTTGTGAAGAAGTTGAAACGAAACTTATTCTCCCCAGCCAAATTTGTAATGAACTTATTCTCACCTGCCAGGGAGAAATTCTCTCCATATTACTTGGCAGACACAGGTATTCCAACTCACTTGCACCAAAATACCAAAWATACAAGCAGCATCAGTTTTGGTCTATTCAACTASTTTTGTGATACCGTTTTCTTGGTCTGTCAATTAAATGATTGATTCCAAAAACTCAAAATTKTTAAGAGAGAAATTTGACGATTGTAATAAGGCTCAACGTCAAATGCTCGCCAACATTCTCCCTTGTGTGTAGGTCACTAGGTCTGCAAGAACTAAACAGAACTTGGCAGCACACATTTAGGCAATGGATGACCAAAATTGGCATACCAAGATTTCGATGATTGAATGAATTGATAAACAACCTTATCTTGGTACCGAGTCATGTCTCCTTTTTCTAAAAGTGAAGTTATTACGAGATATGTCTATCAAACACAACTTAATAATACAATAGTCTGTTTTAACGAACACAGATTCACTACACCGCAAGATTCGCTGATGGGACAGTCTTTGACAGTAGCTACAAACGAGGCAGACCTCTAACTTTGCGTATTGGTGTTGGCAAGGTATGAAAATTCTATAGTATCMATTAGATGAGAACATTATCTCTTTKATTTCCTATATGATGAMTTGAAGCTTTTAAATCAAGAATCATACTGCGTAATGAGCATAAAAGCCACAAGAATTAGTGAAAATTCTGTCCGGATAGTCATCTTACACTTACATTTCTGCATGTGCTTTCTATGAATTGCCGATTCTCTGTTCTGAGGCTGTTCCTCTTTTACGTACTTAGATGCATTTTAATCATTCTCTGAAGTCCTAATTAAAGGTGTATTCGATTTTTTCTTTGTTATCATTCTCCCATTGTTGGAATAAACTAATTATTAAGGTTTGCAATGTTTCCGGCCACAATCAACATCAGAAATAAAGAATGTTTCCGGCCACAATCAAACTCATCATGAATGATATTATCAATAGCTGATAAAGGTTTCCTGATGCCTCCATCAATATTGTAGCCYCGTCGAATAAAACTCGGCATGACCTTTTCTAATATCAAAGTAAAAACACTAATTGGCTAGCTTGGATGTTCACTCAATTTCTCCAATTTTAAAGCAGCATCAGAAACAAACTAAACATTTTCTTTTCAGATATCTCTAAATCAAGGTTCTAAAAGATGCTAGGCGCTAGTCGGGCGACAGGCTGGTGCCTATCCCCTAGGCGGCTAGGCGRGGCCTAGGCGGACTAGACGGATTCAAGAAATTTATGATATAYTGTATAAATAAGTGTCKTTTTATGCTTAAAAAATAACACACATAATTATATTGGGATACCTAAATTGCARAATAAAATGACGCATAAATTATTAAGTATTAGAACATCTTGAAAACATGAGAAATAAACATATACTGAGTGTTTTCCAATTTCCACATGGATGTGTTGCAACGGGTTTCAAAATACTTCAGAATTTTATCACATTCTTCACCTCTTGTATTAATCAGCATTGCACTTTACTATGGCAATATCCATATATTGTATKTATTATTCATAAGTTTTTCAAGCTCGTCATGGTGGGGTTGAATAWATAGGAGAGGAAGAGTCTTGCTTAGCTTCTTGTTTGAGGTTTTCATCCTAAATTATTAGCAATAATACTAAATGGAGAATTAACCCCTAATTCCAAGTCCAGAGATTAAGAATTAAACAAAAACAATTTTCGTGTTGCATTTGTCAAAATCAAAGCAACCAAGGGCTCTAAGAAACCAGAGTAGAATAAAGAAACTAAACGAAGTACATCAAATTGTGGACYTGAGAAATGCTTAGTTTTCCTTTTCTTTTGATTCTCTCGGTGTAGTCTCCGCCTCCATACCTTCATTTATTCATGCCCATTTACCTTTATCTTATATTCTGGTGTCCAGGTTATTAGGGGACTGGATCAAGGTATTTTAGGGGGTGAGGGTGTGCCTCCAATGCAAATAGGTAAGACGATAAAGTTGGTTATGTTTCTAAATGTTTCTTGCCATTTGTGAAAAAAAAAAAAAAAAAAAAAARAAATCAAATCGTAACCATCAAATATCACACAGGGGGAAAGCGGAAGCTTAAAATTCCTTCACATTTAGCATATGGGCCAGAACCTGCAGGATGCTTCTCAGGTGAGTTTCCTTTATATTTGAAACTATCTTGATTCTTTAGTCTTCCATGATTATGGTTTACGACTGTTGTCGTCAACCAAAATCATCATCAGAAACTTGAAACTTTCTATGATCTTACAAAAGACATCATTGAATCACTATCATCCTCACTATCTGTGTACGTGTGGAAATACTTGGTATATGCGATCAGTTAACTTTGCAGGACAAAACTCTTGTCGATATCTGCTACCGTAATTTCTTAGAAAAGTAAAAACTGGATGTCAAGTACAGTTACTTATATCGATGGTATAATGATGATGTATGCTACAGTTTCTCAAAATCTGAGGCTTGCTAATTTATTCCTAGACCTTAAGGTAAAAACTAAAATATTTTAAACATACAGTCAGTCGTTTTGAGTCTTGGAAACTATCAGAAACTAACATATATGCATCTTTTATAGGTGACTGCAATATACCTGGAAATGCTACTCTTGTCTACGATATTAATTTTGTTGGCATCTACTCGGGAAATAGAGCATTGCCGGGCAAATAGATAGAACACGCCATTAGATCCTTGCATCTCCCGACTTCCTGATACTAAGCTTCCATTGTTAAATACAAGCCGAGGCAGGAATTCAGAGTCATACATATAATGGTGCGCCTTCTTTTTCTACGCTTGGCTTGCTAAATGGAGATGTATCGATCCCATCCGCGTTGGTATCGCCCCACCTATTTCATTCTGCATTCTACCTTACATTTTATGTATATAGGTTTACCTTTGTGGAACATGGGACGATTCGTATTAATGAACATACTTATCTTATGTGATCGAAACACTTGGAAATAATGGCTTGTACCTAATTTGCGTATAACTACAAAAGTAAGTTTTATAAAAGATGTTCAGCCAAATCTTTTGGATCGGTTTACTA

>MdFKBP16-3

GGGTGTTTGCGGAGAGCAGAAGGGGGGAAAATAGCCTTTTAATTCGGCAATCTTGGCGGAAGCGGATGCCATTCGTGCAACACTTGTGTTTTGTAAAAACGAAGGATTTACGAAGGTGGAGATTGAATCGGATGCCCAGGTGCTATTGCGAATGATTAATAAGGAGATTGAAGTGGATGCTGATTTGGAATATCTTCTGTTTGATATTGACTCTTTTTGGTGAATCATATTGGAGATGTCAAAATAAGGTTTGTGCTGCGGGGAAGCAACCTGGCTGCTCACACGGCTGCCTCGTTTATTGCCAAACATGGTGGTCCATTTGTTTGGGACGAGATAGGCCCTAAAATTTTATTTAATATTCTTACTATGGATGTAAACATTTCAATTAGATTAAAATTAAAGTCCCTGTTTTGACAAAAAAAAAAAAAAAAAAAAAAACTTTTTCTTTTTTAAAAGCAAAAAATTAGAAGAAAATGAAAAGCCCAAAACAGAGAACGGGCCTCCCATTTTTCGATCAAGTTCCCTGAAGATTCTGAAAGAGGGAGAGAGTGCACAAGATGAGGTTTAACTGGCGGCATAGATGAGCGGTTGTCGGCGGCGGAGGTAGGCTGGGGGGGCTTTGGTGTTTTCCGGTTGTAGTGTCTTTTTCTAAGTTTTTTATTTTTTTATAGAGTTAACGGAGTTAAGTTGGTGTGAAATGCAGGCAGAGGGTGGGCAGAGGAAACCTTGGCACACCAGATCTGCGCCCAGGCTGGACCGACCATATTTATACAAACAACAAGATTTCGTTATCCGTTTGTGGACGTTACAAACTCTTCACTTCCAGCATGGCTTCGTCTTCCTCCACTCTGCTCTTTCCATTTGGTAATCACTTTTGTTCTTCATTTTTCTTATTAATTTATTTATTTACAATCCATGCATTACTTTACCATCTTCGTAGTTCTTATCAATCTCAGTGTGATTCATTACAAACCCTAAATTCGTAACTGTAACATTTNCCCCTCTGTTTGGAGGGAGAGACGAAAATGTTGAGACCAATCGAGAATGTAGTGAATTATCCATTCCAATTCAATCNTCGAGAGCAAACGAGGCCTAAAAATTGATACATCAAAAGGTGTTTAAACGGCTTGTGATCCAATCCAAGAAGCTTTGTGCTTGCTCGTGTGTNTGTATGCCGCGGCTTTAGATATGCTGTTTCTCTTTTTAAGTTCTACCGAAATGCAATATCACAGTTTTAGNTCTGTTGCTTTTCCTACAAGTTAGGTTCATCTTCTGGAAAAAGGATGTCCGCAGATTACCAAGGCGTTTCCTGCGACAGGGTTCAAGGGATGCTNATCCGATGTTCTAATTCACAAGTTACTGTAAATGCTACAAGGTCAAGAGTATGTAAGGATGCNTTTAANGTGATCAAAAGAAGAGATATGATAGGGTTGCTTTTCGGAGTTTCAAGCGCTGTGACAGGCGCATTTGAAGCTGAGGGAGCTGGTCTGCCCCCAGAAGAGAAGCCTCGACTGTGTGATAATTCTTGCGAGAAGGAGCTTGAAAATGTATGGTGAGGTTGAATTGATCCCTTGGCTGCATTCTATATGTTATGGGAGTTCAGCTGTTAATTATTCACCTCAAAACTCTAGCAGGCTGTAAAACTTGGATTTTACTGATGTCTCGATTTATTCTCTTAAGTCCTTGAAATTAATATAAAGTTTAGGAAATAATATCTTGATTAAACTCTTAAGTCTTTAAAAATTAGCTCAAACTGGAATGAACGAAGTTACGTTTTAGGGGAAAGTTTTTTTCTGTAGCTCGCCCAAAGAAAAAGTTCTACTTAGGTCTTTTAGAATCAGCTATAAATGATATTTTCCAGGCATTAACGTTTAGGTTCAAAATAGATAGGGATTATCTCAAAGTTATTAACCAATCCAAGGTTATAAATCTAAGTTCTAAATTATGATTCTGCACCCTAAAACAATCAACCATGTCAATAAAATAGATACCAAGTTAAAGAAAATTGAGTAGAAGTGAGGTCTTCACAACGCTTCCCNCAGGAGCCATCTTAAGCTTGAGAAATACTTGCACAACTTTATAACAGCTCATATGAATGCACACTGTGGCCTTCATTTGGTAAGGACTGGATTGTTTTTTATTAAAGTGGTNATACCGATTCTTTTCTTTTGCGAGTTCAGTGGGTGTCTGTCAGTGGCAAATGCCTTTTGTTGATATGATCTATCAATTGTACTGGTAAATGATGTTTGAAATTGTCACTTTGTTGAAGGTGCCTATGGTAACTACAGAGTCTGGTTTGCAGTACAAGGATATTAAAGTTGGTCAAGGCCCCAGTCCACCAGTCGGTTTTCAGGTGAGTAAGATTTAAATCATCCTGGCTTGTGAGAACTAGATTTCACTAAAAAAAAAGGTCGTACCCAGTGCACAAGGCTCCCGCTTTACGCAGGGTCTGGGAGAGGTGAATGTCGGCTAGCCTTACCCTACAATCTAGAAAAAAAATGGGATTTTCTGAAAGCGTTTGATGTTTGTGGGTTTCTTGATTATGTTGATTGATTGATTGATTGATTCTCATACTTGGTTTGTGTTATGAGCATGCGCTTGATTATTAAAAAAATTGAATGAAATTTTCTTATCTGATATTGTCTCTGGCCCCCTCATCTCGGCTCTTTTGTGATTCCCTCTTTCTTACCAATAGACTTCTTACAGTCCTTTATAGAGTCACTGGTGGTTATTTCTTCTGAAGCCATAGCAGAGAGGATTGTGCATACTTAAATCTACCCTGTCGATTTATGGACATTTGTTTTTTGTGTTTCTGACTCTTATTACATTGGCGCTGTTTGCATAATTTCTGTATTAACAGGTAGCAGCGAATTATGTAGCCATGGTTCCATCTGGACAAATATTTGACAGGTAANCATATTGTTATATCTTNATAGAAGTGGAAAATAGAAATGAAATGGCTTAATGTAGATCAGNTGATCTTTTGGTAAGAAACATAAGTTTATGAAAAATAAAATATAGCTGCGGACAAGTTGTCCCTCTAAAGATCTGAAATTCTGAATCAACAAAGAGAACGTTGGAACCCTCGTTTCTATTTGTCATTATNGTCCTCTCTTTCAGGTTTGAGAGTGGCAGTTTGTGCCTTTGACCNGTAATGAGGAGCTACTCTCNCCTAATCCTCTATTTATNTTTTTGTTTGAAAATTTTGGACACCAAATTCTGAGTTTGTCCCATGAAACCTCCAGCCCATNTCCCCCACATCCGNGTTTATACAGACTACTACTCCATTACCTTTTGCCTTCTTACCATCACTAAACTTATTGTGCTTCTCANGAAAAAGGGTTATGCATGTAGTTGGGTTTACCCAACTCAATCAACTTCCCTGAACAGACTCAACCACCAGTATAATGCTATTGGACAGTGAAGAAACAAATGATCCGCATTTTCAATCTCTACATATGCACCATTGGNGAGAAAGACAAAACTGTGGGTCTTCTCCTTCTGATCAAATCACAGAGGACTTACGGTTCAGGGTTTCAGGGTGCTGATTACTGGTTGTATCGTGCGTAGACTAACTAGTGTAAAACCACAGTGCATAACTTTTGCTTGCTGCTTAATGATATTTATAAAAGGCTACTATTATCCAACAATAAGGTATCTGAGAGAGATAAAATTCATATTGGTAATGTATAACTGTATAGTCTTGTTATTTNCTAAAGGTTTGCTGNGCCGGGCTTAATTTGTGGAGCCGTCCTACATTTTTATCTTTTTTTCTGTGTAACGTGTCTTGTACTTGACAAGTTTTGATGGTGGGTAGGACTAGACTCAGTTTCTTACTCTTTTACTCTTTCTTCATTTGTACAGTTCATTGGAGAAGGGTCAAGTTTATATATTTCGTGTTGGCTCTGGTCAGGTAAGCTCTACAAATGTCTTCAAGNATTTATTTTTTATTCATTCTATTTTGTTTCCATCACCCCACCTCGTCACTTGAGAAGAGTAATTCGGGTTATGATATTGTGGTCTGTTTGTAGGTAGATTTCAAATTCAGTCTACTTAACATTGAGTCTTCTTGAAACAATTAACNCACCCCCAAACACGACACATTTGGTTTCCCAGTAATCAATGGAATAAGGGGTATTGATGTTGTTCTGGGGATAGCATGTTATCATACAATAACGCAAGCAAAAAAATNAATAAAAAGAAGAAACCCTAAAGAATCAAANCATAGATGACTAAAGTGATGTCTCAAGGGTTCTTACTGCATCATTTGAAGTGCTTNATCACATTTTCAGTTTGTTATAGGAAATTCCNAAGCGGAACAACTTTCNTAGGGGAAATGAATGTTTGCTAATATTTGTAATTCAAATGAGTGGAACAGGTGATCAAGGGACTTGATGAAGGGATCTTGTCCATGAAAGTAGGNGGGAAGCGACGACTCTACATCCCAGGATCGGTAAGGCTTTTACTTGGCTTTCTTTGAAAACTAANAAGGAAAAAGAAAGGTCTCGAAAAAAACCTAGGACCTTATTCCAAGCATTGTGCATTTGGAGTGCTTTANAGTTGAACTGTTGAAGTGCTTATTCTAAGAAATCACAGCAGGAGGGTTCTAACCTTTGTGCCTTTTTCCTCTGTTTTTTATTAGCTGGCGTTTCCCAAGGGTCTCAATTCAGCTCCAGGGAGACCAAGGGTGGCTCCAAGCAGTCCGGTTGTTTTCGACGTGAGCTTGGAATACGTACCAGGCCTTGACATTGAGGAAGAGTAAGAGAACAGATGCCTCTCTTCTTTTATATATTTTTTTCTTTGTTAGTTTTTTATCTTTTCTGAACACAGGAGCAACAAAAAAGATCCATTAATTCAATTTTTTCTTCATATTTTTTTGCAAAAAAATATTAAATTGTCTTTCCTTAGATTGTTTCGTGAGATTTTAGAACATTATGC

>MdFKBP17-1

ACATCCAACCAAAAGTATGCATGAGTTCGTAGGGTTACAAGCAAGGTGATGATGGCGTTTGTAACCTCGATCTGTGCCTAAAAGGATAGTTTCACGAGTTGACCGTATAAGTTGTACGGACAATGGAGGAAATCCGAGAGAGAAGAGAGAGAGAGAGAGAGTCAATGGAAGAGTGAGGGTGTGTGTGTGGTCCAGTGTGGACTACCAAACAAAAACAACATAAACCTAAGTTCTAATTGGGTCCAAATACTTAGGAAGAAAATGGATTGGTCCAAATCCATAGCCCATTTACACACACACAACTCAACACTCAAGGGTATAATCGTCATTCCACGCAATCAAAGATAAAAATAGATTATTTTTCGAGACGAGCTGTCACAATAATTATTATTAATTTTATTAATTTTGCACTCAAATAAAAAAATATTATTCATAAGATTTGGAGGGTTTTAAAAATCTAAACGACCATCTAACCATCCTCTAAGCATAAAAGATGCATTCACGAGCATTTATATATGATTTCACATTTTTCAGACTTGTTAATGATTATGAATTTTATTTATTTTGAACTTCCTTCAAAATACTATTCATGACGACATGTATGTTTTTATTTCTAATGTGCGTTATAATTTTTTAATCTCATTCTTTGCCTATAGTATACTTATAAAAATAAATAAATAAATAAAACTTAAATTTTTTAATTTGTATAGGATAATAATACAATAATATATATTAAATATACAATACATACATATACACTATAACTATTGTATTTTATGGTAAGTTTTTTTAGTAGTTTGAAAAATTAAAATCATCAAAATAACTTTTTTCTTAACGGGCTACGTGCGTGTCACCCTTGTGTAAACCTGCTCAAGTCCTGTCATTAACAGGTTCGTATCGTATTGACTCAAAATCTATTATTTTCGTGTCATTTACGTGTTGTGTAAATGAGTTTTGTAAGAAATTGACAGGTATAATAATATGAAGAATGGATGATTTTCTTAATATTTTATGTTTCCTCTTATTAATTTAGCAATTCAATTTGAACTCTCAAGTTCCTAGTTGATTTGTTCCTATTCTAACCAAGAATTTGGTTGACGAAACTTTAAAACGGGCCCGGCCTAGTCCCTTTAAATTTGGATCCGACCCGGCTGTGCCCCGTAGTTACCATTAAAGTTGGTTATAAATAAATCGCTATATTAAAAGAAAAATTATATATAAATTGCCACTTAGTATTACGATTTAGTGATATTTCTCTTCATTTGTAAGAGATCTTAGGTTCAAGTGGCACGAAATGCGAATTTAAACCACTCTATTACTAATCTATTATGAGACTAAGTTCACTTCAATATTCCTTGGTATAGATAATACCATTTATTAAAAATAAAATAAAAAAGGGCTGAGGGCATAAATGTATTTGAAAAATACCAACACTTTGCAAACAAAACCATAAAATAAAGAGGATCCCCATACCATACATCTGAAATCTGAACAAGAAATATGATTTACACGTGCTTCGCGCCACTACAATGCTTCCATGTTCCCCACCATCCAACCGTTCGTTTAGTTCATCCCAACGTCACCTCTCCAACCCCATCATCGTTAATCACAACTAGAAGGGGAGCATTATCCACTACTTTAATCTCCACCACATTATTCACGCTGACACCTCCTTCAAAGTCCGCCACCATACCCGAATTCTCAGAGCTTCCAAACTCCGGCGGGGTCAAGGCTCTGGACCTTCGCCTTGGTGATGGTCAAGTACCCGCCGATGGCGACCAGGTTATTTTTCTCTCCCTTATCCATAATATGTGCAGGCGTGAATGTGTGTATACTTATAAGGTTATAACACTGAGATAAGGGGATGTTGCGTATGTGGCTGTATGGCAATGACAATCTGCACATTTTGTCTTTAAATATTTCTTATTTGGGCTATAATCGTAGCAATATTTCCTTATACCACGAATTTATTTTTAGTCAATGTAGTCTAATAATAGTTGCTCTTGTTCTCCAATTATGTTCTGTGCTGCACTTGTGGTCCTTTTCACAAGTTTTGGATTGGATACTAGGATAATCTGAAGGCCTTTAGCAGTGATTGGAACGGAATGTGCATATCCAATTATCCATCTAAATGTTTACACTTTGATTGTGAAGGTTTCTGTTCATTACTATGGAAGATTGGCAGCAAAACAAGGATGGCGATTTGATTCGACATATGATCATAAAGATGAAAATGGTGAACCAATTCCTTTTGTGTTCGTGATTGGGTCCGGCAAAGTAAGTCCTCGTCATGTCAATATGATATCCTTGTGTTAGTATTACTACCACCTTTGGATTATATTTATCTGTCAGATTAATCTCCACTGTGATCTTAACAATTAATAGAGTTGTTTCAGTGCTGCAGCTTATATTCATTCATGTGATTTGATATTTCATATAAGATTAAAATAAGTAGGTCTCTTGAATTTGGGAAATCCTGTAAATCAGGAAGCCCGAAGGGAAGTTTCGTTGACATATTCTATTTTGTTAGTAGTTGTTATCGGTCTACATCTATTGGATATTGTACTGTGCTTCAGTTTCTACTACAGACTTGTGCAGATAAATGTGTTGATCTTTGACAAGCTTGTTCAGGTCATTTCAGGGATTGAAACAGCAGTTAAATCTATGAAAGTGGGTGGTGTTCGTCGGGTTGTTATACCGCCATCCCAAGGGTATCAGAGCACGTCACAAGAACCTATACCACCTAATGTAATTCCTCTGCATCTTCAAGATTATTTCTTTTAGTCTGTTCTCTTGGTAANTTTATTCTTAAACAGATGTATACCCATCTTAATCGAACAAAATAGAGACTACTTACGCCACTCAGAAGACCTTTTGCTTGTTTGTTTTAATAGTCTGCTATCTCTGTGTGTGTGTGATATACTTGACACATCATATATGTCATACATGTAACTTTGAATGAGTAGATGGATAGATAAACAACGACTTGCCTACTTTGTACTGCGATTAAAATCCAACGATTCCAACCTAATTCCACCCTTGTTACTCAACTCTGAGGGTTTGTTTTCTTTGTCTTTCCTCAGATTTTTGACAGACAGAGACTGTTTACAACCATTTTTAATCCAACTCGTCTTGCTAATGGAGAAGGATCAACATTGGGGACTCTCGTGTTCGATATTGAGTTAGTCAGCCTGAGGCATCAATGAGATCACCTTTTATCCCGAGCAGTACTAATAGAATTTTACGGCTTAATTCTCATGTATTGCATAATTTGATATATAAGTACATACAGTCCTTGTTCTCCAAGGAAACAAAAAAGGACACAAAGAAATATCCTTCCTAATAAAGGACTCTAATATTTACACAATATTTACATTCCTATTTCCCTAAAGATTACTCATGCCAATACTCCCTCTCAAGTTGGAGCATAAATGTCACACATACCCAACTTGATAAGTGAGTTACCAAAAACCTACACACGGCATAAGTGAGAATATCTGCAAGTTGTTCTTCCGAGTTCACAAACAGTAGTG

>MdFKBP18

TTTTCGCGATTGTGGCGGTGAATGTGGGAGAAATAAAACATTGAAAAAGTTGAGGCTCCGGCAAGCAACTTAAGGCCGGCAAGAATACCAAATATCCCTATTGTTTATGAGTGACTGTGCATATGATTTGTCACATCATGACATCTTGCAAATTCTCCAAAGTTAACATGAACTTAATCTTGTACAAGTAGCAGGCCAGAACAATCCATCAGCTCAAGACTTACGTGGTGAAAAGATCCCCTTCTCTTTCTATGTAATATTATTTCTTCATTCAATGTTTTGTTTTAAAACCAAAAGTATCAATCAACTACATTGATTTCGTGTTCTTTAAATTTTAGAGTTATTAACACAAACACCTGCAATTTGGCATGTTCTATTTTAGATCTTGTACTTTAAGAATTTACACGAATGATCCCTAATAAAGTTTTAAATATAGTAATTTAAATTGTCCGTTAGCTAGTGTTCTATGAATCATTTTCTAACATCAAGTTTAAAGAAAAAATAAACGGTCACTCACATGTCACTTTACATTGGGCAAAAAATACATTAAGAGTAGAGGATAATGTGAGACTCGCAAACTTGACTTAAGGGGGTTTAAAGTTAGAGTAATTTTCAGGAGATAAAAGTCAGATTCATACCATCATTTAAAAGATAACAAATGTTGTAAGAATGGACGTCATATGAGTCTTCACTATTCACTGAAGTCTTTTTGCACATGATGAGATATTTTTCACTATTTTACTCCAACAAGTTTGGTGTATGGTTGTGTAAGTTTGTTTATGTTTTGTTAGTGAACTTTTCTATATGAAGGGGTAACTTTGAGACCCCAAACACGATCGAAAAGCCAACTCTACTTTTTCCTTTGTGAGCACAAAGCGCAAACAATAAATCATATAAGTTTTTTTCATTTTTTTCCTTTGTATCCATCTTCCATATTCAAAACAAGTAAAAATAGTAAGACGATGTGATATGATCTTGTTCCACTCCAACCCCAACAAAGGTTCTCTTTCGCTTTTGCCTATTTAATAAAACAAAATATTTCATCCTATATTCTTTTGCCAAATAAAGAAAGAGTGTAGTATGGGTATTATGGTAAATTGAAGAAAAATAGGATATGGATATGGTATAGAAAGGGCGGTTGAAAAAACAGGCATCTGCTGCTGTATTTCCAACGGCCATGGCATCAGTAAGGTGCTTGACCGTTAATCAGCGTCTCAGTCGTCATCANTCTCATTCAAAAGGTGGCAGCGGCGGCAGCGGCAGCAGTACTAAAGGGCAAGTGGAACAAGTTTCAGTAGTACAAAAAGTGCTGCCCATTTCCTCGTCATTGATTTCTTGTTCAAGAAGATGTGCTGCCATTCTCATTAGTACTTGGCTGCCTATCACCCTCATTTCAGCCGCGTCTCCTCCTCCGCCTTCACTGGCGAGAGAGCGACGCAACAGGAAAATCATCCCCCTTGAAGACTACCGCACAACCCGTAAGTTACTTAGTTACTCACTTTCTCATATCTAACTGTTTCAATTTCTTAAATTTTGTTGTTGATTTTGAAGGATAATTATGTACGAAGTTGTATTTATCCATTGGTAAAATTGGTTGTATATATTTAGGTGCAATGGTNAACCCACAATTTATTCCGCACTATGAAAATTGCAAACTGAAAACAGTTACCAAACAAGTTTAACCCATCTTTGCTTGCTTGATTTCATGTTCCTTCAGCAGCTGATGGATTAAAATATTATGATTTGGTTGAGGGAAGGGGTCCCGTAGCTGAGAAAGGATCAACAGTTGAGGTACATACATGCATTTGTGTTTCTAATTTAAGTAGATTGATTGATGTTAATTTTCTTATTTAATGAAGTACCTGAATGCAGGTCCACTTTGACTGCTTGTATCGGGGGATTACAGCCGTGTCAAGCAGGGAATCTAAACTCTTGGCCGGAAATCGTAGTATCGCGCAGGTTCTCAACTCTATTCTTTGTTTTGTGGGTGTGTTGTGTGTGTTCATATCATGCACTGCCTCTTTTTGAATGAATTACCAACGCGTAATTCCTTGTTTTGTACTAGCCATACGTGTTCAAAGTTGGAGCTACTCCGGGGAAAGAACGAAAGCGTGAGTTTGTGGACAATGCCAATGGTCTATTTTCTGCACAAGCTGCACCAAAACCCCCTCAAGCAATGTACTCCGTAACTGAGGGGATGAAAGTCGGGGGAAAGGTGATGGAATCCTCCTTTNATGCTTAAATACAACATATGTGGTACTTAATCCATCTAGTTTCATCGTTTCCATGCCTTCTTTTCTCACCTTTTAACTTAGAAGACTTTTTGTCATTGTTTTCTGGTATCCTTGCAGCGAACTGTGATTGTTCCTCCAGAGGCTGGCTACGGCCAAAGGGGCATGAATGAGATCCCTGTAAGCGCCTCTAAACATCGATCATTTGTTCCGCTTATTAGTCTTGACAGTTGACTCCAAGACCTTACCCTAGGGCTAGGCATCACAACATCTATCATTCTATTATTCATTCATGAGAGCATTTTCGTACTGCAGTGCTATATCGTTATTGTTTAATACACTGCTTAACTCNAATCTTTGTTAATTTTGGACAAAATCTTCAAAAAATCTGAAGCGTTGTTTGTTTCTTAGACAGTCCTGATCGATCATGTTAAAANTTGTACTAGCTAGCTTATAGTGCNACGTACATGTGCAAGTCACTTGCTGTATATAACAAAAATGTTGAGCATACGATATGGAATTTGACAATGAATGTTTTCCANNCAAATCTGCAGCCTGGTGCAACGTTTGAGCTAAATCTGGAGCTTTTGCAAGTAATTCCGCCTGACGGAAAGTGAGCCCGGCATAGGTTGATTACGGTAGTTTATTTTCGCTCGACGATCTCCCGTCTCATGTTTCGGTGGGAGGATGGGGATGAGCTGGAAGTTGAAACCGCCATCTTCAAAGAGTAGGGATAAACTTGAGAGAGACTTGTGTACGCAACGGAAAGTTTGTAAAGGTCTTATGGCTCATATCATTGAATCTGCATTTTCTGTACGTAATCTGGACGACTTTAGCAATTTATCATTGCTGTATGAAACTGGCTCGTTTGGTTAAAATGGTAACGCAAATTTGTG

>MdFKBP19

GTCTCTCCTCCCCTCTCATTCCAAATTTATCTCTCTTTTCTTCTTTCTCTCTCATCTTTTATTTTATTTTATTTTTATTTTTACTTTTTTCGTCTCTCCTCCAATCTGCTCTCTTTATTCTCTCGGTTCTTTCTCTCACTCCTATTCCTCTTTGTCTCGTCCGATCCTCACTCTTCTTTCTCTTTCCTTCAATCATCTATTACTTTCATTTCTCATCTCTCTCCCTCTTTTGCGACCCCCTCTTTTTAAATATATTTGTCTAGTTTAAATCGTAAAATTTAAAAATTTTAAATCACAAATCAATCAAGTTTTTGAGTCTTAAAGAAAATTGTTTTCAAGAAATGTTCTTAAAAAATATTTTGAGAAATGATAAAAAATTTCAAATAGGATACCAAACAAGTCCTAAAATTCTTGAATCCGATTGCCGAATTTTAGGTTACCTTCAAACTAATGGAGAGATTCAAGTATACAGTGTATGGACTTCCTTGTTTTCATTTCATTTTTCATCGAAAGTAAACGCTCTTGTTGAACTATGTACCAAGGGAAGAAAGAAAATACACATAATTAGAAAAATATTATGTTATTGAATTATCAATCTGAATTGTTGTTGAAATATGTTATTCGTGACTGTTACTTTGATTGACAATATTATTCGATTAAAGGAAAGAAAACTGAACTTAATACAAGTGGCGAACACCTTGATCTAACTGTCTGGTCTAAATCAGCCAAAAAAAAAAAAAAAAACTAAAACTTGGTAAATGTTTATTTTTGTTACACGTCCAAATAAAATTAGATAGTGCTGATTAATTCAAGCAATAGGTTAGTCATAATAATTTAATATTGAATTTTTTATTTATAGCATTACATGAAAGAAAAAAAGAACTAATATTATATACATATTATTTAATTTAAGAGTAAATTGTAGTTACGGTCCTTTAACTTTAACTCAATTGGAGCAATGGTCCCTCAATTAAAAATTCATTACCATTGGTCCCCCAACTCATCAAAAAGTCCAGCTATGATCCCTCAACTCAAAATTCATTATCATTGGTCTTCAACTTTAATTCAATTGGAGAAATGATCCTTTAATTTTAACCCAATTGTAGCAATGGTCCTTCCAATATAACTCATTTTGACAAAAATTTTGACGTAGTTGACGAAAATGACCATAATTACACACTTTGATGAGTTGAGGGACCCTAATTATATAAATGGTTATTCCAACATAACTTATTTTGACAAAATTTTGACGAAATTGATGAAAATAACTATAACTACACATTTTGATAAATGAAGGGACCAATGGTAATTGAATTTTAATTGAATGATCATTGCTACAATTTACTCTTTAATTTAATTTAAAGGAGAAAAGAAAAATGAAAAATAGAGGGGTTGAGCCAGACAAGTTGAGGCTGTTATCTTGCCGCGAAACGCATTCCCTGGTTCCCCAGTCACCACATCACAGTCCCACCCTTTTCTCTCCCTAGTCGAGTAGCACCTGCCACCACACCTACAATGTCTTCAATCTCAGCCGTCCGGTCTCCACCGCGGCCTCCGTCCTCACCCGTCGGAAAATCTTCAACCCCGATACGTCGTTTCGTGCTAAACTTCCCTTCTATGCGCCAGAGACGCCGCGGACCCCAATCTCATCTTCTTCCTGAAGCGGAGAAGTTTTCCGATTCAGGTGCAGGCGGTAAGTACATCAACCATGGAATATCTATTTTTAATTCGTAAATTGGCCAAATTTTGAATGAATTTTCAATCTTTGATCAAGGTGGAAGCATTCTTATCGAGCGAAGAAGTGTTGTGTTTTCATCGGTTGGCATGGTGGCTGCAGCTTTCTGTAATGCTTCAAAGGACGTAATTGCCCTCGCGTCTCAGTTTACTGACAGTGAGTCATTTCCTATTTCCATTGCCCGAAAGGTTAGCGATTTTGACACTCTCCTGTTTTAGCCTCTGCAATCCTTCTGTGACTGTGAGTGCCAAAACATAACATCGTAAATTATTCAGTTTCGAGTGCGGATGGCTAAAAATACGGGGAGTGTCGAAATCCCGTCCTTGAATAACCATGCTCATAAGTCATAGTCATATATATAATATATTTGTATGAGTGCAACGAGTGTATATCCTGTTGTTCGTATCTCTCTCCTCACGTGACATATCATGTGATGAGATAGACATGCACAAGTGTGTCACTTGAGGAGAGAGAGAGTGGCATAAGACATATCATGCGACGAGAGATATATATGTACTTTTGCTTTTTCTCACATTGTTTGTTGCATTTGTGAATGTTTGGGAACCACTTTCAGTGCCAGCACTTAGGGGGAAGGACTATGGCAAGTCGAAAATGAGTTATCCGGACTATACGGAAACTGAATCAGGTCTTCAGTACAAGGTTAACTACTACTGGAACATACAAAAGTTTAGCCTCCTTGTTTTTATTGAGTTTCCAGCTCTCCGAAGTTTGCTTTAGTTATTTCGTTGAATGATTTATCTAAGAAGGAATGCAGGACTTGCGAGTAGGAGATGGCCCCAAACCGAAGGTGGGAGAGACCGTTGTGGTATACCACTCATTCTCCGAGTGCACACTTTCAAGCTTTCACATGTTGCTAGGGTTTCTGTGTAGGATTAAGCCAATTTTGTGCAATGAGTTTGTCTCTGTCGTATACAAACGTTTGAGCATATTTCTGGTTGATTGGATGGATTTCAGGTTGATTGGGATGGATACACCATAGGATATTATGGACGTATCTTTGAAGCTCGAAATAAAACAAAGGGCGGTTCATTTGAGGTATGGAATACTTCTCATCGGTTAACTTGGTATTGCCTAAGCTTAACTAACCGATTAACAGATTCCATTCCACGGTTTCTCAAATGCAGGGTGATGACAAGGCCTTTTTCAAATTCAGGGTAGGATCTCAAGAGGTGAGAAATTTGTGACCCAATCGAACGAAACAATCTTGAATGCTCTGATCTGTGGCCTCTAATGATGACGTGGTTATCTTCAGGTAATACCAGCTTTTGAGGAATCCATTACAGGCATGGCTCTTGGAGGCATTAGAAGGTGCTGCATATTCTTTTCCGTAGCTCGTTGTATTTGCTCAAGGAACTTTCAAGTGGTTACATTGAATGTTATTTGCAAATTTCACAATGTAGCTCACAGTGATGCAGGATCATAGTGCCCCCGGAATTGGGATATCCAGACAATGACTACAACAAGAGTGGCCCAAGGCCGACAACATTTTCGGTAAATCTTATGTCTTTCTGCCGATTCATATAATCCAGTTCCGCTTTGTTCTTACAATGTGCAATGTTCACGCGTTACAGGGCCAACGAGCCTTGGATTTCGTGCTGAGGAACCAAGGGCTGATCGACAAGACTCTTCTGTTCGATATTGAGCTCATCAAGATCATACCAAACTGATCTCCTTTCAAATTCAACGTTTCTCACAGGAAATCGATTAGCTGTTTAACAAATACGGCTTATTTATCGTAGAGTTTCTTATTCCGGATGCCAGAAATATCGATTAGCTGAGAAAGTTGACGGATTACCATAATACCATCAGATTTGAAGTAG

>MdFKBP20-1a

TCCCCTTAAGTATTAACCTTTTGTCTGAAATTTCCGTTGGGCTACAGCCCAACCCAGCAAAGCCTCCATCCGGATCTCTGCTCATGCAGTATCCAAATCATCAATGTCAAATGAAAAGGACGGGTTGTTCTTGTTTGTAACCGAACAGGAAAAAGAAAAGAACCGGTTTTGGGGCTAGAGAAAAGAGAAACGGCGGCTTTTAAGTTTTCAACATGAATCCAACTACTATTACGACTTTTTTGGGTAGGAAAGGGATCCTATCCGGATCACTTCATCCTAATCCACTAAATTCAGAGATCCGAGTCGTTGAAATCTGATCCAACTGCTAAAGTTATTATAATTTTTAAAGTGAGCCCTTATTTGTAGCGGTTGGATCAAATTTCAATGGTTCAGATCCACGAATTTAGTGGATTAAGAAGAAGAGATCCGGAGAGAATCTCTTTTTCTGTTGGTCGGCAGAATGTGATGAGTTATATCCAATTAAATTGGACTGGTAATCAAACAGATGTTTGTCGTTTTAATTGAACATAAACCAACCGATATTTGTTGGGTAAGAACGTAACTTGGATATAATAGGACAAAATAGGTTACGAATCTCAAATAAAGTTATCTAACTTACCTCATAGTCTCCCACACTTCAACGTCGTCGAACAAATTTTCGCATGTTCTCTCAACTGCAATGACAAATTTAGATCAGATTTTTGCATGTGTGATTACTGATGATTAAATTATTAACTAAATGTTAATTAATGTACTTATTTTTATTAATAACACACCATATAATTTATAATTTTGATCTGAAAAATTAATCCAATTGACCTCCCTACCATTAATCGTGGTACAGTAAAAACTTGAGCGGTGTTTATTTATACTCTTCCCATCATTTCAGCATCTGCTGGAATCGCTACACGTGCGCGTGGGAATTTCATTGTCATCATCGAGTCATCGTTATATAAAGACGCCATCTTTCCGCGTCGAGCACATCCTTCATCAGACGCCGATGGCCACCGAAGCTCCCGGCTATACCACCCGATTTATCGTCTCCCTTTCAACCCGACGACCCGATTGCCCCTCTCCGTTTCCGAAGCTCCTTTATCGCCTCGAAACGGGTCAGTCCAACTCCAACTCTCTCTCACTCTCACTCCCAAAATTACTGATTTTTTGTATTTATTTTATTTTATTTTATTTTTTTCTCACCTTGTTAATTTGAGATTGAAGTAATTGGTGGATTTTAGTTAAAACCCCTAATTTTATTAGTTTAGCTTCAATTCCTTGTTAATTTCTGGTTAGAACAACAAAATTTAAAACTTTTGGGGGTTTTTGTTTGTAATTGTATCTAAAACCCACTAGGGTTTTGAAATTTTTTATTAATGTCTTGCAATTTATCATTAGTTAATGGAAATTTTTTTCCAAAGGTACAACCTTTCTGGAGGGTTTTTTTATGCTAAGTAATGATGTGGTGGATTTTCCTTTTTCATATCCATTTTATCATGTCATGGATTCATGTTTGTATGTCTATGACATGACCAGAACATTGAAGATTGCAACTGCTGCAGCCATGAGTGATGCAATTGATTTATCCGGGGATGGCGGTGTTCTCAAGAAAATTGTAAGGCGTGCAAAGCCAGATGCAATTGCTCCGACCCAAGACCTTCCGCTCGTTGATGGTAAGGGCTAGAGTGTTGTTGCTGATCATATTTATTTTTCGTTTGTGCAGTGCGAACATTTTGTGATTAGGTTGAGTGGAAATACCTAGCAAACTTATGTTCAATTTGTTAATAATCATATACATTATGTTGGCAGTTCATTATGAAGGCATTCTTGAGGAAACCGGCGAAGTCTTTGACACGACGCACGAAGATAATACAATATTCTCATTTGAGATTGGGAAAGGCAGTGTGATCAAGGCTTGGGATATTGCAGTGAAAACCATGAAGGTTTAAAATAAGTTTCTCTTTGAGGATTTTTTTGTGAAATTTTCAAGTCCCCTATTACATGTTTTCTCTTTTGATTGTTTCCTCAGGTTGGGGAGATTGCTAAGATCACTTGCAAGCCAGAATACGGCTATGGAAGTGCTGGTTCTCCGCCAGATATCCCACCCGGGTAATGCCATTGTTCTATTCATTATTTTTCTGTCAATTTTTTTTTTTATCAAGGGACACTAGGAGTGCTAGTAGCTTTGTTAAGTTAAAAATATGTTTGCGCACCGATGTGCCAAAACAACACTGTCCAGACATAATTATCTCCCAAATGTGGATAGTATATGCTTATAGTAAACAATTGCATCTTAGTCTATGATTTAAGAGTTATTTGGTTGGTGATCTTCGATTCCCAACTATAAACCAATATATTTGTTGAATAGATGTAAAATCTCCGGAAGCAAGAAATGTACATAAATTCCAGGTGGCAAGACAAGGGAGGGTCTTCACGCTCGATACTTGCTAATCTGTGCGCATTCTGGTTTATGACTGTATCCAAGTGTTCGTAAAAGTCTGCATCAATTTTCTTGGTCAATCATGTTTTATTTCTTGTTACAAATGGTGTCTCTCTACAATGCCTTTGGAATTAATTCATCGTCTGTTACAAATTACTCCTGCAGTGCAACCCTTGTATTTGAAGTGGAGTTAGTTGCTTGCAATCCGCGGAAGGGATTAAGTTTGGGTAGTGCTTCAGAGGAAAGGGCTAGGCTAGAGTAAGTCCATTTACTTTCATCTTCTCCATAACTTATATTCCAGATATATAATGCAAGTCTTATTGTTACCGTTGAACTTTTTGTTGGTTCCGTTGAGATCTACCAACTTTTTTGTGACCAGTATGATTGATTGAATTTTACGTTGGTCCTCACAACAGAGAACTGAAGAAACAGAGGGAGTTAGTTGCTGCAAACAAAGAGGAAGAGAAGAAGAAGAGAGAAGAGGCCAAAGCTGCTGCCGCCGCTCGTATTCAAGCAAAGTTAGATGCCAAGAAGGGCGGAGGAAAGGGAAAAGGGAAAGCAAAATAGGCACTTGGTCGCATACTCGCATGTTTGCATGAGCTTGATCCTTCATATTTATGTAGATACAATAAATAAATTTAATTCGCGAGTTAGACCGAACTATGTCCTTCATAAATAAGTTATTTTCGGACCAGAAAAGATGGTATTGATGTGTTATTGAAATATGAGGTGTTGAATTATTACATAATCTAATGCTGAATTATTGCATTTCTTTACTG

>MdFKBP42a

TATGGAAACGCAAAGCTGATGCTGCTTTCCAGCGTTGCACGGTATTATTTTATTATCCTTGTATACTTATTAAGTTCCACTTTTAGGATTCAATACATTATCTAAATTCAGGGGAGGTTTCTGAAACACAGGACTTTGAAAGTTATAATCGATCCCCAATCAAAGCCTAATATTCATTCATTATTTTCCGATGGTTTCATTTACGTCCAGGGGATTATGTTGTTCCTGAGTTGCTTAAGTTTCTGATATGGCTTTTGAACCTTGCAGACTATGAAAGAGTTTTTTGATGAAACGCTTGTAGCAATTCCTGACTATGTTTTGGATAAACCTATTGGGGAGCCACCATCACACAGTGACAATCCTTTCGCGAACATACACAGTTTGCTGGCGTATGAATCAAGGGAACTTGAACTGCAATATGCTTAGCCGGTGTCTATTGAAGATACATACTTCTGGCGGCGAAACCCGTTGTCAAATTTTTGGAATGTAACATGATTGATCCCAGGACCCATGTAACAACATTCATCCTACATGTCAGTGCGTAGATAGTGAAAAGCATATATCTTGTAAAGCAGTCGCTTTTGGATGATGACTAGCAATTTGTGTTTTGAGTACTACATATCTTGCTTGTATACTAAGAACCTCGCTCTTAACTGGAGCAATTTGTTCATTGATTTCGTAAGTAAATCCAATGACTTTCAGACGTCGATCTGTTATTTCTGTTTGGTGATTGATAAAATGCTTGTTTTGTGTCTCGTCACAATGTGGAACAGGCGATCGACTCTTCGTTGATAAAAAAACTGGGAAACATGTTATTTTATGCTCTTCAATTAACTACGGATTTTGGAATTTAACCTGTTAACGTCTATTTTATGCAGTCTTACTCTTATTTTACAAAAAGACTCTGTTATATGATTTAAACCCGTGACTTTTCGATCGCAAAAGAGAAAAATTTCTGTTCCAAGGCTCCTCCTCAAATATCTGTAACCAAAATTTATTCCGTTGAAGTTTGTAATTTTTGTTCCGTTTTTGCTTTCATTATTCGGAAAATGATCATTTTTCTAGGAATCTTATGATCGTGACCGTTTATCGTACATTGTGCGATTAATTTTTATTTGGTACTATTTATTTTAATTTTAAATTTTAAATTTTGAAATAATTTCTGATCGCACGATGTACGATGAACGATCACGATCACGATCACGGGATCCTTATTTCCAAAAAGGATCATTTTCCTCATATTCTACGAAGGTAGGAGACGAACAGAGGTAACCTCTGTTAACTGAGGAGACAGGTGGCACAAAGAGGGTGCAGGCAGAGGGTGGGCAAAGGCTTCTTGGGCGCAGCAGATTTGAACTCGTATGATCAATTAATTAATTAATTAGTTGGATGTGTCTCTAGAATCTTGTGCGATCGTTGAAAACCAAACACAATACAGATCCTCTCACTCTCTTCTCGTCTCTTCTCGTCTGTCCAAGTGAGTCCCGCTTTGCCCTTTTCTACTTTCCGTTTTCTGTTTGTTTCCCGGGAAAATTCCAAAGAAAGCGAAACCCTTCCTAATTTTCGGTTTGGAATTCGAATGCAATTGCATTAAATTTTGCTTCTTTGATTTGGGTATTTGCAGAATTTTGAGAAGACCCATCGGATTGGATTATACAAGTTTGAAGTAATGGAGGATGTTCGGGANCAGGAGCAGGCTGAGCAGCAAAGTCAACCACTTGGTATATTTACTCATACTTTTGGCCTTAATTTCCAGTTCAAGTATTAATTCATACTTTTNTACNCTCAATCAAATCTTTCAGCTACACTTGGTTTCTGTNGGAGCAAATATGTGCCAAATCTTAGTAGGTTTTTAGCTTCNAATGTATGTCAAATTAACCTCATACGCTGGTTTTCGAGTGATTGACTTCGACTGAATAAATACATGAAGATGTTTGCTTATCCTTTTCTACTTAGTTTCTTCAAGAACAATTCTTCTGTAGGTCAAGGCGGAAACTTATTGTTTTTATNTACTTATCGATAAGATTTTNGAAATGATTTGTATGTCGAGAAGGATTGCAAGGGTTGGACATTATGACAACTGCTTCCAGTACACTGATCCATCATTCTTCCATGCTTTTATGTCGAGTAACTCAGAAATCCTATGTTCCAAAACCCATGTATTAATAAGTGGGGATGGCAGCCGTATAGACCATGTATAATGTGCGGGGGAAAGAATAAGATTGTCGTTCCCCCTCTGCTTTTGAGTGAATCAGATTTCCAGATAATCATATATTATGGGGCACTATAATTCCTATAGGGTATTGCAATCCATGTGAGCCTCAGAAAGGGCCATCAGAAGATAGACTAATGCTCATCTTTTCATCTTCTTTGTAGGTCAAGATGGTGAAAGTGAAATAGTAACCGAAGATGCTACCCTTGTGCATGGGGAACCTTCTCAAGGTGCTAATGGCCCCCCAAAAGTTGATTCTAAGGTGGAAATCCTTCACGAGAAAGTCACAAAGCAAATCATTAAGGAAGGTCATGGTCAAATACCATCCAAGTATTCAACATGCTTCTGTAAGTGTCAAGTTGTTGCTTATTCTTGTACTTGTAATATGGTAATCTTTGGATAGTGCTGTGTACATTAGTTGAAAATCCAGTAATTGTTATTTGTGCATGTAAAATTTTACTGTGGGAGTAAGACATGAATGGGATTGTGACTTGCAAACAGACACACACAACATCCACGTTCGCNGAGTTGTGCGTGCGTATAGGAGAAGTAATTGTGCAGGCATTAAAATTTATAAAAGGATGCCTAAGAAGGCTAAGATACATGTAGACAGGTCTTGTAGTGAATTTTTACTCTGGTTTTTTTAATCACTGATTGATTGGCATATGTGTGATAGTGCACTACAGGGCATGGACTGAAAGCACGGGACACAAGTTTGAAGACACATGGGATGAACAACGACCACTTGAAATGATTTTAGGAAAAGGTATTAGCCTTGCCGCGTAGTCTGTTACTGTTGATATATACAAAATATATGCAAACTGAGTTCTGGCGTGTTCCTTGTTCAGCCTGCAATTAGTTTTGTGGTTTCTTTATTGCATTTATTCTCTGTAATTACTTGTCATTTCNGATGATTTGGGTCCATAAACATTTAAGCATTTAAGCATTATGTAGTGGTATTAAATACAATGGACTTCTTATTAGTAGTATATTTTTGTCANTGCAACAGAGAAAAAAGAAATGACTGGCTTGGCTATTGGGGTGTCAAGTATGAAGTCTGGAGAGCGTGCCCTATTACATGTAGGCTGGGAGTTAGGGTATGGGAAAGAAGGGAGCTTTTCTTTTCCAAATGTTCCACCATTGGCAGATATATCATATGAAGTTGAGCTTATTGGATTTGATGAAACCAAAGAAGTAAGTTGCTCACTTGCTCTCAAATGTCTTCCCAAGTAGTTACCAATGGAAGTTTCAGAATCTCTTTTCAGGGACTTGCTCCAATAATATAGGTTAACATGTATATCTGTGGGTTCTGCCTTTCATAGGGGAAAGCTCGTAGTGACATGACCGTGGAGGAAAGGATTGGAGCAGCAGATAGAAGAAAGATGGATGGAAATGATTTATTTAAAGAGGAAAAACTAGAGGAGGCAATGCAACAATATGAAATGGTAACGCACTTGTCATTCTTACATATTTTATTTATGGCTGANGAGCTTTTTAGTTGTAATCTATTTTCTAAATTTCCCTTCTGACAANATTCTGGTGGTTTTTCTCAGGCCATAGCATATATGGGTGACGACTTCATGTTCCAGTTGTTTGGGAAGTACAGGGACATGGCTTTGGCTGTTAAAAATCCATGCCACCTTAACATGGCAGCGTCTTTTATAAAGCTCAAGCGCTATGAAGAAGCCATTGGACAGTGCAGTATTGTAAGTATAATGCTATCACAAGAAAGTCATTTAATTTCATGTGTTCAGCNATTGTGCTATTCATCATTATACACTTAGAAAGTTGGGTTTGTAGGCTTATTTGGCATTCTGTGAATTTNGAATCTTCATTCACTCTAACCTGCCTTTTGGATTGCCATAGAAATGAAAATGCTTTTAGTATGGACCAGTGAAAAATTTGTAGGCTCACTAGCACCATTCGCCAATCAATAATCGAATTTTGGGTCCNATGCGACTCTTATGATCTCTAGTATGTTACTTTCCATGTTTTATCCTGTTGTTCATACTATATGGATGTTANAACTCAGAATTGGCAAATGCAACTAACATCTTATAGTTGATTTTTCTGTGTCGTTTTGTATGGAGTCTGCTGATATTTACCTTTTCATGAATACCCATAAATTGTCAACGAAACTAACGTCTATTATTGATTTTTCTGCATTGCTTGGANTCGGCAGATATTTACCTTGTCATGAATACCCTTAAATTGTCATNGAAACTGTGTTAGCTGTAATTGGATGAATGTTTGTAGGTACTGGCAGAGGATGAAAACAATGTCAAAGCGCTATTTAGGCGAGGAAAAGCCAGAGCAGAGCTTGGGCAGACAGATGCTGCGCGGGAAGACTTTCTAAAGGCGCGCAAATTTGCACCTCAAGACAAAGCTATTGCAAGAGAGTTACGTCTGCTTGCTGAACACGACAAGGCTGTTTATCAGAAACAAAAAGAAATCTACAAGGGAATTTTTGGACCAACCCCAGAGCCAAAACCCAAACGGAATAATCGGTTGATCATCTTTTGGCACTGGCTGTTGTCATTGTTCTATCGTCTCTTCAGGCTCGAAAGGCATAAGGCTGAGTAGCCAAAGATCTTAAGCTCTTGAATTGTATGACAAACGAGGAAATTCATGTACTGAACCTATTGACCCTGAGGTTCGTTCGTTTACGTTGCGGGAGGGAGTTTAGATGCCAACTTTTTTAGACAGGAGCGAAGATATATTGATCAAATTGAACTGTTCTAAACTATTAGACAAAAGTTTGTTTACTGAGTTTCGGTTGTGAAATTTCCATCGCTGCCATGATCTTTTAAATGGTGTGTTTAATGCGTCAATTTGTTTC

>MdFKBP43
CAAAACACAACATTTTCTCTCGTCACATTTGCTGGATCCTAGATGTTCCTTTTGTGGAACAGAGGATGAAACTTTAGAACATATTCTCCTTAAGTGCTTGTGGGTGCACCATGTGTGGTGCGACTCTCACTTATGTTATCGCCCTAATAGTCAAGCAATTACCTATCTTGATCCCTGGCTTCTAAGCATGGCTACGATGGGATCTGCATCTTTATGGGATTCTATCTAGGTTTATATTAAGGGTGATGTGCTGGGAAATTTTGAAAGAGCAATGTCATGCAGTTTTCAAGATGTTGGCTCCCAACCCTATCTCTGTCGTAGCAAGGTGCAACAATCTGTACATTGAGATAACAGATATTTGCATAGGCTTCCATCAATTGGTAGTCAAGAGGATCATTCTAATCATAGGTGAATCCTTCCAATAGACATGAACTTAAATGTTAGCATTAATGGGGCTTGGAACTCTTCTACGACAGAAGCGAGTGGGAGTTGTTATTTGAAATTCTAGAGGTGAACTCATTTGTGGTGAGTCCAGTTTCTTGAAGAGGGCTTTAATTGAAGAGGTTGAAGTTGAAGCAGTGTTAACAGTGACGAAACTTGCTTCAACAAAAAACTTCCAGCATTATTACGATCGAGGGTGATTCTAAGGTGCTTTTAGAGGCCATCAATAGTCCTTATCTCAGCAAGGTATGGAGAATTTGCCCCATCAAGTAGCTCATTTGCCAGCTGCACTTGCCAACTCGAGAGTAAGACACTTCTAATGGGCTTCAACGCCCCCTCCCTCCTTGGTTATGGTACTTTCACGTGATGGACTCCCTTATCGTCCGTCATTGGTGGTCTGAGGATGGCTAGTAAAGTAAGCCAAATAGGACTAAAAGTTATCAAGGTGTTTTCTAACGAAGATGTTATATATGATGTGATAGAGTCATTTGACTTCTAACTATCTTTTTCTAGAGCAAACAAAGATGAAGTTAACATCTCTCTAACAAAACTATCGTTTGACAAAAGTAAGTGTGTGTATAAATGTATATTTAATTTAATTATTTAATTATTTTGGAACATTCATATGCTCTCTTTTAATTTGTTCCTATTTTATTAGGGAGCATTTTTGCTCACCACCATTTAGTGTGGTGTATGCTCACCACCCTAGTTATCACAGTTAAATAAATTTAAATTTCGAGATTCGTGTAATAGATAAACACAAATCTCAATATTCAAACTCATCTAACGGTGATAAATAGGATGGTGAGTATACTCCACCATTTAATGCACTCATTTTATTACCAATGTATTAGTGAAAAATTTCCTCTTTTCCCGCCCGGCGCTGGGGGAGGCTACCATCACCAACGCTATCAAGGTAACCTCCTATTAGGGATTGCCACGTCAATAAAAAGTCAGACCGCAGGCTTGCAGCCGCCGTCTCTGTATTTAAAAGGATTGTTCTAGGTTCTCTCTTTTAATTTCTCAGCAGGGTTTAGGGGAGGAGGAGGAGCAAGCAATCCGGGCCTTTTTCTCTAGGGCTGTAGATATCAGTCTCTCCCAAACAAAATGGCTTTCTGGGGTCAGTTTCTCTTCCCTTGTAAATTGAAAATGATCGACGATGATCAACTGTTTGAATTTACTTATTTGGGTTTTCTGTTTAAAAATGAATGGAATTTTATATTTTAGTAGTTAAGTTTTGAAGTTTTCGTTATATCTTCTGCTTGGCTTTTCGGAGCAACCAAGTGGAGGTCGACTGAGTTGTTATTGTCAAGTGTGTTGTTTATTCATTGATTATTACAGGAGTCGAAGTGAAACCCGGAAATCCTTTCACTCACATATTTGATGGCTTGAAAGGACGCCTTCACGTTTCAATGGTAGTGCTTCTTATCACAACGACCCCTTATTCTTCTTTGCTTTGTGATTCTTCTTCTTCTTTTTTTTTATTTTTTATTTTTTATTTTTTTAAGCGTAGAATTTAGCCAAAATGGGCATTGCTGTGCGACCTACAATTTATAGTTTTTTTTTTCTCTGTTAGGTTTTGTTTTTCTGCTTTTGGTTAACTGTATTTATAATTTACTTCCGCTTTTACGCATGTGAAAATTGAATATGGTCTAATATTGAGTCATCTGAACTTGGTTTCTAATGTTTTGATGTGTTCTGATATTTAGTTCCGATTTAATGTTTAGATCAGTATCGTATGTAGTATTTATAATTTTATATACACAGGCCACACTGGGGAATGGGACTGCGACGAAAAAAAGCACACTTCAGTGTAATGTGGGAAACAAGAGTCCTGTTTATCTATGCTCCTTGTTTCCTGAGAAGGCTGAGTCATTGCAAATAAATGTGGAGTTTGAAGAGTGTGAGGAAGTCATCTTCTCAGTTCTTGGTCCTCGGAGTGTTCACCTATCTGGCCACTATCTTCCTCGTCACCAACATCACAATTTTGTGATGATAATTCGTATCCTTTTGTTTAGTGTGGTTATTGTTTAGTCCATTATCGTTTCTTCCGTGCTTAATACTTTTGGTCAAATTTCTCTGTTTTTACTCCATCACTACCTATCTAATTAGTTAATGGGAAAGGAGAGAAAAAAACCGTAGGACACTTTTTTTTTTCTTTTGCAAAAAATGGGAACCCAAGAGACTAAGAGACTAGTTTCTTTTTGGGGGGATATTTTCTATCCAAGATCAACGCTACATAACCCTTTTGGTTATGGACATTTTTCATCGTTTGTCTCATGTGAGGTTATCTTTAGTTTCCCTTTACTATGATTTCAAGAGAATCTTATGGGGAAGATATTGCTAATTCCGAAACCAAGATGTCTGTTGATAGTGAAGAAGACAAGTATGAAGATAGTTTTATTAATGATGATGACCCAGAAGTCTTCCCAGCTTCTCCTGTTCTTGATAGTGAAGGTACGGCTACCAAATATATGCTTTAAATTTTTCTCTCACAAAATGTAGTATACATGATGTGAAAGTTTGGTCATTAGCAGTAGCTCCTGATACAGGAGTCAGCTGAAAATAAATAAAGCCCAGTCAAGTACAAGCTCCTATAATTAAGAGTCCTAGTTTTTTTTTACGTAACTTAAATCATCGTTTTGGTGCTTGTTGTAGACAACCTGCTCATGTTCATCTACTCCGTGTTCAGTAGTGTATGTCACCAGTTTTTTGGTCTCTGCATCACATTTGTTCAGTTTTTTGGCTTGCATCTTTAGTTCATATCAAGTTAATGTCCTCTTGAAGATGAATCATTGGTGAATTTTTGCAATACTATTCTGAGATATATCTATCTGGTGTGTAGAGGAGCTTTTGGACGAGCATAAACCTCATAATGGCAAGGGCCATCGAAGACGACTTCGGAAGATGTACCAAGTGAGCGAGTCCGAAGTGGAGGGTGAAGATGATGACAGATGCCTAATTTCCTCTTTGTACAAGACAAAATCCAGTACTAGAAACACAACAGAGGAAGTTGAAGAAAAAGTTGACAAGAGAGCCGCAGATACCACTACTAGCAATGAAACAGAGGATGGTGTCTTTAATGCTACTGAAACAATGAACGATGGTGATGATGTGGATGTAGATGGTCAGTTGAAAAGGTATTTGTACTGCTTAATAAGACCATGGAAGCTTGTGAAAAAGATTGATCTCTTCCCATCAGTTGAATATATTTTCATTGTACTTGTACCAAATTGAGTTATTGTTGTTTGTCATCTTGATTGCCAAATTGGAATGCAGGCAATTTGACCAGCCAGTAGATACCTTCCTACCTTCTTCTGAAGAAGGCCTTGAAATTGGTGGAACACCAAAGAAAAAAAGGAAAGAACGGCTGGATGATAAAGCATTTGAATCTGGTTTTCGTGAAAAAGAGGACGAAGCCCAAAATGATGACACCAAAGTTGAAAATAGGACCCAAAATTTGTGTGTGGAGGATGGACAAGAACAGAAGGTGGCCAATCATATGTGAGTTCTTTTTGCTTCATTTTCCTTGAATAAGTACATTCTGAATCATGAATCTTAAGTGTGCATACTATTCTTTAAAGTAGGAGAATTTACAGACAATGATGTTTGTTGGTTTTGATTTTCGTTATATTATTGGTGTGCTCAAGCAGCGCACTTGAAGGGCCAGATGATTTTTCAAAGCCTTCAACAGAGGTCAACCTTGAAAATGATGGAAAACTGAAGAAAAAAAGGAAAGAGCGGCCAGAAGATAAAGCACTTAAAGCTGGTATCCTTGAAAAGGAGGATGAAAAACCCCAGAAAGATGACACCAGAGCCAAAAATAGGATCCAAAACTTGTGTGTGGAGGATGGACAAGAACAGATAGTGGCMAACCATATGTGASTKCTTTTTGMTTCCTTTGTCCCCGTATAATTACATTSWTACCTCWTSMYACTYAAKTGWAYATAYTATTGTTTAAAGTTGGAGAATTTACAGACAATGATATTTGTTGGTTTTTATTTTTGCTATATTATTGGTGTGCTCAAGCAGTGCACCTGAAGGGCCAGATGATTTCCCAAAGCCTTCAACAGAGGTCAACCTTGAAAATGGTGGAAAGCTGAAGAAAAAATGGAAAGAACGGCCAAAGGATAAAGCACTTGAAAAGGTGGACGAAGCCCAGAAAGATGACACTAGAGCCCAAATTCAGACCCAAATTTTGTGCGTTATGGATGGACAAGAACAGAAGGTGGCCAAACATATGTGAGTTCTTTTGCCTCGAATAAATACATTCTCAATCATGATACTTGAGTGTGCATATTATTTTTATGGATGGAGAATTTACTGACAATGATGTTTATTGGTTTTGATTTTTGTTTTATTATTGATGTACTCAAGCAGTGTACCTGAGGGGCCAGATGATTTGTCAAAGCCTTCAATAGAGGTCAACCTTGAAAATGGTGAAAGGCCAAAGAAGAAAAGGAAGAAGCGTGTGGAGGGGAAAATATTGGAGGTTGTCTGCACTAACCTTGATGATGTTGTTAAAGAGGATAAAGGGCAGCAGGATGAGGCAAGGGGTGGCATCTGCCCAGATCTACCTGTGAGGAGTGAGCAAAATCAGCAGTCAGCCAAGGATGGGTGAGTTATTTTTACCTCACGTTTGTCTCTGTGTCACAAGATATACTTTTTGCCCCCTTGACTCCACCTCAGTCTTGGGAATAGCATGAAAGGACATGTCCATTGAAATGGAGTGTAAACTTTAGTAAGCGGTTCCATTGATCAAATGTATTGTCTATTATTATCTTCTATCAGGAAGTTAAAAATTTCTCTCCCTCACCCAATTCCAATATCTATGCGATTTTTAGGCCATGTCTGGTTGTGTGCGATTTTATTATAGTTCCCTTGATTGAGTATAAAATCTGTATGTCCATTTTTCCAGGAATTCTGGCCATGATTCTGGTAGATTTGTTGATGGTCAATCTGATGAAAAGAAAGTTAAAAAGAAGAAGAAGAAAAGTAAAACCGAAGCACATGAGGTAGCTGGGAACACAGATGTGCCTCTCTTGTCAGCAGAAGATAATGCTAATGCCAAGTCATCTCAAGTTAAGACCTTCCCAAGTGGATTAAGAATTGAGGAGTTAGAAGCTGGAAAACCAGATGGCAAAGTTGCCACATCGGGGAAAAAGGCTAGTCTTTCTGAATTTGTATTTGATCTATTTGATATTGATGCTTTTCTAAAATAAATATTTGGATGTGACCCTCCAATGGATAATGGTAGCTTAGCTGAATTATTCTTAGTCTGAAACTTTCTTCTAGAGCAAGTTAAAGTAAAAGATTAATGGAGCTATGTTGATTGTAGTAGTTTCTGTTTCAGATAAGTGTTCACTATGTTGGCAAGTTGAAAGAGAACGGCAAGGTAATGGACTCAACTGACAGCAGTGCTCCTTACAGGTTTCGCTTAGGTATCACCAAATGAGATAAACTAATATTTTACCAAAACATTGTTTCTGCTTTTGTATTTACATTTGTTGCTTCTTGCAGGCAAAGGGGAAGTTATAGATGGTTGGGATGTTGGCATTGACGGTAATGGCTACTAAAATTTCATCTATATTTTATAAATAAGTTTTTGACGTTTTGATTATATTGCTTTCCTTATTATTTCAAGACTCTTGTGATGTATGTGCATGATTCTCAGGTATGCGAGTTGGTGAAAAAAGAAGACTTGTTATACCCCCGTCATTGGCGTATGTTATTAAAATTTAGCCAACTGATTTCCTTCTCTGCTTTTTTCTTTTTTGGTTTCTGACTTGCTGTACATGCTTGCAGCTATGGGAGTAGAGGAGATGGTGCAGGTATACCACCAAACTCATGGCTGGAATATGATATTGAATTAGTGAAAGCTCGTTAAGCGATCAATTGATCAATTCTACAGCGTTTGGTACCGGCCTCTGTATTTTGTTTGGGCATATTGTCTTTTTGAGATTTTTCTCCCCTTCTCATCAGTACGAGAGGAAAAAAAATGAGGGAGTAACAACAGGGTTGGGTATGGTTTTCAAATGTATGGGAACGGCCTCGAGATTTTGCAATAGAAAGCTGGCTTTTGG

>MdFKBP53a

Not found

>MdFKBP62a

CTTTTCTTTTCTTCCACCTCAAGGGCATTTTGCTCCACCATTTATCGCAATGAGGCCGCCACTTCATGTTGAGCGGTGTAATCATCATTTGCCTTGCCATTTTCCTTCAACCTTCGCGCCTTGTTTCGTCCCATAGCCCTAGGTATGGAACCTTCACCCGAAGATGGATTTTCTACCCTTATTTGTTGAATGGTTGGAGATCCATCTTCATCCATATCTGCAGCTGAGGATGCAGTTCCAAACACCGGCGTAGGAGCTCTATGTGGTGGATCTTTAAATAACACCCACCCTTTACAAATTTCCAAACAACTATGAAACTGAAAGGCTTTCGAGCTGCTCTTCATATACAATTCCTTTGCTTGGTGTACCTAGAAAAAAAATTAAAGAAAATTAATTTATAAAATTAAATGTAATATAATAAATATTTAAAATAAATTGTGAAAACACTTACTTCGTCGTAGTAATTAGTGTTGCTTTCATATCTACTTGCGGCTACTAACAATGCTTGATGCCATTTGTTCAAACTTGGCTAAAGATGTTTCTTCCATCTTGAAGAACAACTCTCGTGGTTTCGAATATTCATTGGAGTGGTGCATTCATAGAACTCTAAGTATTTTTTGGACACACAAGTCCAAACACCGTCATTTGTTTGACAATTCCCCCTCTCACTATCTTCTGACACCCATCTATAAGCCTTGCAAAGAGCTTCATCTTCTTTTCGGGTCCAAGCCCTACCTTTTATTGCAGACGAGGCCATTTGAAAATTATTGAAAAAATTGAAGGATAGATTTTTGAAAGAGGAAAGAAAATATGAGAATTGAAATGGTGTAGGAATGGTGAAAATTTTGTAAGGAATGGTGAATGAATGGTTTAGCTATTTATAGGAAAAAAATTAGAATTTTTGAGTTTTTTTTTTTAAAAAGGCCCCAATAACCTAAAAAGAAAAAAAGAAAAAAAATTGAATCCAACGGTAACTAGCGCCAGTTAGCCGTTGGATTCAAATTTCTCTTTTAGCATTTCTGTCGGTTATAACCAATAGGATTAACCATTTTTCTAGCCATAACCGACATGATTAATCATTTTTCTGGCTAGCCTTTCAGCCCTTTCAGATTCCGTGGGGCCCTCTCAGATTCCACGAGCCCACTGGCTTAACCCTCAATTGGAGATGGTTTTTGGGCTATTTTCGGCCCTCTAACCCTCTGGACCTTTTGGTTGGAGATGACCTAAATGGGAGGACATGTCGTCTACGCTGAATGATTAATCCGAGCCGTCAGATTAAACCCCGGAGCACGATTAGCAACAAATCGACAAAGCACCAAATCATCTCGCAAGTCTCGCGCTGCGACCCAAAACCCTTGCAGAAGGCTCTAGAACTCGAAAAGTTCACACCATAAAAGCCTCGTCTTCCTCACTCTCACACTCACCCAATTTTCTTCAATCTCAGAATCTCNTAATACGCATTCGAAACCGTTGAATTTATCATCTCGCTTTCTCGATAAAATGGACGAGGACTTCGACATGCCGGCGGCGGAGGAGATGAACGAGGACTTCGATCTCCCCGGTGAGGGGCCGGCACTCAAGGTCGGCGAGGAGAAGGGGATCGGCACCCAGGGCTTGAAGAAGAAGCTCCTNAAGGAAGGCGAAGGCTGGGACAACCCCAAGAATGGCGACGAGGTCGAAGGTTGGCGCTTTTATCTGTTTATTTTTTGGAAATTTTGAATTTTTGGAATTGTTTTTGGTGCCTTTGGATTGGGATTGATTGGGGGNTTNTTGTGGGTTGTAGTTCATTATACTGGGACATTGCTGGACGGGACTAAGTTTGATTCGAGCCGGGACAGGGGGACTCCGTTCAAGTTCACTCTCGGACAAGGTGAGGGCTTTTTGGTACTGTTTCAGCTTCAATCTGATGAAATTTATGAACTTGATTAAGTTTTTGGTTGATTCNGATTAGTTGGTGTGTTAGTTTTGCTCAAATGTACAGTTNCTTTGCACTTGAATTTTATTGTTAGCATATACCGATGCAATTTTNGTAGAAGTGATGACAAGTACAGGCATATCTTTCATTGGGGAAAACTTTCATGCAACGGGGTTTTCACTGNGATGGTATCTCAAACTAATCACTCAATNTCATGTGCAAAAGTTAAAGCATGTGGCATTGCGTGATCAGTATGGGATACAATTACAGTGGCATCCCCGTTACAGGTCCCTCCTTTTTAATCTGTTAAAAATATGGAATTGTGTTTCAGTAGTAGCATTGATCTTCAATATGATGTTCTTTTTAAGCTTGCTTTATCAATTTTTTTTTTTTTTTTGGATCTTATGATATTATAAGATCATCGTTCAGGGTAAGCACTCTCTTACTGAGTCAATCTTGTGATGGTGTGGTGCAGGGCAAGTGATTAAGGGATGGGATGAAGGTATTAGAACGATGAAGAAGGGCGAGGATGCCCTTTTTACCATTCCCCCTGAGTTGGCTTATGGCGAGTCTGGATCACCTCCAAAGATCCCTCCCAGCGCTACATTGCAATTTGATGTTGAATTGCTATCGTGGACCAGTATCAATGACATAAGCAAGGATGGTGGTATAATCAAGAAGATCCTGAAAGAGGGAGAAAAGTGGGAGAACCCAAAAGACCTCGATGAAGTTATAGGTAAAAGCTTATCTTTGACGTTTGTTGTTGTTGGCTTGTGCACTATCTGTTGCCTTTGTCTGTTAACTTTGTTCCCTCCCTGTTTGTTATGCAGTTAATTTTGAGGCGCAGCTTGAAGATAAAACACTTGTTGCAAAATATCAAGCTGTGGAATTCACCGTCAAAGACGGTATCCTTTCTNTTTTTATATATCAATTCATTTGTTTTATTTTTTANTAGAGATTTTGGCTGTTTAATGGACTTATAATCTACTAATTATTGTAATTAGGTTACTTCTGTCCTGCAATATCAAAGGCTGTTAAAACAATGAAGAAGGGGGAGAAGGTACTATTAACGGTGAAACCACAATGTGAGTTGCCTTGAGCCATCATACTTATTTTATTGAAATTCGAAAATAACAATAGTTAAAAACGTATATCTTACCTTTATGTTGGTTACAGATGGATTTGGGGACAAGGGAAAGCCTGCCTCTGGCTCTGAGGGTGCAGTTCCTCCAAACACAACTCTTCATATCACTTTAGAGTTGGTATCATGGAGAACCGTGTCTGAAGTGACAGATGATAAGAAGGTTATTAAGAAGATCTTGAAAGAAGGGGAGGGATATGAGCGTCCAAATGAAGGGGCTGTTGTTAAATGTAAGTGTTTGTTCATTGGGATGAGTACCTTGCAATCTATAGCACGTTAGAATTTTATTAAATCGTTCTTTGATTTTGTCTGGTAGTGAAATTGACTGGGAAGCTGCAAGATGG
AAAGGAATTTTTGAAGAAAGGTCATGTTGAAGGAGAAGACCTGTTNGAGTTTAAGACAGATGAAGGTATACTTGNGTGCTTTTGTTTACATTTCTTTGTGTATTTGGCCGTCAGTGGTGCTGAACCATTGTTTGTATTGGACAGAGCAAGTGATTGATGGGCTTGATAGAGCTGTACAGACAATGAAGAAGGGTGAGGTAGCACTCCTGACTATTGCACCAGAGTATGCATTTGGCTCTGTGGAGTTCCAGCAGGAGTTGGCTGTGGTACCTCCTAATTCAACTGTCAACTATGAGGTTGAGCTTGTATCTTTTGAGAAGGTAGGTTGATAATGCCGCTCAGATTACATTGTGCACCATGTATGCCTTGGTATCAAATTATTGCGATAGTGTGTTTCGTTTTCTAACAATTTTTTAGTCTTTACGACCCTCAGGATAAGGAATCATGGGATATGAATACTGAGGAAAAAATTGAAGCTGCNGGTAAGAAGAAGGAAGAAGGGAATGCTTTGTTTAAGGCGGGTAAATATGCAAGAGCTTCCAAGAGATATGAGAAGGTAGGATGAAAAACATATTGGCTCAAGAATCTGATGGTTTGTCATTTGTTCTTGTGCTTACTGAAGCTTACCTTATTCCATGCAGGCTGTGAAGTACATCGACTACGATACCAGCTTTGGTGAAGAGGAGAAAAAACAGGCCAAGGTGTTGAAGGTTGCTTGCAATCTTAACGATGCAGCTTGCAAGCTGAAGCTGAAAGATTATAAACAGGCTGAAAAATTGTGCACCAAGGTTTGGAATATGAATTTATGAAATGCTNGCAACTCCCCTGTTTATTGTTGGCTTTTACAGTGACCAACCGTCGATATGTACACAGGTGTTGGANCTTGAGGGTAGAAATGTGAAGGCTCTGTACCGAAGGGCTCAGGCATATATTCAGCTGGCAGATTTGGATTTGGCTGAGCTTGACATCAAGAAAGCTCTTGAAATAGACCCTAACAACAGGTTTGTGCACCTTATGTCTGGATTCTCATTCCCGCGCTCAATTTGTTTCATTGAGAAGTGAAAATGCTTACGCATTGTTTCTTTGTTGAAACAGGGATGTGAAGCTGGAGTACAAGACACTGAAGGAGAAGATGAAAGAATACAATAAAAAAGAGGCNAAGTTNTACGGCAACATGTTTGCCAAATTGACTAAGTCGGACTCTCCCGATANCAATGTAAGCATCCTTTTGGTCACCTAGTGGTGTTTTTTTTTTCATGCTTTCGGGGTTTAAACGTCTGATGTCGTTTGGTGGTTCATTGTTTTTGGCTCCNCAGAAAGAAGCTGCTAAAGATGCCGAACCCATGAGCGTAGACAGCAAGGCATAAGGGGATTGCATCGCATAATCGGCNCTCGACTTCATATTGTGTCATTAAGGAGTAAATCCCTAGTTTTTTTGAGCTGTAGGACTACTCGGTATTGGCACAAAGTTGTAATTGACTTGCTTTCTTCACCTGGATTGTGAACATGGGATTTTATTGGTAATTTTCAGGAGTATTAAGGGCATTCTCCCCCAAA

>MdFKBP65a

ATTATTATGATAATTTTGAACTTTCCCCAATCGCCTAATAATAGTAATTTTTTTTTCTTTTAAATTCTTGGATAGAAAATTTAATTGCTAATTCAAATTGAAATTTAATTTTGTAGATAACAAAGTGTAAAAATACAATAATTCTGTAAATGACGCCTTATTGCAGTGGAAGAAAATATGCATTCTAGTACAAGTTTGATCTCCATCGATTCTCCTTCCCTAACATTTGATAATTTGATCTATTAAGTTCCAGTTTGGTATTGCTTGTTTTCACAAAAAACCTAATCCATTTTAAAATTAAATAGTAAAAAAAAAAAGTTTAATCTACTAAGTCCCAATTTGATACCGCCACAACCATACACACCATCATTACCATTACAACTGCCACTATCATCGATACCATTACAACTACCACTATCATCGATGTCGCCACCCCCCACTACCACTACAATCACAATTGTCACCTACCATTGTGTTCTTGTATTTACCAAGTCAGAGTTAAATAATTAAACGTCTTTCAATTTGTGTAAAAGTTTGTTGAGATGATCTTTCAATTATAACCTAAAATATATACGGTTCAAATTATGAACCGGATTGGAGCCCAAACTAATTTTTTTATATTTATTTATTTTTAATGGAATAATTATTTTTTTATTGCAAGACTGTATCTTAACAAAGTTCGTCAAAAATGGAGATAATTCTGGAACCATCCTGACCATGTCACGTTTCAGAACTCTCTAGACTCTCTCTCTCTCTCCTCTGCTTCTCTCTCTATAAATTCCATTGGCAAGCTCTCTGCATCTCCGCAATTCGAAAAAGCTCTGCATTCTCTTCACTGCTCTGTTTCGCGCTTGCTTATTTAACATCAAGCTCCCACTGCAATCCGGTCAGGAAGTTGCCATGGAAGACGACTTTGATTTCTCAGCTGCCACCAACAACCTCAACGACGCCGGTGACTTGGACCTTCCCGACGCCGAGGACGACGCAACTAGCCCAACTCTCAAGGTTGGTGATGAGAAGGAGATCGGGAAGAACGGCCTCAAGAAGAAGCTCCTTAAAGAAGGTGAAGGCTGGGACACTCCCGGCTCCGGCGACGAAGTTGAAGGTATAAATATATATACATACACACGCGCGCGCATACATACATATAAATATATGTGTGAGGTTTGATGGCGAGCGTTGGTACTAATTGGCTGGTGATAATTTTGCAGTGCATTACACTGGGACTCTGCTGGACGGAACGAAATTCGATTCGAGCCGTGACCGTGGGACCCCTTTCAAGTTCAATCTCGGACAAGGTGATTGATTCATTTCCACTTTATCATTGTTTTCAATTGTGTTCATATATATATATAAAGGGCGACTATAAGCCAACAAGGCTCTCGCTTTGTGAGGGTTGGGGAACATTTATCGCAGCCTTACTCTTGCTTTGTGAAGAGGCTGTTTTTGCGGCTCAAATTCGTGACCTTTCAGTCCCAATGGAGCAATTCTTGATTCAAGTCTTACTGAGCCCTTTTTTGGGTTATGATTCAATTGGTTAATTTCAGGACAAGTGATTAAGGGATGGGATGAAGGAATCAAAACCATGAAGAAGGGCGAAAATGCGGTTTTCACCATACCGCCTGAGTTGGCCTACGGTGAGTCTGGATCCCCTCCAACGATTCCTCCGAATGCCACACTTCAGTTCGATGTGGAGTTGCTTTCCTGGACGAGTGTGAAGGACATTCTCAAGGACGGCGGAGTTATGAAGAAAATTATCACCGAAGGAGAGAAATGGGAGAATCCGAAAGACCTTGACGAAGTGTTTGGTACATTGTCTTTCCCACACTATTTTCCAATGACTGAAAATTGATGAATGGCATTGAAACTCATTGGTTTCTTACACAATTCCTTGGTGGTATCTCAGTTAAGTACGAGGCTCGGCTTGAAGATGGGACTCTTGTTTCGAAATCCAATGGGGTCGAGTTTACTGTCCAGGATGGTTAGTTTCATGGCCTTATCTCATATGTTTACTTGCGACGCAAAGAATCCTTTTTTTCCGAAGAATCTGTGTGTGAAACCTCTTTGATTGCAGGCTATTTCTGCCCCGCCTTGGCAAAAGCTGTGAAAACAATGAAGAAAGGCGAAAAGGTCCTTTTGACTGTAAAGCCACAATGTGAGTCCTGTGCCCATATGCTTACTTCTTGATTGCTTAATTTATTGAGAGTATTCAAATTTTAATTCACAAGATGAAAACTTAACGGCTATAATTTTGTAGATGGATTTGGAGAGGCGGGCAGGCCAGCTATAGGGGATGAAGGCGCAGTGCCGCCAAATGCGACCCTTGAGATAGCGCTCGAGTTGGTATCGTGGAAGACGGTTTCTGATGTTACCAAAGATAAGAAGGTCCTCAAAAAGACATTGAAGGAGGGAGAGGGGTATGAGCGCCCGAATGATGGGGCGGTTGTTAAAGGTAATACTAGCGATGCATAAAATTTCGTAGGTAATTACATTCTTAATGTTCGCGGATGTTAAATTTTCTGTATTTGTTTTGGTCTGGCAGTGAAACTTGTTGGGAAGTTGCCTGATGGCACGATCTTCACAAAGAAGGGCCATGATGACGAGCCATTCGAGTTCAAGACGGATGAAGGTAATCATGTCCTTTCGACATCGTTTCTCTGAAACTATTGAGTTAAAATTCCTAAACAGGAAAACTTGAGACGTATTGATTGTTTGCAGAGCAAGTAATTGAGGGACTCGATAGAGCTGTGAAGAACATGAAGAAAGGTGAAGGGGCTCTTGTGACCATTCAACCAGAATATGCATTCGGGTCGACCGAATCAGCACAAGATTTGGCTGTGGTTCCTGCAAATTCGACGGTCCATTATGAGGTGGAGCTGGTCTCCTTTGTCAAGGTAAGTATGATATGTCCCCCTCGATGCAGTCCCCGTTCCCCGTTGTGAATTAAGCCGGACTAACTTACTTCACTGGTTGCAGGACAAGGAGTCTTGGGACATGAACACACAAGAAAAAATCGAAAGCGCTGGGAAGAAGAAGGAAGAAGGGAATGCACTCTTCAAGGCTGGTAAATACGAAAGAGCCTCCAAGAGATACGAAAAGGTATATCCGGTGGTCTGGCAAGAGTCTTGCCTCTTGCTTCAGTGAACAGATTTTACTTAGACGTTTATGGCAACTCATAAAAACCTCTAATTTTCGGTGACAGGCTGTGAGGTTTATCGAATATGACTCCACTTTCGGCGACGAGGAGAAGCAGCAGGCGAAGGCGCTGAAAATAACCTGCAATCTGAATGATGCTGCGTGCAAGCTGAAACTCAAGGACTACAAGCAGGCTGAGAAGCTCTGCACAAAGGTACAAGGCTCGCCGGTTTCCCATATCTCCGCGCAAGTGCGAGGGTAGCGTAGTTTGTTTCTCTTCTAATGTGTGAGAGCATTGATATGCAGGTTTTGGATTTAGACAGCCGGAATGTTAAGGCTTTGTACAGGAGGGCGCAAGCGTATAT
CCAACTTGTGGATTTGGACTTAGCAGAGCTGGATATCAAGAAGGCCCTTGAGATAGACCCCGACAACAGGTTCGTTGATGAGTTGTGTCGTTTTTGTTCGTTTGAGATGGTTCATTGATGAGTTGTGTCGTTTTTGTGTTTCGTGTGTCGATTAACTTGTGTGGGATTGCGGTTATGTGTTTTCAGGGATGTGAAACAGGAGTATAAGGTATTGAAGCAGAAAGTGAGAGAGTACAACAAGAAGGACGCGCAGTTCTACGGCAACATATTTGCAAAAATGAACAAGCAGGAGCCGGTGCCGATGACCATCGACAGCAAGGCTTGATTGAGGTTGTCATGTAATTGAAACAAAATTTGAGGTTGTGTGCATTTTTGAACAAGATTAGTAATGTAAAGCCTGGGATCTGTCAAAATGTTTGTGGCTAAATGTGCTTCTGACAGTTAAAAAGTGCTTGTAAATAAAAGTGCTTTTGCACCATATAAGCGTATTTGAGAAGTGTTTGCTTCTATTGTTTTT

>MdFKBP65b

GTTCCGAACTTTCGCCTTCCCGTATCACGACGAAGAAATTGAAGCCAATGACAATTGATACTTTCACAAAGTCATGCACATCATTTAAAGCTTTTGATAAAACCGAAATTTTATTTAATGCTCTGGAGCTTTCTTGTGATGTCCACTTCATTTAAATCGTCACGTAGATTCACAAAATATCTGTGGGCTTTTGGCCCACCAGAATATATTGTTATGAGCATTACATCGCAGTTTTTTTGGACTTAGCTCCAGTTTTGGGCTCATAGCTTTTGAGGTGGCAATTCGGTTTGCTGGTCGGCTTTATCTCTTCTCGAATTCCACACAAATACGATTACGGTTAATTTATGGTTGTTAATTAGGTTAATCTGAACTTAACATATGATTAATTGGATTACTTGTCACAAATAAAATAAAGTACATGTCCACATCAACTAAAAGAGTTAAACTTGTCATTTGAAAAAAGAGTTAAACTTGTCGATTTAACGTGAATTTAAATGAATAATCTCTATATTATTGTAATTTTATCAAATCTCTTGATATGCGTAGTCATATAATTTTCAATTTTAATTTTTCATTTCGTTTGGGAAAATATGAAAGTTAAAAGCTGAAAATAAAAGATAGAGTACTTATCATACGAATCTGTTTCAGTGTTCTTTTTATTATTTTTAAAAAAATTAAAAATAGTTCTCAAACAAAGTTAGAAGTTATAAAGAGTTATTTCACGTAACCGAGACATGTTCACGTCCATAATGACAAGTACCATCGCTAACAACCTAATTCTTGTTATTTCGTATTCGTGTCGATTTTTAGCACTGTTCTAAAAAACGGCCTAGGCAGTGGTGAGCTGATATGTTTTCTTGCAAATCGGTTGGAAAAATCGGATCGGCTCTAGGTGGCTGCCTAGGCGGGGTAGGCGGCTAGGCGGTGGTAGGCGGGGCTAGGTGAGGCTAGACGGTTATTATTTTTTTAATGAAATTAAAAATAAAATAAAAAATCCTATCTAAGTTTAAGTGGTTTAAAATTGTGAACTTGTTGTACATTTATCATCATACAATACTCCTCACTATGGTTATATGACATATATGCTTAATGTGTTTTTAAATTTTGGACTTGTTGGATACTCTTTTGGCATTTTATCATTTTTTTATTATCTTATCCATGGATTTTATACAAATATAATTTTTTGTAAGTGTTAATATGCACTTATTTACAAGATATACAAGAAAATTACCTAAATCCGCCTAGGCCACCTAGGATCTATGCTACAGCTCGCCGCCCGACTAGCGCCTAGTGTTTTTTAAAACCTTGATTTTTAGTCAGATTTGTCCTTGGGTCCAAAATCACCCTTGCCTCAATTTACTAAAACGCCCTTACATTTATCCCTTCCATTTGCCACGAAATCATCGTTCCCGTACCGCCACATAACCGCCCCACCGCCGAGCCACACTACTCTAACTCACAGTTTCCCTTGCTACCGTCGTTCGGAGTCCAGATTTTACCATGGAGGTTGAGAAAGGCACAAACCTGTCGGACATCGAGAACGACCTCGACGAGGAACCCGGCGAGGTGATCGAATCGGCACCGCCTCTCGAAGTCGGCGAAGAGAGAGAGCTCGGTAGCTCCGGCATCAAGAAGAAGCTCCTCAAGCGCGGCCATGGCTACGAAGCCCCTGAGTTCGGCGACGAAGCCACTGGTAAGTGTTTCTCCAGAAAACCCTTTTGCTAAAAGCACTTGTGATAAAAGCATGTTGGAATTTGATAAAACCCATACCTGCTGCTTGTACAATCGAGAAGCATTGCATTTTCTGCTGAGGGCTTGTTTGGTAATCCTTTTGTAGGAAGCATGTTGAAATTTTCGTGAAAAATTCAAGTGCTTCCTGAATCCTGAAGAACCAGTTGGAGGCGCTTCTTAAGTTTTTTCTGGCTAGTCAGAAGCACAATTTGTTACCAAAAAGCACCTTTGACCATTCAAGAAGCAGTGCCAAACAGGCTCTAAACATTGTTAGACGTGCTTTTGATCATCTAAAATTGCTTTGACCATTCAAGACGCCCTTAGACATTTCTTATCTCAATTAATAATATTTTAGAGAAAACTGATTGTGGAAATTTGGATTTAGTGCACTATGTTGGGACTTTGCTTGATGGGACGAAGATCGAGTCCACTAGAGATGGAGACGATCCTCTCACTATTAAGCTCGGTGAGGGCCGAGTGGTGAAGGGTTTGGATTATGCAGTCGTGACTATGAAAAAGGGAGAGATTGCATTATTCACATTACCTGCTGAGTTGGGCTATGACAATGCTGCTGTTCGATWTGAAGTTGAGCTCGTTTCATGGATTAGGGTGGTGGATTTGAGCAGAGATGGTGGGATTGTTAAGAAGATTGTGGAGAAAGGAGAGAGGAACGAGCTGCCTGGTGATCTGGATGAAGTTATTGGTACTCTGATTCCTCATCATGTAAGCATGTAATTACTGTAAACCTCAATTTTAATGTTTTTATTGGAACTTTTGTTGCTAATGCAGTCAAGTATCGTGCGGCGTTGGCTGATGGCACTGTTGTTGCAGAAACGCCAGAAGAAGGAATTGAATTCTATGTGAAAGATGGTAATTTTTGTGTGGTTTCAATGTGGAAAGCAACTATGTTTGAGATGTGCCGAATTGTGATAATTGAATAACTCTGTTATCTGCGTGTGTTTCAGGTCATTTTTGTCCAGCATTGCCRAAAGCGATCAAGACAATGAAAAGGGGAGAGAAGGCCAAATTAATTGTTCAGCCTCGGTGTATGTCTTTGTATTCTTGTKATCACACTGCTATTTTAGAGAAGAAAAAATTGAACCTTTTAACCATGAAATATTTTAAATAATTGTAGATGCCTTTSGAGAGGAGGGGAGGGATGCGAACGAGGGGTTTAATTCTATCCCTCCAAGTTCTGTGCTCAAYATTGACACAGAGTTGGTGTCTTTCAAGCCTGTTATTGACGTTACAGGTGATGCCAAGGTACTAAAGAAGATCTTGAAAGAAGGGGAAGGCGCATGGACTGCTAATGAAGGTGCAAGTGTTACTGGTAAGTTTATTGAGAYGCCCACTTTTATAGCAATRTTACTAGAATCAYGACATAAGTGTATAGCTAGGCTTGTAATTCCTTGCATTTGAAACATATAATTAACCTATGACAATCACAGTTAACTTAAAACTGCTATACAATGTCCAACTGTTGCAGTTAGCTATATAGCTAGGCTTGAAGACGGCACTGTCTTTGACAAAAAGGGAGTAGATGGAGAGCAGCCTTTGGAGTTTATCATAGATGAAGGTCAGTTACTCCATTGCATCTCTTTTTTCTGTTATTTGCATCTTTTTGCCTGATAATCACAAGAAGAAAGCTCAAAAGTAGTTGTGGCTAATATCTGCATCTTACTATCTGTAGAACAAGTGATTGCTGGTTTAGACCGAGCAGTTGCAACAATGAAAAAGGGAGAGCTGGCAATACTGACTATACATCCTGATTTTGGATTTGGAAGCGTTGAAGTAAGGCGGGACCTGGCTGTTGTACCACCATGTTCGAATGTCTTCTTTGAAGTTGAAATGTTAGATTTTATCAGGGTAATGACCATTTTGATGAACTTGTGGAATTTGCAATGATAATTTTCTAAGATATTCATATTTCCTAAACTTCCTACAGACATACTTTGGTAATTTGGTGTCCTTACTCATCATATATACAGCATAAGCTAGAAGGCACATATTTTCTTAATTTCTCTCGCAACCATTGCTTTCTCAATTATTACTCTCTAATTTTCTTTAATGAAAAGGAAAAAGCACCATGGGAAATGAGTAATCAAGAGAGACTTGAGGCAGCAGGAAAGAAGAAAGAGGAAGGCAACCTTCTCTTTAAAAAGGGAAAGCTTCAACAAGCAGGGAAAAAATATGATAAGGTAACGGARTGCGAATTTTTATTCCCAAACTAACCATAGTTTGGTTCAATTTAAACTAAATTCATATTTATTTGCTGCTTTCCTCCTCTCAGGCTGCTGATTATGTTAGTGAAGATGGAAACTTTGGGGATGATGAGTCGAAGCTAGCTAGAACACTGCGAATGTTATGTTGGTTGAACGGTGCAGCATGTAGCCTAAAACTAAATGACTTTCAGGAAGCGATCAAGCTATGTTCAAAGGTAAGCTGTACACAGAAAGAAAAACATGATGACAAAGTCTCAGCTTCTTCTAGCAAGAATAACGTTCTACACGTCTCTCAATGTTCAGAGATAAYGTTGAGGCAAATCTCATCATATTTTCTGAATTCGATCTTGTACAGGTACTAGATATCGAGTTCCACAATGTAAAAGCCTTGTACAGACGGGCACAAGCGTATATGGAAATTGCAGATTTGGTCTTGGCTGAATTAGACATCAAGAAAGCTCTYGAGGTTGATCCTCATAACAGGTAACTGAAAAACTGATCTTTGCAATCCCGTATTCCCGATGTACATTTGTTTAAAGCGACACGTACTCCGTGGAGCATTATACGATTAGTATTCTTTTTCAATTTTCCAGGGAGGTCAAGCTGATTGAGAAGAACTTGAAACGACTTCAAGTTGAAAGCGACAAGAGGGATGCTAAGCTCTACACAAACATCTTTGGACGAGCTACAAAGGTGAATATTGTTTTTTATGTTTCATCAGCTCTGAGTAATTTCAGTGACAATATTGTAACTGAAATTGGCCATCAAGGTCCATCTTAACCGAGATTCTTTACTGTAACGGCAGAAATTGAAAGTCGAGGACGAGAAAAGATGAGGCTGTGGCGMTGTAAATGGTTGCTGATACACCGGTTTCTYCCTCGCCTGAAAATGAAGTGGTTGATTCTTGTAAAAATGTCTGATCATGATTCATGATTCATGATTTGTGTATGCGTAGAGTAGTGAGAGAGACAGTCGATGAAGGTACGGGTTATATAAACTACATCAATGTA

>MdFKBP72a

CATATATTTTTTTTCAATTCAATTAAATTCATGCATATTCAATATGATTCATAGCAATATCAATTCACATAATTTAAAATTATGAGTATAATGCATACACAATTTATGTAGAATCTAAAATTTGAAAAACGTAAAGTTGGGTCATGCATTATGGTGAATGTTCATGCTATCAGGGCTGCAAAAATATCGAGATAAAAACCGTTGTTTTGGAAGAACCTGACTACGTGATGATATTGGTGAACTTGTTTGATGTAGATTTTTTTTTTCTTTCAATTTTGGCTTTCATCTTCAAGCTTGTATAACATTCAACATAAGAAGAAAAATAAAGTGAATCAGTAAAAGTATATGAATTCAATAAAATCAAAATTGAATCAATTAAAAATAATTGTAACGTAATGCTCTTCTGATTGAAGATGAATAAATTCTCTTTAGCACAGCGTGAAGAGTGTGCTAATAACGTGTTGTGGCCTATATTTAGTAGAGAGGTGCGGCAAAGGGAAGAGAGAGAGATTGGAGAATAAACTTGAGGAATTGTGTGTTAATTCCCCAGTCCTTATGCCTTTATTTACAGTAATAAGGATGAGAGAAACTTGCTCTCCAAGTAATACAAAGTATATTAGAAAATATATTTTAGATCCCAAATGATTCCTACTTTATCTCGACATAGGATTTACACAATCATATATTTATAAGAACATATGTCACATCAGAAAATGAATTATTGGGTTCCTTAATTAGGTAAACAACATTTTATTTTTTGGTGGATGATTAGGTGAACAACAATTTATCACATGTAACCACATATCACTCTGCCACATGTCGAATATATAACTAAGCTTACAAGCCGCCGGCCTAGGCTGCTCATGTGGCGCCAAACACATTCCGGACTTAATCCTCAGTGCGAGTCACCACCGTATCGTCTCTAGGACCCAGCAACCGCTCGGTAAATGGTAAATCCAGGTACCGGTTACCGCCGGTAAAAAGTCGCTCTTCGTCGTCTTCCATTCGAGTAGTTCGATTATTTGTTTAGAAGTGGGACTGGAAAGCTGTAAAAATTATCAACCCAGGCTGCTGAACTGGAAGCTTTGAAAATCACGACTCGTGAGTCAACTCGGTCTGGAATCGTCCTCGATCGTCCGAATTTCGGGTAAGCCGAGTGAAATAACGAAGGTAATTGAGTGTACGGAATCCCCGCTCTTGCTGGTTGAGCTGAGAGGAAGTTTGTTTCTTCATGTAGGGCTAGGGTTTTGACGTTGATCGGCGATGGCTGTTGAAGAAGGTGTCGAGCAGGTAGTCGCGCCGCCGCAGAAGGCGAAGGAGCCTTCCGAAGATGAGAAGCGGTATTTTGTTTTCAATTATTTTGGTTTTTTTGTGTGGAAATTATCAGTGAAATGCTGTATTTGAATTTGATTGATTTTGTTTGATTTGTGACGATAGGAGGAAGAAGATAGCGGCCGGAGCCTTGATGAAAGCGGTGATGAGGCCCGGTGGAGGCGATTCGACACCTTCAGATGGTTATCAGGTTAGAGCTTGTGTTGCMTTTTGATAAGCCATTACTGCAATTGAACTGTGTCAAATTAACAGTTTTGGTGGATAACGTTACGGAAGGAAATGGCGAAAGCTAAATTGCATTGTGCATTTTGCAGGTTGTATATCATTCCACTGTTAGAACATTGGACGGAGTCATTGTTGAATCGTCCCGATCGGAATATGGAGGCAAGTCCTTATGTTACTAACTAGATTTTAATATGTATAGAGAYTAGTGGACTTTCTAAATAACACTTCTTATATGTTTTCCCCCTAAATTAGTTTTTGTGTTGTGGTAATTCTATGATTTTCTAAAGTTGTCGAAACCTAATTTTATTTCGCTTTTGAGTAAAATGAATTTCCATGTTAAAATGCTCTGTTATGTTTGTTGAATTGATTAAACTCTTATTAACAGGCAAGGGCACTCCTATACGACATGTTTTGGGAAAGAGCAAGATCATAGTGGGATTACTCGAAGGAATTCCAACAATGCTGAAGGGTGAAGTTGCGATGGTAATATCTTCTTGGTTAAGTAAGCTTTATTTTTGCCTATGAATGTGTGCCTTTGGCTCATATGCTTTAAAGACTGTATTTTTCCTTGTATCAGTTCAAAATGAAACCTCAAGTGCACTATGGTGAGGAAGATTGCCCTGTTTCAGCACCCAGCGGCTTTCCCAAGGATGATGAACTTCATTTTGAAATTGAGATGATCGATTTCTTTAAAGCCAAGGCGAGAATTTCAAAACATAAATTTTATGGTTGAAAATCCTTGAAGTACAATTGATTATGGTGATCAACTACAGTACTCATCCTCTTCCTGACCTCAAATTGGTTCTGATCTTATTTATTTCATTTTGGCTAATTTTCAGGTCGTTAGTGATGACTTGGGAGTGGTAAAGAAGGTACGGGAAATAATAAATTATTGCACATTCGCTTGACTTAGGAGCATTCTGTTAAACTCAGAAACGCTGTCAGAATATTTATTCAATTATGATTAATAGTGTCAATCACAATAATCTGTGTAGGGACAAATTTTTCTTTACCTTCCTCCAAACTTGAGTCATTTTTCATAATAGATTGCCCATTAAAATTGGCAGGTAATAACTGAAGGTCAGGGTTGGGAATCGCCAAGGGAACCTTATGAAGTAAAAGCCTGGTAAAGACCAATTTTCCCCTTCCCAATAAGTATTCTATTGCTGTAATGAAATTAAGTATGAATGTGTGGAAACTTTCTAAGGGAGGTATATGGTTGTTTTCAGGATTTCAGCAAAGACAGGTGATGGAAAAGTGCTTGTTTCATGTACTCAAGGAGAGCCATTTTTCTTTAATTTTGGAAAGTCAGAGGTTGGTAACTGATTTCTCATTACCTTCTTTGTTTGTGTTGGTTTGTTCATTTGTTTTGTTTCAGGTGACATCGCTCTTAGCTGTTACTTATATGCTGGATTTAAATCGCATTTTTGGTGCTAATTTTGTTCTTTCATACACTAAAGAATACATTAAGTCTAGAAGTAAAATAAATTAAGACTAGAAGTAATCAGGCCACAAAAGTGTTTATGTTGAGTGGGGAAACAGTCAATTTTGATATTACTTTAACGTATTATGTCTTATTCACCGCAATGTCTTTGAATATAAACTAGGTACCTAAGGGTCTTGAGATGGGAATTGGTACAATGACACGGAAAGAGAAGGCAATAATATATGTGACCAAGCAGTACTTAACTCCATCTCCTTTCCTTCCTGTGGTAGAAGGTGTTGAGGAAGTTCATTTTGAAG
TGGAGCTTGCCCACTTTATTCAGGTGTGCAAAGTTCATCTTTATCATAACTCAAGTTCCCTCTCCATGTATCTTTGTTTTCTCGTTTTGTAATAATTTGTATAGCAAAGTTGGCAAGGATATGCCTTTATGGGGTACTCAAGTTCTGATCTAGCTTTATGGTTCTGTATTTTCAGGTGCGTGACATGCTTGGTGATGGGCGCCTGATAAAACGTCGTATTCATGATGGAAAAGGTGTGGTTCAAGAATTTTATGTTTCCAGAGAATTTAGGGTTTCTTAGAAACATAGATCGGGGTGTGCTACATGATTTTCCAATTTTTTTTCATGTCAGATTTTCCCAATTGAGTCATTGACTCTGGTCCCTTTCAAATGGTGGTTTTTATGGTCTTTTCACGGATCTTTTATCGGCATATGTTATATGCATGAAGCATAATGTTTCTATTATATTTCTTGAATAATTAGGTGAGTTTCCTATGGATTGCCCTCTTCATGACAGCCTACTACGTGTCCATTATAAGGGTATGCTTCTTAATGAGGAAAAGACAATCTTCTATGATACAAGAGTTGATAACGATGGTCAACCTTTGGAGTTTTGTTCTGGAGAAGGCCTTGTGAGTTGTCCATAAGCTTTAAGAAGAATGTTTGGCTTGAAGTCTTCATTTTTTCAGTTTGTGCAATACATGTGTGTGTGTGTCTGAGGTCATATTCTAAGGTCAGTCCGATTTGCTTGATGCAATTGGAGCTTGGTGTTTACCTTGTTCTATGTGAATGGTGGTTAATTAGAAATTCAATGCAGTGAAAGGTTTATGTTGTATGATAGGTGCCCGAGGGATTTGAAATGTGTGTTCGTTTGATGCTGCCTGAAGAGAGAGCTCTCGTCACATGCCCTCCTGATTATGCATACGACAAATTTCCTAGGTTATTTTGAGAATATTTGCTTACTATTTGTGATTTCTATCGTAATTGGTAATAAACAGTATCCTAAAATATAACACCCAGACGCACAAATGGAAGTCTCCTGCCAGTGTTTCTATAGTTTTCCAAGTTCAAGTGTAACACTGCTGTTCTCTCCATATCATAGGCCTGCTAATGTTCCTGAAGGTGCTCATATTCAATGGGAAATTGAACTTCTTGGGTTTGAGATGCCAAAGGTAATCTTATGTTTTCCTTTCTTTGAAGTTTTATTTATTTGTTTGTTTATTTTTCATATAAAAATAAAATAAAATAATTGTTTTTTATATTGTTTGTAATTTGTTTGGGTTAATTGTTGAATATTTCTGGGTTATCATTTTAATTGTTCATGTTAGTGTTAACTTTCTGTGAAGCATTACTTCAATATGAGATGAAATTTTAACCTTCGAGAATTAGCTTTCAGCTTAAAGTAACTTCAAAGTGGTTGTGTACTTTGTCTTAATGATTACGTCTAATAGTCCTTGATGGAGGTAAACGTAATTATGCGCACTCTAAATGCTTTATGTTTTGATGTGAGTTACGACTAAACTATTTATACCCATGCTTTTATAAACAATTTATACACATGCTTATATGCTTTTTGTTAGCTGGACATTTATCTGAACTATGTGTTTGAGGTTTTTTCTTCTTCTCTAGGTTCCTACTGTCAAGCTGTATGCTAGCCATGTGATTAAACCTTATTCTCAAACTCATTGCAGGATTGGACTGGTTTAAATTTTCAAAGCATAATGGATGAAGCAGAGAAGATTAGAAACACGGTATGTATTATTAACTTCTAGTGGTTGTGGTATCAAACTATCAACAGCTACTGATGTATTAACACATAATCTGATTGCATCAAAGTTTTGTGTGTGCACATTTTCGTGCTTTCATGCTCATCTTCTGAATATTCAAGTTGTTTCAGTGGTATAAAGTTTGATTTTTATCAATATATAACATTTTAATAGTAGGGTATTTGAACTTTAAATCATGGGAATTGAAACTATGATTCTATATTCGTCTCAGGGGAACAGGCTATTCAAAGAAGGAAAATTTGAACTTGCTAAGGCAAAGTATGACAAGGTACTGTGCCCTTCCATGCATGCTTTACCTTTAGATTATGAGTATTTTGTAGGTGTAGGCCTTTCCTATCTTCATGATGAAGCATGTTCTCTTACTGCGGTTGTTTATTAA

>MdTIGa

TTTCGACCCGCTTTTTGCTTCTTCGGCTTTCAAGAGTGACTGAGCCAGTGTGTGTGACTATTTCAGCTACGAGGTTGAGATATACAGATTATTTCGTTGTTCAAGGCCTCACGACGTCGTTTCTGCGTTCAGTTACAGCGTCTTTGTCAAACACACGCGACACAGTTCCGTTGACGGTTTGCAACCGCATTACTTTCTTCTCTCTCATTGGAAAAATGATAACCACACTTTCTTCTGTTATCGTTCATCTTTGTTTAATTTTGTCCGTCATTTCTGTTGAATTTATATTTAGGATGAAAATTTAAAGACAAAGTCTAAAGTACGCTGAAAAGTCAAGGACCAATTGTGGGTTTGAGTATGGATCTTATATCATGCCACGTAATTAAATTAATGAAAAGTCTCAGAATCATCAGTAACGTAACGCAATGTTAAAGTTAGAGTTCTTAATTGGCAAATTTTAATTTATAGACTAAATCTAAACTAAATTGAAAAATTAGTCGATTATAAATTTGATTTCTTTTCTCAAATACAACGATATTATTGAACTAAAGAGTTATGATTGAGTTAAGTCACACAATAAGTTTCCCTAATAATTTAGTATAAAATTTGCCCTCTACAAAAGTCGAATCTACTACTTTTCACTTCTATATATAAAAAAATTACAAATGAAAAAAAATATTATTAAACCAGAATATTAAGTGACAATTCAAATAACTAACGTCATTGTGAGAATTAGGGAATGTGGAGGAACAAATTTGGTTAAAACTTTAGGAGTAATTACATTTTACACCATTGAAATTAAAGATGATTTTCAAAATGCGCTTTGAAGTTTTAATTTTTTTAGTTTACACCATNAATAGTAAAATTGTATTAATTTAACTTTTTTTAGTTTACACCATAAATAGTAAAATTGTATTAATTTAACTTTTAGTTGGATTGATTGTTAGATCCATCATTAAACTTGTGATATTGTAAACGTGAAATCCTTATATGGATTAACATGTAAGTCAGATTTTCATCACATGACAAAAAACAAAACAAAAAAACTCATCAAATTAATGAAGGATTAAATATTTAACAGAAAATGTATAAATTCATAAAAGTTTAAAACTTCATGATTTAATATGAGAAAATTGAAAAAAAAAAATGTAGCTAGCACAAATAAATTTAACAAAGACGCGAGAGCAGAATGCAACGGAAAACTTTCCTTGTTTTGTTGGATAATCCCAGTTTTGCCCTCTATCTTTCAAGCTAAAGCTGGGCACCAAGCTAGTGAGCTCAGAACGAAACCAGGAACAATGGAGCTCTGTATGGGCGTCGTCAATNGTGTCTCCATGTCGGTCGTCAGCGTAAACCCATCACCCCCTTCACGCTCTCTTCTTTCCCCAAGAAACCCCATTTCTCTTCATCCCTTCACTTCCCTCCGACCCCNCTCCTCCCTTTCCCGCCAATTCCAACATCTCCAACCGCCACCACTCTCTCGCTCGGCGGTTCCGGCTTCTCCTTCTTCTTCGACGGCTGTCAGCGACGATAAAGATCAGCTTCCGGCGGACATAATTGTCACAGAGACCCGAGAACCCAATTCCAGAGTAAAATCTTCTTAATTGCCAATTTTTCTAATTTTGATTTTAATCTCAATTTTGGTAAATATCATCACTGTCTGTAATCTGTATGCAGGTCAAGTTAAGCATACAAGTACCGCCGGAGGTCTGCGACGATTGCTACAAAAGGGTTATGGCCGAGTTCATGAAGCAAGCCAAGGTTTCTACTTTCTAGTTTTACATTCCTTCTCCATTGTTTTCATTTAGTTTGTCTGGACTTGAAGTTTAATTTATGGATATTTTGGGGGTTTTAATGTGCGCAGGTTCCTGGATTCCGGCCCGGAAAGAAGGTGCCGGAGAGTATTCTTGTGAGTCATGTTGGGAAGAGAAGTGTCAACAAGGCTACTGTTGAATCTATTTTGAGGAGGACCCTTCCACATGCCATGTCTTCGGTACGTTAAAACTGAAATATTGTTTATTTATTACAATTTATCTTCATCGTTGTTGTTAAAACATAGAGTTGCTTAAATTCGTGTACTTGCAAGTTAGTGTTTGGATTTTGCTTATTGGCAATGGCTGCAAAGTTACAATGCTGCAGCATTTTTACAATCACAACCCCCCATAACATATAATAAGAACAGCATTAATTTCTTATGATTTATCATTCTCGAGTTCTCTGTTGTGGCTGATCTGCTTTTATGGTTTAGATGACAGGCACACCTTTAAGAGACTCAGTACGTATTGTAACCAGATTTCCAGAAATGGAGCAGACATATTCTACTCTCAATTCTCTCAGGTTGCTAATTCAGATATCTTTCAAGTTACATTTTTCCACTTGCGTATTTGATATTTCTGTTAATATCCATCTTACAATGCCATAATNCTTGCAACTCTCCTCTCACAGTATNTTAAGAGGAGAGGCATGTATTGAAGGAAATTTGATCATGTCAACTGATTATGTGCCTCCACGTTTGATTTGATCGATCATGCTTGTCAGTTTGATTTGTGGTCCAGATATGAAGTCATCGTTGATATAGCACCGGAGTTGAAATGGACACCTGAGGAGGGATACAAGAATTTGAAGGTTGTAGTTGAGCTAGATAGCGACATAGATGCTCAAAAAGCTTCTGAACAAGAATTAAGGCGACGCCATAAGTCCTTAGGTGCACTGAGAATTGTAACCGATAGAGGTCTACAGGTGACTCAACTTCAATTTACTTTTGTTCTTTTCTTTTGAATGAAATTTTACTTTAATGATATGTTCATTCGCTGCTGGGATAAATTCTGCCAAAATAGTCCATTGGAGAAGTTTCCGGTTATCACCTTCAATTTTATGAATTGTCAGACACTCCCTAAACTAATTAAGCTTTATTCGTTCAATAAAGAAAGATCACTGAACAAGCTTTCTTCACTGCACCTTCAGATGCAGTGTTTGAATTCTCTTCAGAAGTACTTCTGCGTTACATTCACAATGTTTCATGGATTAGAGTGTCATATTAGTTGAGTTTCAAAATTCCATTGAAAACCCTTCTCCGACTTGTTATATTAAGTTGGTGACTGACAAAATTGAGACTCCTACAGATTGGTGATCTTGCTGTCCTTGATATATCTGCAACAACGATAGATCAAGATGAAGCAAATGTTAAGAATATTCCATCTGCTGAGAGTAAAGGTTTGGCAATATCCACTTCTTCCGTTCTATTAACAATGGCTTTATTTTTTGGTTTGGAGTGATGAAATATTATTGCATAAGAATCTCTCAACTCTATTTTCTATCTGAAGGTTATCATTTTGATACGGAAGATGGTGATAAAGTAGTTNCTGGTTTCCTCGAGTCGATAATTGGAATTCAACGAGGTGAAACAAAGTCCTTTCCACTTNTCTTTCCTGAATCATGGACACAAGAAGAGCTCCGAGGAGTTAATGCCCAATTCAATGTGAGTGGTTTCCTGTTTTTGTGGAAGTCCACTTGGTATACTTTGTCCTCACAAGACTAAATAGGTGAAAGTTCTCATGTTTTGCTTCTTTTGATTCTCCTTTAGGTTGAATGCAAAGAATTATTCTACAGAGACTTACCCGAATTGGATGACTCCCTTGCTGAAAGGCTTCTTCCTGGAGGCACCACCCTCAAACAGGTAGCCTGTATGAGATTTTAAATACTCTCAATGAAGTGATACGTTAAAATTGTACTGTCTACAACGCTACTCTGCTTCACGAATATGCAGGTCAAGGAAGTGTTGTTNCAAAAGTTCCGGGAAATGGAGCAAACAGCTAGAGAGCAAGCAGCTGATAATGCCATTTTAGATCAGCTTTGCAAGGTGCTTTTAACTTTTCAAATATTCATCATTAAGATTAGATTGATCTTTTGCAATCTTCACTTGGTGTTTGCCTCTCTGAGACAGGGCCTTGCATGGCTTCTTTTTGTATCTGTGTACAAACACACACAAATGCACCCATGTTGTTGCAAAACCATTGCAAGGAAGTTTTGTGATTACGCTGGTCAACATGTTTGCGTGAATTCCAAAAGTCCTATGTTTTGACATGATACTGTTAAATTGCTTGACAAGCTTAATTCTTTTTGGAGGAAGCCTCAATTTTCATAAGATGAATGGTCGTTTTAAATATGCATATTTCAAACAAAGAATTATGTTCCTCTCCCTTNGTATTATTTATTAAATCCACAAAGGAGATGCAGAGTGTGTTGACATGCGGACACTTCAAGTGTCCTCTTAAATGGGAACTTCNACCTTCAGAATAACTGTGTCNTGTTGTTTTTAGATGGTAGAGGTTGATATTCCTCAGTNCNTTTTTGAGGAGCAAGGTAGGCAGCTTTATGGAGCCAAACTTTTGGAGATACAGGTCAGTAGTTACAATATAACAATGTAAAGAGAATTACATTTATTGGCTCGTGGTTTGAGTTTGTGTGTGTGTGTGTTGGTGAGCATGTAGGTTCCTGCGTTTTTCTGTTAACAACTTCTATCTTTTGGTTGTCATGGTTTTGTAGGCAAGCGTAAAATTAAATGACGAGCAGTTGGCTTCTCTGTCAAGTAAGAAGGCTGTGGACGAGTACCTTCTAAACCAGAAGGAGAACATAACAAATATGATAAAACAGAGTCTGGCTGTCGGAGACATATATAAACGTGAAAATTTGCAGGTAATTTATATTTATATTAACNGATTATTTGTCCTGAACTCTTTTTTCTGTGGGTTATGCTTTGTATCTTTTTCTGCTTGGTAGCATAGGCTTCACGAAAAATAACTGNTGGGTTGCAAAACGTATTGTAACCGGAAACCTATTGTTTGTGTTTTGCAGATTTCAACAGAGGAGATTGTCAAAGAAGTTGAAAATTCAATTGATGAATTCAAACGTCAAAAACAAGATTATGATGAAGAACGTGTCAGGGGACAGGTCAGCATGTGTCACCCTGTTAGAAAAGCAGATTTCAAGTGCTTTTAGTTTCAACTGTNTNGGCTATGTGCTTTCTGTTATCTTGATTACTTGTGCTGTCTGTGATTTCAGTCTGGATTTAGGGGAAGAGAGATGATAAAGAGAGGAAAGGAAATACGTAAAACTAAAAGGATAACCTATACGAAAATGATTAGTTCATTTTTCCAAACTAAATGCTATTGATCTTCTCCCTTTCTTCTATCCTCTCTTTTCTCACATTTCTACCATTTTTTGTTATGTACCATTATTAAATAAATCAAATTAAAACAAGAAAGCTTCCTTACGGGCGCTAAATGTTTGATGTTTTTGCTTTCTCCATCTTTTACCCTTCCTGGGGTTTTGAATTATNTAATTAATCTTCCAAATTGCAGGTTCAAGAAATTTTAGAGGGAGCAAAGGTGCTTGAATGGTTGAGAGAGCATGCAGAGATTCAATACGTAACCAGGTGACGGAGAGGAGAAGCATGGCTGTGTTGGCACGCGTACAGTGTACACAATGTTGTTCAAGGCTAGTCATGTTTTAACTTTAAAGGTGATTTCTTTTGACGATGCCCAGCAGCATTCAAGCCGACATAGTTTGCAGCATGCCTAAATTTACACTAGTTGGATTGTCCGGTTGAGGGCCAATAGATTGAGAGGTTCAATTGGTTGATTAGCGAGTTCAAAGCTATTTTTCCGAAATGAAGAATTTTGTTTGTGGATCCAGTCCTAATCAATCCAATATCTGGTTTTTGAA
