# Supplementary material for: Genome-wide analyses of genes encoding FK506-binding proteins reveal their involvement in abiotic stress responses in apple
Source: BMC Genomics. 2018 Sep 25;19:707. doi: 10.1186/s12864-018-5097-8 (PMC6156878; doi:10.1186/s12864-018-5097-8)
Supplement: Supplementary file 4 — Table S4. Application of primers and sequences. (DOCX 19 kb) [file 12864_2018_5097_MOESM4_ESM.docx]

**Table S4** Application of primers and sequences

| Use | Primer name | GenBank number | Forward primer (5'- 3') | Reverse primer (5'- 3') |
| --- | --- | --- | --- | --- |
| Complete ORF | *MdFKBP12* | KY365537 | ATGGGAGTGGAGAAGGAAGTTG | TTACTGCGCACTCAGGACTTCA |
| amplification | *MdFKBP15-2* | KY365538 | ATGAGGGCCGTCGCCATCCTCT | CTACAGCTCTTCATCTTCAGCT |
|  | *MdFKBP16-2* | KY365539 | ATGGCGGTTTCTACTCTAATCCT | CTATTTGCCCGGCAATGCTCTA |
|  | *MdFKBP16-3* | KY365540 | ATGGCTTCGTCTTCCTCCACTCT | TTACTCTTCCTCAATGTCAAGG |
|  | *MdFKBP17-1* | KY365541 | ATGATTTACACGTGCTTCGCGC | TCATTGATGCCTCAGGCTGACTA |
|  | *MdFKBP18* | KY365542 | ATGGCATCAGTAAGGTGCTTGAC | TCACTTTCCGTCAGGCGGAATTA |
|  | *MdFKBP19* | KY365543 | ATGTCTTCAATCTCAGCCGTCCG | TCAGTTTGGTATGATCTTGATGA |
|  | *MdFKBP20-1a* | KY365544 | ATGAGTGATGCAATTGATTTATCC | CTATTTTGCTTTCCCTTTTCCCT |
|  | *MdFKBP42a* | KY365545 | ATGGAGGATGTTCGGGATCAGGA | CTACTCAGCCTTATGCCTTTCGA |
|  | *MdFKBP43* | KY365546 | ATGGCTTTCTGGGGAGTCGAAG | TTAACGAGCTTTCACTAATTCAA |
|  | *MdFKBP53a* | KY365547 | ATGGTATTCTGGGGAGTTGAAAT | TCAATTCACATCGAGCAGCTCAA |
|  | *MdFKBP62a* | KY365548 | ATGGACGAGGACTTCGACATGCC | TTATGCCTTGCTGTCTACGCTC |
|  | *MdFKBP65a* | KY365549 | ATGAAGAAGGGCGAAAATGCGGT | TCAAGCCTTGCTGTCGATGGTCA |
|  | *MdFKBP65b* | KY365550 | ATGGAGGTTGAGAAAGGCACAAA | TCATCTTTTCTCGTCCTCGACTT |
|  | *MdFKBP72a* | KY365551 | ATGGCTGTTGAAGAAGGTGTCGA | TCACAAAATCGCACATCCAGGC |
|  | *MdTIGa* | KY365552 | ATGGAGCTCTGTATGGGCGTCGT | TCACCTGGTTACGTATTGAATCT |
| Promoter | *MdFKBP12* |  | ATGTGTTGGGGAATGGGGAT | TTTCTCGCTGCTTCTCGATG |
| amplification | *MdFKBP15-2* |  | TCCAAGAGTTCATGTACCTTTGT | TTGCGATCTACCTCGCTCAGTCGG |
|  | *MdFKBP16-2* |  | GCAGCGGCCATAGTAGTGGTGG | TCTGTTCTTGGTCTGGAGGT |
|  | *MdFKBP16-3* |  | GGATGCCATTCGTGCAACA | GCTGGAAGTGAAGAGTTTGTAAC |
|  | *MdFKBP17-1* |  | TGTAACCTCGATCTGTGCCT | TGTATGGTATGGGGATCCTCT |
|  | *MdFKBP18* |  | TAAGGCCGGCAAGAATACCA | CCGTTGGAAATACAGCAGCA |
|  | *MdFKBP19* |  | TCTCCTCCCCTCTCATTCCA | CAGGTGCTACTCGACTAGGG |
|  | *MdFKBP20-1a* |  | CCATCCGGATCTCTGCTCAT | TGCAATCTTCAATGTTCTGGTCA |
|  | *MdFKBP42a* |  | GGTTTCATTTACGTCCAGGGG | CCGATGGGTCTTCTCAAAATTCT |
|  | *MdFKBP43* |  | ACACAACATTTTCTCTCGTCACA | AAAAGGCCCGGATTGCTTG |
|  | *MdFKBP62a* |  | TTTGCTCCACCATTTATCGCA | TTGGGTGAGTGTGAGAGTGA |
|  | *MdFKBP65a* |  | TTGAACTTTCCCCAATCGCC | GTCACGAATTTGAGCCGCA |
|  | *MdFKBP65b* |  | ACTTTCGCCTTCCCGTATCA | CGGTAGCAAGGGAAACTGTG |
|  | *MdFKBP72a* |  | ACGTAAAGTTGGGTCATGCA | CAACGTCAAAACCCTAGCCC |
|  | *MdTIGa* |  | GCGTTCAGTTACAGCGTCTT | TCCTGGTTTCGTTCTGAGCT |
| qRT-PCR | *MdFKBP12* |  | CTGAGTGCGCAGTAACATCC | ACGAAAGAGGTCACGAAGGT |
|  | *MdFKBP15-2* |  | GTTATGGGGAGCAGGGTTCT | TCATTCCCCACCTACAGCTC |
|  | *MdFKBP16-2* |  | ATCCTTGCATCTCCCGACTT | TCTCCATTTAGCAAGCCAAGC |
|  | *MdFKBP16-3* |  | AAGCGACGACTCTACATCCC | AGCTCACGTCGAAAACAACC |
|  | *MdFKBP17-1* |  | CCAACTCGTCTTGCTAATGGA | ACTGCTCGGGATAAAAGGTGA |
|  | *MdFKBP18* |  | AGGGGCATGAATGAGATCCC | ACTTCCAGCTCATCCCCATC |
|  | *MdFKBP19* |  | GGATTTCGTGCTGAGGAACC | TCGATATTTCTGGCATCCGG |
|  | *MdFKBP20-1a* |  | AGAGGGAGTTAGTTGCTGCA | AGTATGCGACCAAGTGCCTA |
|  | *MdFKBP42a* |  | TCGTCTCTTCAGGCTCGAAA | CATCTAAACTCCCTCCCGCA |
|  | *MdFKBP43* |  | GGGAGTAGAGGAGATGGTGC | TACAGAGGCCGGTACCAAAC |
|  | *MdFKBP53a* |  | GTTGGGGTTAACGGTATGCG | ACTCAAAAGTCGGAAGCGGA |
|  | *MdFKBP62a* |  | CTGCTAAAGATGCCGAACCC | TGCCAATACCGAGTAGTCCT |
|  | *MdFKBP65a* |  | GATGACCATCGACAGCAAGG | TGACAGATCCCAGGCTTTACA |
|  | *MdFKBP65b* |  | AGCGACAAGAGGGATGCTAA | AACCATTTACAGCGCCACAG |
|  | *MdFKBP72a* |  | TGCAGTTGAGAACCCCAAGA | ACAAAATCGCACATCCAGGC |
|  | *MdTIGa* |  | TGGTTGAGAGAGCATGCAGA | GCTGGGCATCGTCAAAAGAA |
|  | *MdHSFA2* | GO504821.1 | TGGTGAATGTTGCAAAGGGG | TTCACAGGTCTCCGATGGC |
|  | *MdHSP60* | CN943489.1 | AAGGACGAGAAGGAAACCCC | ACACGAGGAAGGTTAGAGCC |
|  | *MdHSP81-2* | CX023789.1 | CTTCCGAAAACCCTAGCACG | CGAGCTTGCTCTTGTCAGTC |
|  | *MdHSP90.1* | GO526831.1 | GGATGACCCCAACACTTTCG | CTGGCTTAGTCCACCTCCTC |
|  | *MdHSP70-15* | EB135841.1 | TCTCTCTCTCCTCCCTCTCG | GCACAACGTCAATACCCCTC |
|  | *MdHSP81.4* | EG631339.1 | TGATGATGACGCTGTGGAGG | AAAACTACTGCTGGCTGGGT |
|  | *MdHSP89.1* | GO563143.1 | TACTAGACGCCGAGGTACAG | ACACCTCTAAGATCCTACCCC |
|  | *MdMTHSC70-2* | CN881173.1 | ACCCTCTGTTCTTCTCGCTC | GTGATGGCTTGGCAATGCTA |
|  | *MdMDH* |  | CGTGATTGGGTACTTGGAAC | TGGCAAGTGACTGGGAATGA |
