# Supplementary material for: Genome-wide analyses of genes encoding FK506-binding proteins reveal their involvement in abiotic stress responses in apple
Source: BMC Genomics. 2018 Sep 25;19:707. doi: 10.1186/s12864-018-5097-8 (PMC6156878; doi:10.1186/s12864-018-5097-8)
Supplement: Supplementary file 5 — Table S5. FKBP fragments identified from the apple genome. (DOCX 13 kb) [file 12864_2018_5097_MOESM5_ESM.docx]

**Table S5** FKBP fragments identified from the apple genome

| Gene ID | Protein length | Molecular weight (kDa) | Theoretical pI | Chromosome Location | FK506-binding domain | complete ORF |
| --- | --- | --- | --- | --- | --- | --- |
|  |  |  |  |  |  |  |
| MDP0000159228 | 187 | 20.7 | 4.75 | chr14:6159993..6160781 | 1-34 | No |
| MDP0000120395 | 173 | 18.7 | 7.31 | chr12:22574724..22576252 | 1-64 | No |
| MDP0000125166 | 165 | 18 | 6.77 | unanchored:99741454..99743458 | 47-141 | No |
| MDP0000130405 | 469 | 52 | 8.97 | chr6:23748093..23751336 | 212-314 | No |
|  |  |  |  |  |  |  |
